# Temporal dynamics of gene expression in the lung in a baboon model of E. coli sepsis

**DOI:** 10.1186/1471-2164-8-58

**Published:** 2007-02-26

**Authors:** Hua Zhu, Yuhong Tang, Lacramioara Ivanciu, Michael Centola, Cristina Lupu, Fletcher B Taylor, Florea Lupu

**Affiliations:** 1 Cardiovascular Biology Research Program, Oklahoma Medical Research Foundation, Oklahoma City, OK, USA; 2 Arthritis and Immunology Program, Oklahoma Medical Research Foundation, Oklahoma City, OK, USA; 3 Department of Pathology, Oklahoma University Health Sciences Center, Oklahoma City, OK, USA

## Abstract

**Background:**

Bacterial invasion during sepsis induces disregulated systemic responses that could lead to fatal lung failure. The purpose of this study was to relate the temporal dynamics of gene expression to the pathophysiological changes in the lung during the first and second stages of *E. coli *sepsis in baboons.

**Results:**

Using human oligonucleotide microarrays, we have explored the temporal changes of gene expression in the lung of baboons challenged with sublethal doses of *E. coli*. Temporal expression pattern and biological significance of the differentially expressed genes were explored using clustering and pathway analysis software. Expression of selected genes was validated by real-time PCR. Cytokine levels in tissue and plasma were assayed by multiplex ELISA. Changes in lung ultrastructure were visualized by electron microscopy. We found that genes involved in primary inflammation, innate immune response, and apoptosis peaked at 2 hrs. Inflammatory and immune response genes that function in the stimulation of monocytes, natural killer and T-cells, and in the modulation of cell adhesion peaked at 8 hrs, while genes involved in wound healing and functional recovery were upregulated at 24 hrs.

**Conclusion:**

The analysis of gene expression modulation in response to sepsis provides the baseline information that is crucial for the understanding of the pathophysiology of systemic inflammation and may facilitate the development of future approaches for sepsis therapy.

## Background

Sepsis and its complications represent a complex disease encompassing multiple pathological processes, including uncontrolled systemic inflammation, coagulopathy, microvascular and hemodynamic abnormalities. The development of septic shock, acute respiratory distress syndrome (ARDS) and the onset of multiple organ (lung, liver, kidney, heart) dysfunction (MOD) are landmarks of severe sepsis, one of the leading causes of mortality in critically ill patients[1]. Despite significant progress, the pathophysiology of sepsis and its sequels are still not fully understood. One early MOD symptom associated with sepsis is acute lung injury[2]. Lipopolysaccharide (LPS) was considered the main component in the induction of lung injury in gram-negative bacterial sepsis. LPS recognition by host's Toll-Like Receptors (TLRs) is followed by subsequent activation of a plethora of signal transduction cascades in different lung cells[3], leading to the generation of a multitude of inflammatory mediators, including proinflammatory cytokines, chemokines, adhesion molecules, reactive oxygen species and nitric oxide[4,5]. This process is accompanied by massive influx of neutrophils and macrophages into the lung, and by severe damage to the lung endothelium and alveolar epithelium[6,7]. Other mediators including microbial signal molecules, complement activation products, and coagulation factors play a critical role in the pathophysiology of sepsis[8,9]. However, neither the full spectrum of mediators acting in multiple pathways nor the temporal expression pattern of each individual mediator, alone or in combination, are clear to date. Understanding the pathogenesis of sepsis has been particularly challenging as this involves multiple functional pathways, comprising hundreds of genes. We reasoned that microarray technology would likely provide novel insights into the pathogenesis of sepsis or acute lung injury by allowing genomic-scale analysis of the gene transcription in specific organs. In the past, this technology has been used to study the pattern of gene expression either on cells exposed in vitro to LPS [10-13] or *E. coli*[14], or on blood cells isolated from septic patients[15]. So far, global gene expression studies in animal models of sepsis have been limited and restricted to rodents[2,6,16]. However, mouse models do not reflect the pathophysiology of the disease in humans (e.g., mice do not produce IL-8, which is a major proinflammatory chemokine in humans). Our laboratory had characterized a baboon model of compensated (non-lethal) *E. coli *sepsis, which is comparable in nearly all physiological and immunological aspects to human disease. Similar to humans, the baboon model of compensated response to inflammatory stress exhibits two stages, each with its unique inflammatory and haemostatic response signature[17]. Baboon-specific microarrays are not available, yet, but given the close evolutionary relationship between baboons and humans, human microarrays have been successfully used to study gene expression patterns in non-human primates [18-21] and several cross-hybridization studies between these species have been attempted [22-24]. Using the baboon model of sepsis, here, we report for the first time a global analysis of gene expression patterns in the lung of baboons during the first 24 hrs post *E. coli *challenge. The purpose of this study was to relate the temporal dynamics of gene expression in the lung to what we already have learned in previous studies about the compensated response of the baboon to sublethal *E. coli *challenge. Our results provide information relating to the temporal changes of the gene expression during inflammation in response to *E. coli *infusion, which could serve as a basis for discovery of new diagnostic and prognostic indicators and biomarkers, as well as the identification and validation of new molecular targets for drug development.

## Results

### 1. Global analysis of gene expression profile in baboon septic lung

Oligonucleotide microarrays were used to study the time-course of gene expression in the lung of baboons challenged with *E. coli*. Approximately one third of the genes on the chip (7240 out of 21,423) were detectable above the background.

**Figure 1:**
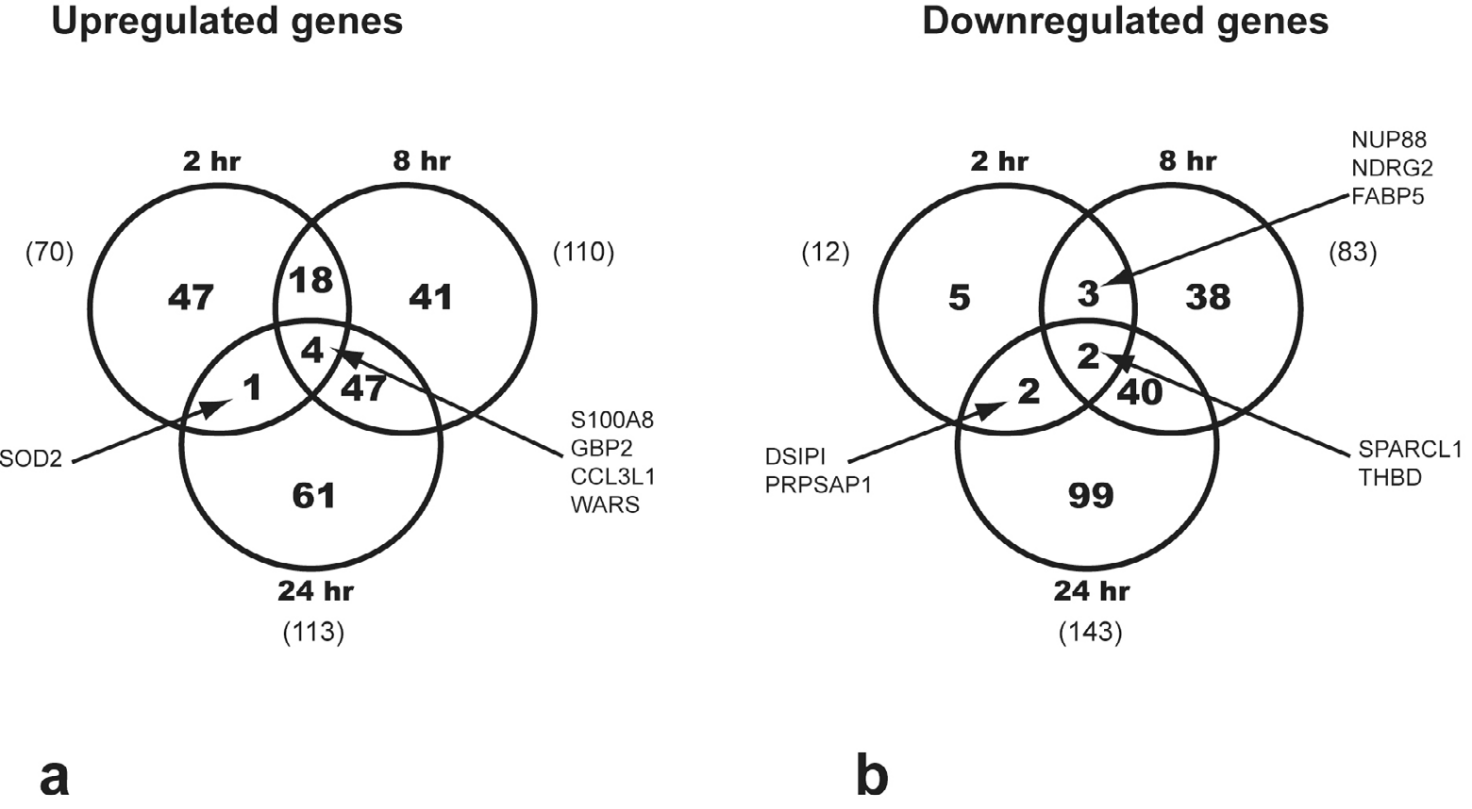
**Venn diagram of the differentially expressed genes in the *E. coli *challenged septic baboons**. a: genes up-regulated; b: genes down-regulated at three time points.

Genes up- or down-regulated more than 2 times over controls at any time-point (Figures 1a and 1b) were considered differentially expressed. At 2 hrs after *E. coli *challenge, expression of 83 genes was modulated by treatment (70 up- and 13 down-regulated); at 8 hrs, 193 (110 genes up- and 83 down-regulated); and at 24 hrs, 256 (113 up- and 142 down-regulated). Four genes (S100A8, S100A9, WARS, GBP2 and CCL3L1) were up-regulated and two genes (THBD and SPARCL1) were down-regulated at all three time points. The genes up-regulated during the time-course of the experiment are listed in Table 1, and the down-regulated genes are listed in Table 2. Most of down-regulated genes (95%) were detected in the lung at 8 hrs and 24 hrs. These genes are involved in inflammation and immune response, protein scaffolding and blood coagulation, collagen and extracellular matrix catabolism, cell adhesion and migration, cell cycle, stress response, protein transport and synthesis, energy generation and transcriptional regulation (Table 2).

### 2. Validation of microarray results by real time RT-PCR

To confirm the microarray results, the expression level of nine genes was measured by real-time quantitative RT-PCR using the same mRNA samples used for microarray analysis. The primer sequences of these genes are listed in Table 3. Microarray and real-time PCR data were highly concordant (Table 4).

### 3. Functional analysis of gene expression in lung during *E. coli *sepsis

#### Inflammatory/immune response genes

##### a. Induced proinflammatory proteins

At 2 hrs, 25 of the 70 up-regulated genes belong to the inflammatory/immune pathway (Table 2; Figure 1a). Five of the most highly up-regulated genes were chemokines (CXCL2, CCL20) or inflammation-associated genes (S100A8, S100A9, GBP2). Other genes up-regulated at 2 hrs include CXCL2, CCL20, IL1B, CXCL10, CCL2, IL6, IL15RA. Genes upregulated only at 8 hrs include CXCL11, IRF1, VCAM1, CLECSF5, GBP1, S100A9, while GBP2 and S100A8 were up-regulated at both 8 hrs and 24 hrs.

##### b. Induced antinflammatory proteins

Three TNF-α modulators, TNFAIP2, TNFAIP3 (TNF-α-induced proteins 2 and 3) and TNIP1 (TNFAIP3 interacting protein 1) were upregulated at 2 hrs. TNFAIP2 may play a pro-angiogenic role, whereas TNFAIP3 interacts with TNIP1 and inhibits TNF-induced NFk-B-dependent gene expression by interfering with RIP- or TRAF2-mediated transactivation signal. The complex acts as an inhibitor of apoptosis, has a role in the function of the lymphoid system and may contribute to the in vivo effects of TNF-α.

Two other NFk B inhibitors (NFKBIA and NFKBIE) were upregulated at 2 hrs by 6.2-fold and 2.4 fold, respectively. These proteins inhibit NFk-B by complexing with and trapping it in the cytoplasm. They may be involved in regulation of transcriptional responses to NFk-B, including cell adhesion, immune and proinflammatory responses, apoptosis, differentiation and growth. IL15 RA (IL15 receptor α, 2.2-fold increase at 2 hrs) signals through Jak-STAT pathway and plays a role in IL15-mediated T-cell proliferation and differentiation of natural killer cells. Its ligand, IL-15 is a potent inhibitor of TNF-mediated apoptosis and has a protective effect against *E. coli *induced shock [25]. IL1RN (IL-1 receptor antagonist; 2.8-fold increase at 2 hrs) is a protein secreted mainly by macrophages that acts as competitive inhibitor of IL-1β binding to its cell-surface receptor. Thus, IL1RN functions as a major naturally occurring anti-inflammatory protein.

##### c. Suppressed inflammatory genes

Decreased expression of a group of inflammatory response genes, including TNFRSF8, CXCL5, C7, HLA-DRB3 was observed at 8 and 24 hrs. TNFSF8/CD30L, a member of the TNF receptor superfamily that regulates gene expression through activation of NFk-B, may play a role in the regulation of cellular growth and transformation of activated lymphoblasts. C7 is a complement component that binds to C5b forming the membrane attack complex C5b-7. HLA-DRB3 is a component of the major histocompatibility complex class II and is essential in presenting bacterial antigen to CD4-positive lymphocytes.

#### Hypoxia, oxidative stress and apoptosis genes

Several genes that have function related to hypoxia and oxidative stress, including HIF1A, TXNRD1, SOD2 and HP, were induced during the time-course of the experiment. HIF1A (2.9-fold increase at 2 hrs) functions as a master transcriptional regulator of the adaptive response to hypoxia. Under hypoxic conditions, HIF1A translocates into the nucleus, where it binds to the hypoxia response element (HRE) of target gene promoters and activates the transcription of over 40 genes involved in oxygen delivery or metabolic adaptation to hypoxia. Upregulation of HIF1A in the lung reflects the strong ischemic condition installed in the early hours post *E. coli *challenge.

Expression of HP (haptoglobin) was increased 33.3-fold at 2 hrs (second highest induced gene) and 24.4-fold at 8 hrs. HP binds free hemoglobin released from erythrocytes with high affinity and thereby inhibits its oxidative activity. TXNRD1 (thioredoxin reductase; 2.1-fold increase at 2 hrs) is a key enzyme in the regulation of the intracellular redox environment, and protects against oxidative stress. SOD2 (mitochondrial superoxide dismutase; 12.5 and 5-fold increase at 2 and 24 hrs, respectively) plays a key role in free radical detoxification by destroying toxic free radicals produced within cells during sepsis.

Among the downregulated proteins involved in oxidation, FABP5 (fatty acid-binding protein-5) was decreased at both 2 hrs and 8 hrs.

With regard to apoptosis, both pro-apoptotic (COP1, GADD45B and RIPK2) and anti-apoptotic (TNFAIP3 and BIRC3) genes were induced in the early stages of sepsis. RIPK2 (CARD-containing IL-1β ICE-kinase) activates both pro-caspase-1 and pro-caspase-8 and also potentiates CASP-8-mediated apoptosis.

Moreover, the highest increased gene transcript in our study, IMAGE clone 2363394 (36 and 114 fold at 2 and 8 hrs, respectively) may encode a proapoptotic protein. Searches for known protein domains and functional sequence motifs in the putative open reading frame of this clone, using SMART 4.0[26,27], show perfect homology with a serine-threonine kinase, STK24, also known as STE20-like kinase 3 (Mst3), a less known protein kinase with potential role in the onset of apoptosis[28].

As a general feature, we found that pro-apoptotic genes predominate during early stages, and anti-apoptotic genes predominate during the second stage of sepsis. Five genes encoding apoptosis inhibitors (TNFAIP3, BIRC3 HIPK3, MAPK8IP1 and LCN2) were up-regulated at different time points while one gene, DAP, a positive-regulator of apoptosis was down-regulated.

#### Cell adhesion and extracellular matrix (ECM) molecules

During the inflammation, adhesion molecules are involved in the transmigration of leukocytes through the vessel wall. Beside the two well characterized adhesion proteins ICAM1 and VCAM1, CEACAM8 (CD67, a GPI-anchored protein exclusively expressed on neutrophils and eosinophils), and SDC4 (syndecan 4, a heparan-sulphate proteoglycan) were also up-regulated early in the inflammatory response. Two other adhesive molecules, VASP and CLECSF5, were up-regulated at later stages (8 and 24 hrs). VASP is an actin-associated protein that functions in cytoskeleton remodeling during leukocyte-transendothelial migration. CLECSF5 is a lectin expressed on macrophages that may be involved in proinflammatory activation of myeloid cells via TYPO-BP mediated signaling.

Decreased mRNA levels of another group of genes (CTGF, UCC1, PKP4, TNS, CD9, FN1, VCL, ITGB1BP1, claudin 4, and VEGF) were observed at 8 hrs and 24 hrs. CTGF is a major connective tissue mitoattractant secreted by endothelial cells. UCC1 is a calcium-dependent cell adhesion molecule, similar to the protocadherins and ependymins. PKP4 is a component of desmosomes and other adhesion plaques, and is involved in regulating junctional plaque organization and cadherin function. TNS (tensin-1) is localized to focal adhesions, and is thought to play a role in cell migration by linking signal transduction pathways to the cytoskeleton. CD9 is a tetraspanin family member that makes complexes with integrins and other tetraspanins, modulates cell adhesion and migration and also mediates platelet activation and aggregation. FN1 (fibronectin) is a dimeric/multimeric glycoprotein present in plasma, ECM and on the cell surface, and is involved in cell adhesion and migration, wound healing, blood coagulation, and host defense. VCL (vinculin) is a cytoskeletal protein associated with cell-cell and cell-matrix junctions, where it is thought to function as one of the several proteins involved in anchoring F-actin to the membrane. ITGB1BP1 may play a role in the recruitment of β-1 integrins to the focal contacts during integrin-dependent cell adhesion. Genes with decreased expression at 8 hrs and 24 hrs include matrix proteins (collagen VI) or metalloproteainases and their inhibitors (MMP2, TIMPs 1 and 2).

#### Regulation of cell growth, transcription and membrane transport

Except for ACVR1 (activin receptor 1B) that was induced at 2 hours, most of the genes involved in growth regulation and recovering processes were upregulated during the second stage of sepsis. Genes encoding proteins that are involved in cell cycle (CDK8, SMARCB1, STAG2), cell migration (SLIT1), cell proliferation (PHLDA2, NMB, FAP, TIE), tissue regeneration and remodeling (FGF23), and protein glycosylation (MGAT2, HPSE, B3GNT1, HSE3ST3A1, LARGE, GLANT13) were increased almost exclusively at 24 hrs (Table 2).

Most of the genes encoding for transcriptional regulation factors, including transcription factors (HOXA11, FOXK2), transcription repressors (HIC), splicing factors (PRPF4, SFRS2IP), and nuclear receptors co-activators (NCOA2, NUFIP1, NR2F2), were also up-regulated at 24 hrs. However, increased expression of transcriptional factors NFKBIA and NFKBIE (NFKB negative regulators), as well as of GTF2A1 (general transcriptional factor IIA) are detected only at 2 and 8 hrs, respectively.

STAT4, a major transcriptional co-regulator of several signaling pathways (STAT, p53, wnt and steroid hormone signaling) was down-regulated at 24 hrs.

Several ion transporters, including zinc transporters and members of the solute carrier (SLC) families (SLC25A28, SLC30A6, SLC39A8, and SLC39A14) are increased during the early stages of *E. coli *sepsis. The expression of other transporters, including SLC2A9 (glucose transporter) and SLC25A3 (mitochondrial phosphate carrier) was decreased at 8 hr and 24 hrs.

A decreased expression of several genes involved in the intracellular trafficking of proteins was observed at 24 hrs. These included adaptor-related protein complex 2β-1 subunit (AP2B1), a component of the coat surrounding transport membrane vesicles, three members of RAS oncogene family (RAB2, RAB6A and RAB36), and sortilin 1. The latter is required for the formation of a specialized storage vesicle for the glucose transporter SLC2A4/GLUT4.

### 4. Clustering of gene expression patterns induced by sepsis

**Figure 2:**
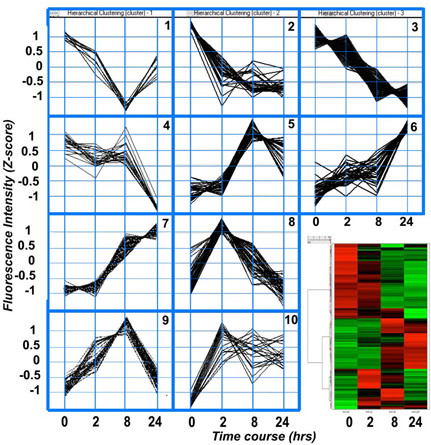
**Ward's hierarchical cluster analysis of time-dependent gene expression patterns in the lungs of *E. coli *challenged versus control baboons**. a: Differentially expressed genes were used in the hierarchical clustering analysis by Spotfire DecisionSite 8.1 (Spotfire, Sommerville, MA). Wards's method computes cluster proximity by the squared Euclidean distance between the gene cluster mean profiles. With this method ten clusters of genes with similar profiles were generated. See the Additional file 1 for a list of genes grouped in each cluster. b: Heat map of hierarchical clustering analysis. The hierarchical clustering process is represented as a tree, or dendrogram, where each step in the clustering process is illustrated by a join of the tree. Red color indicates a Z-score 1, green color indicates a Z-score equal to -1. The colors between red and green indicate Z-scores between 1 and -1.

Pathologic processes in sepsis fluctuate significantly. Understanding this complexity may allow enhanced pharmacologic intervention. Using gene clustering methods demonstrated that gene expression also changes over time in complex manner. A gross assessment of expression kinetics could be made from a heat-map representation of differentially expressed genes from all time points. Three basic expression patterns were observed (Fig. 2). About 40% of the genes were down regulated, 25% of genes were up regulated and 15% were up and then down regulated. A list of the genes presented in this map is provided as Supplementary table 1.

A more detailed analysis of expression changes was done using a clustering algorithm that identifies clusters of genes that change in expression in a highly correlated manner. Ten clusters of genes were identified based on their expression dynamics during the time-course of the experimental sepsis (Fig. 2; and Additional file 1). Gene clusters 1 to 4 contain genes that are downregulated by sepsis (Fig. 2). Cluster 1 groups 9 genes that reach a maximum decrease at 8 hrs and recover at 24 hrs. These genes are involved in cell cycle (TFDP2, GPC6), tight junctions (CLDN4), and cell structural integrity (KRT19).

Cluster 2 consists of 34 genes that are decreased at 2–8 hrs and stay decreased at 24 hrs. These genes are involved in complement and coagulation cascades (THBD, A2M), cell adhesion and migration (MMP2, UCC1, CTGF, PKP4, TNS,) and scaffolding and signaling (CAV1; caveolin 1). Cluster 3 is the largest (118 genes) and includes genes involved in inflammation (TNFRSF8, C7, CXCL5, ITGB1BP1, STAT4), apoptosis and stress (GSTM5, GPX1, DAP), extracellular matrix (COL6A1, TIMP2, TIMP3), and cell adhesion and migration (CD9, FN1, and VCL). Genes in cluster 3 steadily decreased during the time course of the experiment, except for VEGF, which increased at 2 hrs then was gradually down-regulated.

Clusters 5 to 10 include genes that are upregulated by *E. coli *challenge, as compared to controls. Genes in cluster 5 are upregulated between 2 and 8 hrs, then decrease at 24 hrs. Genes in cluster 6 are steadily upregulated during the time-course of the experiment, while those in cluster 7 are strongly upregulated during the mid and second stage. Clusters 6 and 7 consist of 34 genes each that reach maximal expression at 24 hrs. Genes in these groups have biological functions in cell proliferation and wound healing (FAP, STAG2, and FLT1 in cluster 6; FGF23 and TIE in cluster 7), signal transduction (MAP3K3, GNA14, GPR1, GNG10 and MAPK8IP1 in cluster 6; BMX, NMB and NR2F2 in cluster 7), protein glycosylation (HPSE and HS3ST3A1 in cluster 6; B3GNT1 and GALNT13 in cluster 7), blood pressure regulation (NPPC), energy pathway (NNT and GNA14), and inflammation (MD1, lymphocyte antigen 86).

Cluster 8 contains genes that peak at 2 hrs, and cluster 9 genes peak at 8 hrs and then gradually decrease. The genes in cluster 10 peak at 2–8 hrs and generally stay up for the rest of the time. Cluster 8 contains mainly early responsive genes, such as cytokines and chemokines (IL6, CXCL2, CCL2 (MCP-1), IL1B, CCL20, IL1RN, CCL4), inflammatory response genes (RIPK2, SDC4, TNFAIP2, TNIP1, NFKBIE, TNFAIP3, ICAM1, VCAM1, CEACAM8, NFKBIA), stress response (TXNRD1, HIF1A, HP) and apoptosis-associated genes (BIRC3, GADD45B). Cluster 9 contains mid-stage induced genes, including many cytokines and inflammatory response genes, such as CXCL10, CXCL11, CSF3, S100A8, CLECSF5, S100A9, IRF1, PSMB8, PSME2, GBP1 and GBP2. While the post-transcriptional effects of these patterns are more relevant for designing therapy, it is clear from this analysis that transcriptional regulation itself is equally complex, and perhaps models one causal aspect of disease pathology.

### 5. Pathway analysis

Differentially expressed genes were analyzed using pathway analysis software, which characterizes networks of genes with common regulatory and signaling capacity. This allows identification of potentially relevant molecular processes by visual inspection. Regulatory and signaling networks of genes were created from the differentially expressed genes at the 2 hrs, 8 hrs and 24 hrs time points. For each time point several major molecular pathways were identified. The lists of genes in each network and their scores are summarized in the Additional files 2, 3, 4.

**Figure 3:**
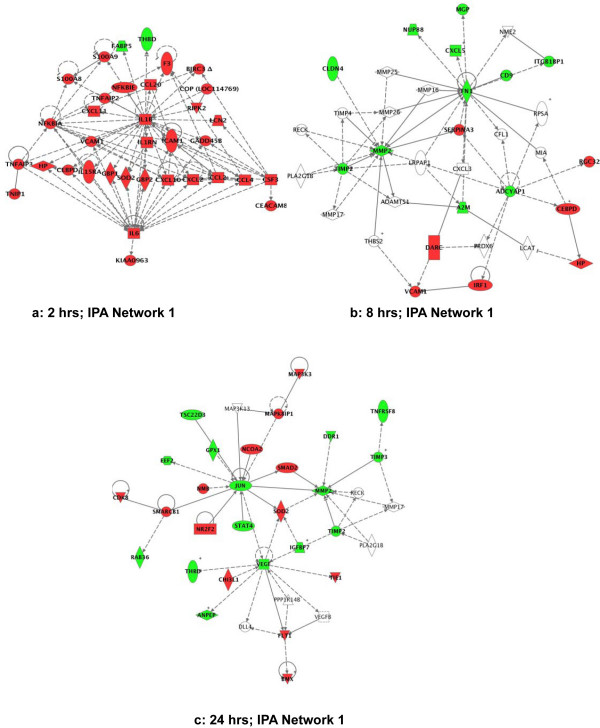
**Ingenuity Pathway Analysis (IPA) of the differentially expressed genes at different time points after *E. coli *challenge**. Green indicates down-regulation and red indicates up-regulation. In this map all genes shown were statistically different from the control. A line indicates that two gene products show binding, a line terminated by arrow indicate that one gene product acts on the other gene product, and the plus sign indicate that other networks contain the gene product. The networks with the highest score in each time-point group are illustrated. a: IPA network 1 at 2 hrs; b: IPA network 1 at 8 hrs; c: IPA network 1 at 24 hrs. See the Additional files 2, 3, 4 for lists of genes involved in each network.

Moreover, this type of analysis underscores the complex evolution of molecular processes that occurs in sepsis. At 2 hrs the most significant (score 78) network contained 35 differentially expressed genes. This pathway consists mainly of pro-inflammatory genes responsive to IL-1β and IL-6 (Fig. 3a). Three other pathways identified at 2 hours were involved in cell movement, cell proliferation and death, cell signaling and molecular transport [see Additional file 2].

At 8 hrs, 8 networks with more than 10 focus genes were found to be highly significant [see Additional file 3). These networks were associated with several functions related to tissue regeneration and homeostasis, including cell migration, proliferation, membrane transport, cellular assembly and organization. The network depicted in Fig. 3b has the highest score (26) and highest number of focus genes (18 genes; 7 up- and 11 down-regulated), and is involved in cell movement, cell-cell signaling and interaction.

At 24 hrs, 10 networks with more than 10 focus genes were detected [see Additional file 4]. The highest score belonged to a network with functions in cardiovascular development and organismal survival (Fig. 3c). Similar to the 8 hrs time-point, the major networks expressed at 24 hrs are involved in tissue regeneration and homeostasis [see Additional file 4].

### 6. Cytokine profiling in lung and plasma

**Figure 4:**
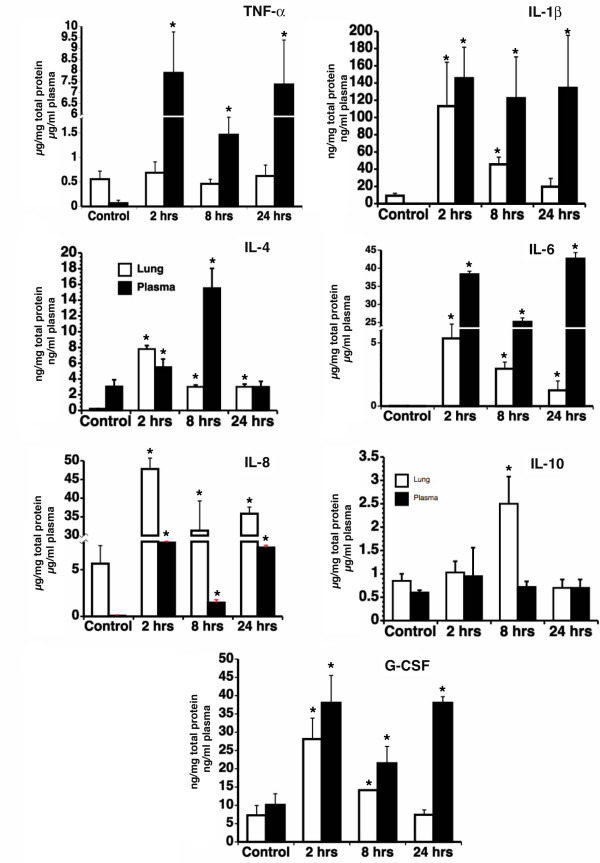
**Cytokine quantitation in lung extracts and plasma of *E. coli *challenged baboons**. TNF-α, IL-1β, IL-4, IL-6, IL-8, IL-10 and G-CSF were assayed in lung lysates and plasma using a multiplex assay, as described in the Methods section. The values represent calculated as ratio per total protein in cell lysates or per ml of plasma, and presented as mean ± SD (n = 3). Asterisks (*) indicate p < 0.05 t-test values for lung and plasma samples of challenged animals (2, 8 and 24 hrs post *E. coli *infusion) vs. non-challenged controls.

Since it has been suggested that serum cytokine levels may not be representative of local tissue inflammation, we have measured the cytokine levels in lung homogenates in parallel with the plasma (Fig. 4). In the lung, most of the cytokines assayed (TNF-α, IL1-β, IL-4, IL-6, IL-8, and GM-CSF) show maximum levels at 2 hrs after which they gradually decrease, except for IL-10, which peaks at 8 hrs. The plasma levels of these cytokines show a bimodal pattern, with a first peak at 2 hrs followed by a second peak at 24 hrs, except for IL-4, which peaks at 8 hrs. Most cytokine levels are higher in plasma than in lung tissue, with the exception of IL-8, which is over 5 fold higher in the lung than in the plasma, and IL-10, which is almost 2 times higher in the lung at 8 hrs than in the plasma. Our data demonstrate the existence of a lung specific cytokine profile, and suggest that serum cytokine levels may not be representative of local tissue inflammation and end-organ damage.

### 7. Structural changes of the lung during *E. coli *sepsis

**Figure 5:**
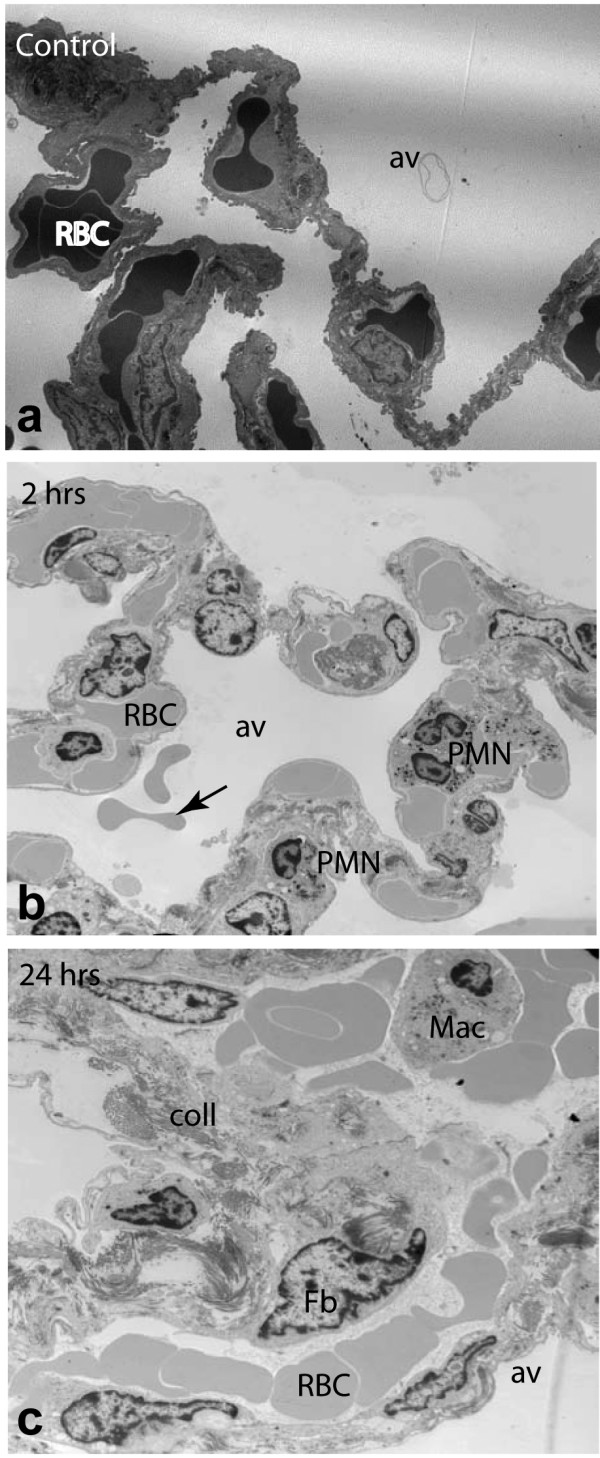
**Electron microscopy analysis of structure changes in *E. coli *challenged baboon lung**. a: normal architecture of the alveolar septae in healthy baboons; b. accumulation of neutrophils (PMN) and the presence of intra-alveolar bleeding erythrocytes (arrow) can be observed at 2 hrs; c: the increased accumulation of macrophages, fibroblasts and collagen deposition at 24 hrs. av, alveola; coll, collagen; Fb, fibroblasts; Mac, macrophages; RBC, red blood cells. Magnification: ×7000.

Histology and electron microscopy analysis demonstrate gradual alteration of lung morphology. In contrast to the normal architecture of the alveolar septae observed in healthy baboons (Fig. 5a), marked accumulation of inflammatory cells (especially neutrophils) within the lung microvasculature was observed at 2 hrs (Fig. 5b). Intermediate and late stages displayed various degrees of interstitial edema due to capillary leakage into the alveolar space, increased accumulation of macrophages, fibroblasts and collagen deposition (Fig. 5c).

## Discussions and Conclusion

Sepsis is a complex, multifactorial inflammatory dysregulation occurring when the host is unable to effectively control a bacterial infection. The disease eventually affects the lungs, heart, liver and kidneys, through multiple effects on endothelial, epithelial and immune cells. Despite progress in understanding its pathophysiology, sepsis is still a common disease with rising incidence and mortality. Many promising therapeutic approaches that were effective in animal studies failed to show similar benefit in human clinical trials[29]. This may be due to the fact that rodent models do not faithfully recapitulate the human disease, or to improper staging of the disease in animal models as compared with humans. Advances in understanding the sequence of events and their specific biomarkers during the progressive stages of sepsis would increase the ability to define appropriate therapeutic targets and to define human populations for successful therapeutic interventions. Our group has thoroughly investigated the changes in plasma proteins during the two-stage compensated response to sublethal inflammatory *E. coli *challenge[30]. The first-stage of events occurred within the first 4–6 hrs post-infusion and was characterized by massive production of vasoactive peptides and cytokines, closely followed by activation of coagulation, margination and activation of neutrophils, release of sympathomimetic effectors, and leading to a transient microvascular vasoconstriction and ischemia[17]. The second-stage events occur after 12 hrs post challenge and include post reperfusion oxidative stress, complement activation, and a second round of hemostatic activity[17]. However, the underlying sequence of changes in gene expression after *E. coli *challenge at tissue level is unknown.

A major complication in sepsis is the progressively impaired lung function, which often leads to acute lung injury. Our aim was to understand the dynamics of the specific changes in the transcriptional program of the lung during the two stage inflammatory response to *E. coli *sepsis in baboons. The three time points selected, 2, 8 and 24 hours, represent the first, the transitional and the second stage of the pathophysiological response to *E. coli *sepsis. Identification of molecular fingerprints of the lung during sepsis may facilitate the development of new approaches for early diagnosis, treatment or prevention.

The baboon genome is not known yet; however, since the similarity between the human and baboon genomes is higher than 94%[22,31], and previous publications have demonstrated that human DNA arrays can be effectively cross-hybridized with monkey cDNA[22,23,31,32], we used a human genome-specific oligonucleotide microarray to hybridize the baboon cDNA. We assume that the human probes that are hybridized by the homologous baboon cDNA provide valid information, while the non-hybridized probe-sets can reflect either the absence of the transcript or differences in the baboon sequence that prevent hybridization. However, the use of pathway analysis tools allows for the identification of networks of genes that are known to interact with each other. This procedure therefore provides confidence in the selected genes as well as clues to what other genes may be regulated but not identified as being significant by the microarray analysis.

We acknowledge two inherent limitations of our study. First, as we have extracted total RNA from whole lungs, our preparation contains transcripts originating from different cell types, therefore the spatial and cellular information as to the origin of the signal is lost. To partially overcome this limitation, we always have collected the tissue from the same region of the lung both for RNA extraction and for histological and ultrastructural analysis. The second limitation affects all gene array studies and derives from the fact that one gene can encode multiple spliced forms of transcripts, and further, the translated proteins undergo various post-translation modifications.

### Sequential induction of inflammatory and immune response genes

Sepsis involves an early strong activation of proinflammatory networks, triggered by the exposure of the host to LPS and other bacterial products. Most of the 35 genes that were induced by *E. coli *at 2 hrs belong to the inflammatory pathway and are regulated by, or interact with IL-1β and IL-6. Except for decreased THBD and FABP5, the remaining 33 genes are upregulated at this time-point.

It is clearly demonstrated both in humans and animal models that cytokine production/release is a sequential process. First, LPS induces proximal cytokines (TNF-α, IL-1β) via the activation of NF-kB pathway[33], and these two cytokines seem to mediate most of the patho-physiological disturbances characteristic to sepsis. TNF-α is very rapidly (within 60–90 min) induced when the inflammatory response is initiated. As our earliest time point was 2 hrs, no up-regulation of TNF-α mRNA was detected. However, we have detected elevated levels of TNF-α in plasma of *E. coli *challenged baboons, at all three time-points, as well as the mRNA of a variety of inflammatory mediators known to be regulated by TNF-α, including chemokines (CXCL2, CXCL10, CXCL11, CCL2, CCL4, CCL20), adhesive molecules (VCAM1, ICAM1, CEACAM8), cytokines and their receptors (IL-1β, IL-6, IL-1RN, IL-15RA, CSF3), TNFAIP2, TNFAIP3, TNIP1, the oxidative stress response gene HP, SOD2 and other inflammatory response genes S100A8, S100A9, NFKBIA, NFKBIE, GBP1, GBP2.

Next, proximal cytokines stimulate the production of distal cytokines IL-6 and IL-8 and/or anti-inflammatory mediators like IL-10[34].

IL-6 regulates the expression of complement components on endothelial cells and enhances cell function and production of pro-inflammatory mediators[35]. IL-8 is a potent activator and chemoattractant for neutrophils and it is thought to mediate neutrophilic infiltrates in the lung. In patients, high levels of IL-8 in broncho-alveolar lavage fluid were found to be predictive for the development of ARDS and were associated with high mortality[36]. In addition, IL-1β and TNF-α increase the expression of specific subsets of chemokines and adhesion molecules that appear to be involved in the pathogenesis of lung injury. Expression of E-selectin and ICAM1 on the pulmonary endothelium and alveolar epithelial cells enhances the adhesiveness of neutrophils to endothelial cells, facilitates transmigration into interstitial and distal airways and mediates the development of acute capillary leaks[35]. Chemokines are involved in immune and inflammatory responses where they act primarily as chemoattractants and activators of specific leukocyte subsets. CC chemokines positively enhance macrophage function, leading to enhanced generation of CXC chemokines that results in chemoattraction of neutrophils into lung interstitial and alveolar compartments[35]. We have detected increased gene expression of three CX and four CC chemokines. CXCL2 (MIP-2 α)-the highest expressed chemokine (10.8-fold at 2 hrs)- is produced by activated monocytes and neutrophils and expressed at sites of inflammation, and is also induced in vitro by LPS treatment of bronchoalveolar cells[37]. Two interferon γ-induced CX chemokines were detected. CXCL10 (IP-10; 4.3-fold at 2 hrs) is chemotactic for monocytes and T lymphocytes, and CXCL11 (IP-9; 4.4-fold at 2 hrs and 10.5-fold at 8 hrs) is an attractant for interleukin-activated T cells but not for unstimulated T cells, neutrophils or monocytes. CXCL11 induction by IFN-γ is enhanced by TNF-α in monocytes, fibroblasts and endothelial cells. Surprisingly, while several IFN-γ-induced genes were identified in this study, IFN-γ itself has not been detected. It is known from previous studies published by this lab that plasma levels of IFN-γ peak after 8 hrs post-challenge[38]. The lack of IFN-γ message could reflect a mismatch with the human probe, or a low content of the lung in t cells and antural killer cells, which are the main IFN γ-producing cells.

Among the CC chemokines, CCL20 (MIP 3α; over 8-fold increase at 2 hrs) attracts lymphocytes and, slightly, neutrophils, but not monocytes. It is induced by LPS, TNF-α, and IFN-γ and possesses antibacterial activity against *E. coli*. CCL3L1 is the only chemokine that was found increased at all three time-points. This is a chemotactic agent for lymphocytes and monocytes and a known inhibitor of HIV infection, but to date, no involvement in *E. coli *sepsis has been reported. CXCL5 (LIX or ENA-78), an attractant and activator of neutrophils, was the only downregulated chemokine. Interestingly, this chemokine was the highly expressed in a mouse model of LPS induced acute lung injury[6]. The divergent expression patterns for CXCL5 could reflect species-specific differences, or distinct responses to live bacteria vs. LPS challenge.

Two other inflammatory proteins, S100 calcium binding protein, S100A8 (calgranulin A) and S100A9 (calgranulin B), were among the highest expressed genes. S100A8 was increased by 12.0, 27.5 and 6.2-fold at 2 hrs, 8 hrs and 24 hrs, respectively, while S100A9 was up-regulated 7.7 and 17.5-fold at 2 and 8 hrs, respectively. S100A8 and S100A9 are proinflammatory neutrophil modulatory proteins. These proteins are found in the cytosol of neutrophils, monocytes and differentiated macrophages in inflamed tissues[39] as well as in the extracellular milieu during inflammatory conditions[40,41], where may play role in the recruitment and migration of neutrophils [42]. These two calgranulins play an important signaling role by inducing NF-kB activation and increased phosphorylation of p38 and p44/42 MAP kinases. Similar to another member of the calgranulin superfamily, S100A12, also referred to as ENRAGE, S100A8 and S100A9 interact with cellular RAGE on endothelium, mononuclear phagocytes, and lymphocytes triggering cellular activation, and generation of key proinflammatory mediators[43]. High levels of serum S100A8 and S100A9 were reported in chronic inflammation disorders, like rheumatoid arthritis, inflammatory bowel disease[44,45], bronchitis and tuberculosis[46,47]. The increased expression of calgranulins may reflect the large number of neutrophils and macrophages that accumulate in the lung, and may serve as additional molecular markers of early inflammatory response to infection.

GBP1 and GBP2 are two members of the guanylate-binding protein (GBP) family of GTPases that are strongly induced by IFN-γ˜
 MathType@MTEF@5@5@+=feaafiart1ev1aaatCvAUfKttLearuWrP9MDH5MBPbIqV92AaeXatLxBI9gBaebbnrfifHhDYfgasaacH8akY=wiFfYdH8Gipec8Eeeu0xXdbba9frFj0=OqFfea0dXdd9vqai=hGuQ8kuc9pgc9s8qqaq=dirpe0xb9q8qiLsFr0=vr0=vr0dc8meaabaqaciaacaGaaeqabaqabeGadaaakeaacuaHZoWzgaacaaaa@2E62@ and other inflammatory cytokines. GBP-1 mediates the inhibition of endothelial cell proliferation by inflammatory cytokines[48]. GBP-2 is expressed by macrophages and has mainly a vesicular location. Both proteins are considered as reliable markers of inflammatory phenotype for endothelium and macrophages, respectively[49,50]. GBP2 may also stimulate fibroblast proliferation and fibrosis[51].

Consistent with prior studies[52], we found that early proinflammatory response to sepsis in the lung is followed by the transition to an anti-inflammatory state. This is achieved by marked production of anti-inflammatory mediators (DISIP, IL1RN, IL15RA, TNFAIP3, NFKBIA and NFKBIE) and downregulation of proinflammatory genes during the second stage of the disease (TNFRSF8, CXCL5, C7, ITGB1BP1).

Our data demonstrating that IL-1 receptor antagonist is upregulated in the early stages support a tight regulation of pro- and anti-inflammatory responses in the lung. The resulting balance may determine the outcome of the disease, as recent reports demonstrate an increased ratio of IL-1 β to IL-1 receptor antagonist in patients with established ARDS[53,54]. During the predominantly anti-inflammatory stages, monocytes are deactivated, resulting in decreased HLA-DRB3 and consequent reduced antigen presentation, which is proposed as a major feature of sepsis-induced immunodepression[55]. Our data support a transcriptional down-regulation of a panel of genes required for MHC II-restricted antigen presentation that may occur in the course of septic shock[56]. In macrophages, DISIP (delta sleep peptide, immunoreactor) plays a role in the anti-inflammatory and immunosuppressive effects of IL-10 by inhibiting NFKB1 nuclear translocation. Two important anti-inflammatory mediators, IL-4 and IL-10 showed no change at mRNA level, but were detected by ELISA at 2 and 8 hrs respectively. This discrepancy may reflect sequence differences between human and baboon cDNA.

### Coagulation

Thrombomodulin (THBD) is one of few genes that are decreased at all 3 time points in this study. THBD is part of the anticoagulant pathway that mediates the activation of zymogen Protein C to activated Protein C (APC). APC is an enzyme that inhibits thrombin generation by degrading clotting factors Va and VIIIa, and regulates inflammation by inhibiting leukocyte activation, thereby reducing organ injury and microthrombus formation in sepsis. It is well documented that sepsis promotes procoagulant activity by strong upregulation of tissue factor (F3) paralleled by a decrease of tissue-associated THBD due to TNF-dependent shedding into the plasma[57], leading to the development of DIC associated with sepsis.

### Hypoxia, oxidative stress and apoptosis

Oxidative stress occurs when a homeostatic balance between the formation of reactive oxidizing oxygen species and their removal by endogenous antioxidant scavenging compounds is disrupted[58]. Sepsis may lead to oxidative stress either by excessive production of reactive oxygen species including superoxide, hydrogen peroxide and hydroxyl radicals and/or by inadequate antioxidative defense, including superoxide dismutase (SOD), catalase, vitamins C and E, and reduced glutathione[59]. One symptom of severe sepsis is the impaired ability of tissue to extract oxygen from the blood[60], leading to anaerobic metabolism.

Unexpectedly, we found that haptoglobin mRNA was highly increased in the lung of septic baboons. This plasma protein, normally produced by the liver, binds any excess free iron, preventing bacteria from using the iron to grow. In hemolysis, hemoglobin binds to haptoglobin to form a complex that will be absorbed, thus preventing its excretion into urine. Increased haptoglobin production by the lung is a novel finding, and may be an adaptive response to bacterial infection or may be partially due to the limited hemolysis caused by sepsis. Two stress-inducible proteins, SOD2 and TXNRD-1 are major scavengers of reactive oxygen species[61] but also have anti-apoptotic effects[62]. SOD2 is one of the key defense enzymes induced in host to destroy dangerous reactive oxygen species, such as superoxide radical (O^2-^) formed in the mitochondria as a byproduct of electron transport. It was reported that TNF-α increased the level of thioredoxin and SOD2 by a NF-kB dependent mechanism[63] and the overexpression of SOD2 prevents apoptosis induced by several oxidative stress inducers including TNF-α [64]. The dramatic upregulation of these genes at 2 hrs may reflect their role in counteracting the oxidative and proteolytic potential of the incoming neutrophils. Apoptosis is a highly regulated and evolutionarily conserved programmed process of cell death that is crucial for tissue remodeling and plays a very important role in normal cell regulation and in the pathology of sepsis[65]. Studies in experimental animals and critically ill patients have demonstrated that increased apoptosis of lymphoid organs and parenchymal tissues contribute to the immune suppression, anergy, and organ dysfunction. While lymphoid cells are undergoing accelerated apoptosis, spontaneous neutrophil apoptosis associated with sepsis or SIRS is delayed[66,67]. This decreased apoptosis is thought to actually enhance tissue injury in the lung by promoting a disbalanced tissue load of neutrophils and uncontrolled release of toxic metabolites injurious to endothelial cells' mitochondria and collagen deposition[66,68].

We have observed an increased expression of both pro- and anti-apoptotic genes at 2 hrs, coinciding with maximum expression peak of cytokines and other inflammatory mediators during the first stage. Conversely, antiapoptotic and cell survival promoting genes (HIPK3, MAPK8IP1, GTSE1, and DAP), were predominant during the second stage (24 hrs) where tissue regeneration-specific processes are prevalent.

### Extracellular matrix, tissue regeneration and functional recovery

Within the inflammatory process, a delicate balance exists between the potential for tissue destruction and mechanisms of protective immune defense and tissue repair. Repair of wounded tissue involves a well-orchestrated sequence of events involving infiltration of macrophages that are essential for removal of necrotic cellular debris. Further events include proliferation and migration of parenchymal and connective tissue cells, extracellular matrix deposition, tissue remodeling and angiogenesis. Electron microscopy analysis of lung samples illustrates that while the neutrophil migration is dominant during the first hours post *E. coli *challenge, macrophage and fibroblast accumulation and collagen deposition are the foremost ultrastructural events during the second stage of the response.

ECM remodeling and synthesis is controlled by de novo synthesis and by the balance between proteolytic enzymes and their inhibitors[69]. In this study, we observed downregulation of both MMP-2 and TIMP2 and 3 at 8 hrs and 24 hrs. MMP2 cleaves several collagens and plays a role in regulation of vascularization and in the inflammatory response. The decreased expressions of MMP2, its inhibitor TIMP2 and of two collagen (type IV α1 and α3) components of the capillary basement membrane suggest a possible downregulation of angiogenesis in sepsis. Besides its MMP inhibition effect, TIMP3 also inhibits TNF-α converting enzyme (TACE), which cleave TNF-α and regulates its shedding[70]. TIMP3 may have a protective role in the early stage by regulating TNF-dependent systemic inflammation, as TIMP3 knocked out mice are more susceptible to LPS-induced mortality[71]. The observed decreased expression of TIMP3 during the second stage may reflect the functional recovering post *E. coli *challenge.

SERPINA3 (α1-antichymotrypsin), a serpin that can inhibit potent proteases released by inflammatory cells, was strongly induced at 8 hrs post challenge. In addition to its role in matrix turnover, SERPINA3 may be also involved in the regulation of vessel's tone. SERPINA3 inhibits neutrophil cathepsin G and mast cell chymase, two proteases that convert angiotensin-1 to the active angiotensin-2, therefore SERPINA3 upregulation may be responsible for the reduced biological efficacy of the rennin-angiotensin system, and thus contribute to the cardiovascular collapse that occurs during sepsis. Our data highlight SERPINA3 as a potential therapeutic target to control the hemodynamic response of the vessel wall during sepsis. Natriuretic peptide precursor C (NPPC) is another vasoactive factor that we found to be upregulated by sepsis. NPPC possesses potent natriuretic, diuretic, and vasodilating activities and is implicated in body fluid homeostasis and blood pressure control. Normally it is expressed at low levels in endothelial cells, and its upregulation is a sign of endothelial dysfunction. The cardiovascular response to septic shock involves peripheral vasodilatation resulting in reduced systemic vascular resistance, hyporesponsiveness to vasopressors, and systemic hypotension. While natriuretic peptide precursors A and B were found in increased levels in septic patients and are markers of poor outcome, currently no data on the role of NPPC in sepsis have been published.

Many differentially expressed genes in the second stage of sepsis are involved in clearing tissue debris present at the wound site, or orchestrate aspects of tissue remodeling, cell proliferation and angiogenic processes associated with the wound response. This is particularly important, as poor wound healing and recurrent infection contribute to high mortality in septic patients[72]. Our data demonstrate that genes involved in tissue repair are turned on as early as 24 hrs after the onset of sepsis. While most of the genes are induced during the second stage (12–24 hrs), ACVR1B (activin receptor 1B), a TGF-β receptor with protein serine/threonine kinase activity was upregulated during the first stage. Its early expression may be important for mediating TGF activity during the second stage by participating in activation of SMAD transcriptional regulators. Activin upregulation in the lung after *E. coli *challenge is also a novel finding but its role is the pathophysiology of sepsis is not clear. In vitro studies support a role in the repair of the mesenchyme and possibly also of the epithelium, thus activin receptor upregulation could be beneficial for the repair of damaged tissues. However, prolonged and/or significantly increased expression of activin may lead to involved the development of fibrosis, thus a tight control of activin level is likely to be important for normal repair[73].

The increased expression of genes such as FAP, FGF23, FLT1 and TIE1 reflects the active tissue remodeling, regeneration and functional recovery at 24 hrs. Fibroblast activation protein α (FAP) is a membrane-bound gelatinase involved in the control of fibroblast growth or epithelial-mesenchymal interactions during tissue repair. Fibroblast growth factor 23(FGF23) possesses broad mitogenic and cell survival activities and is involved in cell growth and tissue repair.

TIE1 is a tyrosine kinase receptor specifically expressed in endothelial cells. Two angiopoietins, ANG1 and ANG4, activate TIE1, thus supporting a role of TIE1 in angiogenic remodeling[74]. Beside its angiopoietin-dependent function TIE1 has anti-apoptotic effects through downstream activation of PI-3 kinase and Akt pathways[75]. FLT1 (VEGFR-1) is a tyrosine kinase receptor for VEGF, VEGF B and PLGF that plays role in vascular development and regulation of vascular permeability. Recently, Yano et al reported increased circulating levels of soluble FLT1 in animal models and sepsis patients and suggested that circulating FLT1 may represent an endogenous compensatory anti-inflammatory mechanism[76]. The normal lung tissue abundantly expresses VEGF, the ligand for FLT1. VEGF is a HIF1-α-controlled endothelial mitogen that mediates increased vascular permeability, induces angiogenesis, vasculogenesis and endothelial cell growth, promotes cell migration, and inhibits apoptosis. Like HIF-α˜
 MathType@MTEF@5@5@+=feaafiart1ev1aaatCvAUfKttLearuWrP9MDH5MBPbIqV92AaeXatLxBI9gBaebbnrfifHhDYfgasaacH8akY=wiFfYdH8Gipec8Eeeu0xXdbba9frFj0=OqFfea0dXdd9vqai=hGuQ8kuc9pgc9s8qqaq=dirpe0xb9q8qiLsFr0=vr0=vr0dc8meaabaqaciaacaGaaeqabaqabeGadaaakeaacuaHXoqygaacaaaa@2E5A@, VEGF expression was increased at 2 hrs, followed by an unexpected decrease at 24 hr. Similar decreases in VEGF mRNA and protein in lung autopsy material from septic patients were reported[77]. However, the role of VEGF in sepsis-induced lung injury is still not clear. Interestingly, plasma levels of VEGF are increased during sepsis[78] and may contribute to morbidity and mortality, but it is most likely produced by other organs (heart, liver and kidneys)[76].

In summary, in this study we explored the changes in gene expression that occur in the lung at selected time-points following E. coli infusion in baboons. To our knowledge, this is first time that the expression pattern of inflammation-associated genes and the transcriptional regulation of sepsis-related genes were investigated in a temporal manner in a baboon model. The baboon model mimics human sepsis to a large extent, therefore our model offers an important resource in the exploration of the pathways and mechanisms involved in human sepsis. We found that the primary inflammatory response genes, innate immune response genes and some anti-apoptotic genes peaked at 2 hrs. Another group of inflammatory response genes and immune response genes that function as stimulation of monocytes, natural killer and T-cell migration, and modulation of adhesion molecule expression were increased and peaked at 8 hrs, while genes involved in wound healing and functional recovery were upregulated at 24 hrs. The transition and late expression genes may play important roles in survival. The global view of gene transcripts profile in the lungs of septic animals will significantly facilitate the understanding of the mechanism of sepsis-induced lung failure, and the pathophysiology of this disease. This may help exploring the development of new drugs and finding new targets for therapeutic intervention in severe sepsis.

## Methods

### Animal experimentation

The study protocol received prior approval by the Institutional Animal Care and Use Committee. Experiments were performed on 12 *Papio cyanocephalus *baboons. Nine animals were infused with live *E. coli *(Type B7 086a:k62; ATCC#33985, Rockville, MD) at the sublethal dose of 10^9 ^colony forming units (cfu)/kg and three were infused with saline (control), as described[79]. Three animals per time-point were sacrificed at 2, 8, and 24 hrs post-infusion. Three control animals were sacrificed after saline infusion. Lung tissue was collected from similar locations, snap-frozen immediately in liquid nitrogen, and stored at -80°C for mRNA and protein extraction. Adjacent lung tissue was immersed in fixative and further prepared for histology or electron microscopy.

### RNA isolation

Total RNA was isolated from frozen lung tissues collected from 12 baboons (control and 3 time-point groups; three animals per group) using TRIzol reagent (Invitrogen, CA) according to manufacturer's protocol. RNA concentration was measured with a NanoDrop^® ^ND1000 spectrophotometer (NanoDrop Technologies, Inc., Wilmington, DE), and its integrity and purity were verified by using an Agilent 2100 Bioanalyzer Capillary Gel Electrophoresis System (Agilent, Palo Alto, CA).

### Microarray slide

Microarrays were printed in-house using the Qiagen Operon v2.0 human oligonucleotide library (21,329 oligonucleotide probes) as previously described[80]. The 70-nucleotide oligos were derived from the functionally defined genes present in the UniGene database[81] and their length and sequence specificity were optimized to reduce the cross-hybridization problems encountered with cDNA-based microarrays. The list of genes present on the array is available[82]. All 11,000 human genes of known or suspected function were represented on these arrays. In addition, most undefined open reading frames were represented (approximately 10,000 additional genes). Oligonucleotides were spotted onto Corning^® ^UltraGAPS™ amino-silane-coated slides (Acton, MA, USA), rehydrated with water vapor, snap-dried at 90°C, and then covalently fixed to the surface of the glass using 300-mJ, 254-nm wavelength UV radiation. Unbound free amines on the glass surface were blocked for 15 minutes with moderate agitation in a 143 mM solution of succinic anhydride dissolved in 1-methyl-2-pyrolidinone, 20 mM sodium borate, pH 8.0. Slides were rinsed for 2 minutes in distilled water, immersed for 1 minute in 95% ethanol, and dried with a stream of nitrogen gas.

### Labeling and array hybridization

cDNA was synthesized from 10 μg of total RNA using CyScribe cDNA Post Labeling Kit (Amersham, Piscataway, NJ), labeled with Cy3 and purified using a Rapid PCR Purification System (Marligen Bioscience Inc, Ijamsville, MD) as previously reported[80]. Next, labeled cDNA was added to ChipHybe™ hybridization buffer (Ventana Medical Systems, Tucson, AR) containing Cot-1 DNA (0.5 mg/ml final concentration), yeast tRNA (0.2 mg/ml), and poly (dA)40–60 (0.4 mg/ml). Hybridization was performed on a Ventana Discovery system for 6 hr at 42°C. Microarrays were washed to a final stringency of 0.1× SSC, and then scanned using an Agilent DNA Microarray Scanner (Agilent Technologies, Santa Clara, CA). Fluorescent intensity was measured by Imagene™ software (BioDiscovery, Marina del Rey, CA).

### Array analysis

Data normalization and robust regression was conducted to correct for technical variation among individual microarray hybridizations. This was conducted using standard statistical analysis methods in Matlab software, as described[83,84]. Genes differentially expressed between groups of samples were selected using associative analysis, as previously described[83]. For selection of genes that responded to *E. coli *challenge, each time point was compared against controls (time 0). The fold ratio between each time point (2, 8, and 24 hrs) versus control (time 0) was generated using the average of normalized gene expression values of three biological replica of each group. The genes have a ratio either > 2 (up-regulated) or <0.5 (down-regulated) were selected as candidates for further studies. Wards's hierarchical clustering was done using the Spotfire DecisionSite for Functional Genomics 8.1 package (Spotfire, Inc., Somerville, MA, USA). Similarity measure was the Euclidean distance, the clustering method was Unweighted Pair Group Method with Arithmetic Mean, and input rank was the ordering function.

### Real-Time RT-PCR

Real time quantitative PCR was conducted on ABI Prism 7000 Sequence Detection System (Applied Biosystems, Foster City, CA). iTaqTM SYBR^® ^Green Supermix (Bio-Rad, CA) was used for detection and quantitation according to manufacturer protocol. For a typical reaction, 12.5 μl of iTaq SYBR Green 2× supermix, 10 μM of each primer and equal or less than 200 ng of cDNA template were mixed together and the final volume was adjusted to 25 μl. Expression levels of β-actin were tested along with each sample at each time point for normalization during the relative quantification. Each gene was tested using two different cDNAs and the analysis was run in triplicate. The default PCR conditions were as follows: (i) initiation: 2 min at 50°C; (ii) hot start of the enzyme: 10 min at 95°C; (iii) amplification: denaturation at 95°C for 15 seconds, followed by annealing and extension at 60°C for 1 min; 40 cycles. The specificity of gene PCR product was evaluated by melt dissociation curve.

### Pathway Analysis

Biologically relevant networks were drawn clusters of differentially expressed genes. Pathways were generated through the use of IPA software (Ingenuity Systems, Mountain View, CA). The IPA uses the Ingenuity Pathways Knowledge Base (IPKB), which is a curated database of biological networks, consisting of millions of individually-modeled peer-reviewed pathway relationships. This enabled the identification of sepsis-induced biologically relevant networks by visual inspection.

Expression data sets containing gene identifiers (Entrez Gene ID) and their corresponding expression values as fold changes were uploaded in the IPA. Each gene identifier was mapped to its corresponding gene object in the Ingenuity Pathways Knowledge Base[85]. These "focus genes" were then used as starting point for generating biological networks. The application program queries the database for interactions between focus genes and all other gene objects stored in the knowledge base, and generates a set of networks. Biological functions were assigned to each gene network by using the findings extracted from the published scientific literature and stored in the Ingenuity Pathways Knowledge Base[85] and are ranked according to the significance of that biological function to the network. The program then computes a score for each network according to the fit of the network to the set of focus genes. The score is derived from a p-value and indicates the likelihood of the focus genes in a network being found together because of random chance. A score of greater than 2 indicates that there is a less than 1 in 100 chance that the focus genes were assembled randomly into a network due to random chance. In the current study, a score of 6 (less than 1 in 1 million chances of random grouping) or higher was used to select highly significant biological networks regulated by *E. coli *sepsis. Gene symbols were colored according to the temporal behavior of their expression (red indicating up- and green down-regulated expression).

### Multiplex ELISA for inflammatory cytokines

Equal amounts of lung tissues were homogenized on ice with 1% Triton X-100, 60 mM octyl-b-D glucopirannoside (OGP) in 50 mM Tris-HCl, 150 mM NaCl, pH 8.5, supplemented with protease inhibitors (1.5 μM pepstatin A, 10.5 μM leupeptin, 0.25 mM pefabloc SC, 20.9 μM calpain inhibitor I, 20 μM calpain inhibitor II, 1.5 μM aprotinin, 1 mM benzamidine, 1 mM EDTA, 1 mM sodium ortho-vanadate, and 1 mM 1,10-phenanthroline). The extracts were centrifuged at 14,500 × g for 15 min, and the supernatants, representing the lung tissue lysates, were collected and stored at -80°C. Total protein was determined using a BCA protein kit (Pierce, Rockford, IL). Ten inflammatory cytokines (IL-1β, IL-2, IL-4, IL-5, IL-6, IL-8, IL-10, IFN-γ, TNF-α, and GM-CSF) were measured using a kit for human cytokines from BioSource International in conjunction with a Luminex multiplex system (Luminex Corporation)[86] and normalized to mg of total protein for tissue extracts or to ml of plasma.

### Morphological analysis

For electron microscopy, tissue samples were fixed with glutaraldehyde and osmium tetraoxide, embedded in epoxy resin and further prepared and examined as previously described[87].

## Competing interests

The author(s) declare that they have no competing interests.

## Authors' contributions

HZ performed mRNA extraction, real time PCR validation, data processing and writing the first draft of the manuscript; YT did the bioinformatics analysis; LI performed multiplex ELISA assays; MC coordinated the microarray and bioinformatics analysis; CL coordinated the microscopy and biochemical analysis and substantially edited the manuscript, FBT contributed with the animal model of sepsis and data interpretation. FL conceived the study, participated in its designed and coordination and wrote the final version of the manuscript. All authors read and approved the final version of the manuscript.

## Supplementary Material

Additional file 2 Table_A2. The table lists the genes of the IPA networks at the time point 2 hrs. Click here for file

Additional file 3 Table_A3. The table lists the genes of the IPA networks at the time point 8 hrs. Click here for file

Additional file 4 Table_A4. The table lists the genes of the IPA networks at the time point 24 hrs. Click here for file

Additional file 1 Table_A1. The table lists the genes presented in the cluster analysis. Click here for file

## Figures and Tables

**Table 1:** Genes up-regulated by sepsis in the lung of the baboons at 2, 8 and 24 hr post *E. coli *challenge

**Gene Accession no.**	**Gene Product**	**Fold change compared to control**		
		
		**2 h**	**8 h**	**24 h**
*Cytokine or chemokine*				
NM_002089	chemokine (C-X-C motif) ligand 2 (CXCL2)	10.8		
NM_004591	chemokine (C-C motif) ligand 20 (CCL20)	8.3		
NM_000576	interleukin 1, beta (IL1B)	4.5		
NM_005409	chemokine (C-X-C motif) ligand 11 (CXCL11)	4.4	10.5	
NM_001565	chemokine (C-X-C motif) ligand 10 (CXCL10)	4.3		
NM_002982	chemokine (C-C motif) ligand 2 (CCL2)	3.5		
NM_002198	interferon regulatory factor 1 (IRF1)	3.0	3.8	
NM_000600	interleukin 6 (interferon, beta 2) (IL6)	2.2		
NM_002189	interleukin 15 receptor, alpha (IL15RA)	2.2		
NM_002984	chemokine (C-C motif) ligand 4 (CCL4)	2.2		
NM_000759	colony stimulating factor 3 (granulocyte) (CSF3)	4.4		
D90145	chemokine (C-C motif) ligand 3-like 1(CCL3L1)	2.6	3.0	2.0
				
*Cytokine suppression or regulation*				
X52015	interleukin 1 receptor antagonist (IL1RN)	2.8		
				
*Adhesion molecules*				
NM_001078	vascular cell adhesion molecule 1 (VCAM1)	6.9	4.7	
NM_000201	intercellular adhesion molecule 1 (CD54), human rhinovirus receptor (ICAM1)	3.5		
NM_001816	carcinoembryonic antigen-related cell adhesion molecule 8 (CEACAM8)	2.0		
NM_021219	junctional adhesion molecule 2 (JAM2)		2.0	2.3
NM_003370	vasodilator-stimulated phosphoprotein (VASP)		2.3	2.1
AF139768	C-type (calcium dependent, carbohydrate-recognition domain) lectin, superfamily member 5		2.01	
NM_002999	syndecan 4 (amphiglycan, ryudocan) (SDC4)	6.0		
				
*Other inflammation-associated genes*				
NM_004120	guanylate binding protein 2, interferon-inducible (GBP2)	10.2	16.3	2.3
NM_002053	guanylate binding protein 1, interferon-inducible, 67 kDa (GBP1)	3.8	4.5	
NM_006291	tumor necrosis factor, alpha-induced protein 2 (TNFAIP2)	3.4		
NM_006058	TNFAIP3 interacting protein 1 (TNIP1)	2.9		
NM_006290	tumor necrosis factor, alpha-induced protein 3 (TNFAIP3)	2.5		
NM_003821	receptor-interacting serine-threonine kinase 2 (RIPK2)	2.1		
NM_002964	S100 calcium binding protein A8 (calgranulin A) (S100A8)	12.0	27.5	6.2
NM_002965	S100 calcium binding protein A9 (calgranulin B) (S100A9)	7.7	17.5	
NM_007053	CD160 antigen (CD160)		2.2	2.0
NM_001276	chitinase 3-like 1 (cartilage glycoprotein-39) (CHI3L1)		3.2	2.5
NM_004271	lymphocyte antigen 86 (LY86) (MD-1)			2.3
NM_001085	serine (or cysteine) proteinase inhibitor, clade A (alpha-1 antiproteinase, antitrypsin), member 3 (SERPINA3)	5.6	6.8	
NM_002818	proteasome (prosome, macropain) activator subunit 2 (PA28 beta) (PSME2)			3.1
NM_004159	proteasome (prosome, macropain) subunit, beta type, 8 (large multifunctional protease 7) (PSMB8)			3.2
				
*Hypoxia and/or oxidative stress*				
NM_001530	hypoxia-inducible factor 1, alpha subunit (basic helix-loop-helix transcription factor) (HIF1A)	2.9	2.5	
NM_003330	thioredoxin reductase 1 (TXNRD1)	2.1		
NM_000636	superoxide dismutase 2, mitochondrial (SOD2)	12.5		5.0
AK055872	Haptoglobin (HP)	33.3	24.4	
				
*Apoptosis*				
AF070674	baculoviral IAP repeat-containing 3 (BIRC3)	2.9		
NM_015675	growth arrest and DNA-damage-inducible, beta (GADD45B)	3.0		
NM_002727	proteoglycan 1, secretory granule (PRG1)	2.3	2.4	
NM_052889	CARD only protein (COP)	9.6	11.8	
NM_003311	pleckstrin homology-like domain, family A, member 2 (PHLDA2)		3.1	
NM_005734	homeodomain interacting protein kinase 3 (HIPK3)			2.0
NM_016426	G-2 and S-phase expressed 1 (GTSE1)			2.0
NM_005456	mitogen-activated protein kinase 8 interacting protein 1 (MAPK8IP1)			2.3
				
*Heat shock protein*				
NM_014365	heat shock 27 kDa protein 8 (HSPB8)	3.0	2.7	
				
*Cell proliferation tumor suppressor wound healing process*				
NM_003311	pleckstrin homology-like domain, family A, member 2 (PHLDA2)			2.8
NM_021077	neuromedin B (NMB)			2.0
NM_006069	murine retrovirus integration site 1 homolog (MRVI1)			2.7
NM_001260	cyclin-dependent kinase 8 (CDK8)			2.7
NM_003073	SWI/SNF related, matrix associated, actin dependent regulator of chromatin, subfamily b, member 1 (SMARCB1)			2.4
AK055327	fibroblast activation protein, alpha (FAP)			4.3
NM_005424	tyrosine kinase with immunoglobulin and epidermal growth factor homology domains (TIE)			2.9
NM_002408	mannosyl (alpha-1,6-)-glycoprotein beta-1,2-N-acetylglucosaminyltransferase (MGAT2)			2.9
NM_020638	fibroblast growth factor 23 (FGF23)			2.3
NM_006603	stromal antigen 2 (STAG2)			2.3
NM_005622	SA hypertension-associated homolog (rat) (SAH)			2.3
AB011537	slit homolog 1 (Drosophila) (SLIT1)			2.2
J05158	carboxypeptidase N, polypeptide 2, 83 kD (CPN2)			2.1
AF339822	fms-related tyrosine kinase 1 (vascular endothelial growth factor/vascular permeability factor receptor) (FLT1)		2.2	3.2
AK056352	PR domain containing 5 (PRDM5)		2.0	2.5
AL117643	activin A receptor, type IB (ACVR1B)	2.4		
				
*Coagulation*				
NM_001993	coagulation factor III (thromboplastin, tissue factor) (F3)	2.5		
NM_014059	response gene to complement 32 (RGC32)		3.7	
				
*Blood pressure*				
NM_024409	natriuretic peptide precursor C (NPPC)			2.8
				
*Energy generation*				
AF327403	solute carrier family 25, member 28 (SLC25A28)	2.1		
NM_000761	cytochrome P450, family 1, subfamily A, polypeptide 2 (CYP1A2)		3.5	3.4
AL359600	ATP-binding cassette, sub-family C (CFTR/MRP), member 5 (ABCC5)	2.3		
AF052090	nicotinamide nucleotide transhydrogenase (NNT)			2.7
NM_004297	guanine nucleotide binding protein (G protein), alpha 14 (GNA14)			2.1
				
*Protein synthesis*				
AL161952	glutamate-ammonia ligase (glutamine synthase) (GLUL)	2.1		
NM_004184	tryptophanyl-tRNA synthetase (WARS)	2.1	8.0	3.4
NM_002954	ribosomal protein S27a (RPS27A)		2.1	
NM_013417	isoleucine-tRNA synthetase (IARS)		2.0	
				
*Cation transporter*				
NM_017964	solute carrier family 30 (zinc transporter), member 6 (SLC30A6)			2.0
NM_022154	solute carrier family 39 (zinc transporter), member 8 (SLC39A8)	4.4	3.6	
D31887	solute carrier family 39 (zinc transporter), member 14 (SLC39A14)		2.2	
				
*Transcriptional regulation*				
NM_020529	nuclear factor of kappa light polypeptide gene enhancer in B-cells inhibitor, alpha (NFKBIA)	6.2		
AK026497	YY1 transcription factor (YY1)	2.6		
NM_004556	nuclear factor of kappa light polypeptide gene enhancer in B-cells inhibitor, epsilon (NFKBIE)	2.4		
NM_013448	bromodomain adjacent to zinc finger domain, 1A (BAZ1A)	2.0		
NM_015859	general transcription factor IIA, 1, 19/37 kDa (GTF2A1)		2.1	
NM_004697	PRP4 pre-mRNA processing factor 4 homolog (yeast) (PRPF4)			2.7
NM_005523	homeo box A11 (HOXA11)			2.1
NM_006540	nuclear receptor coactivator 2 (NCOA2)			3.5
NM_012345	nuclear fragile × mental retardation protein interacting protein 1 (NUFIP1)			3.4
NM_004719	splicing factor, arginine/serine-rich 2, interacting protein (SFRS2IP)			3.3
NM_004514	forkhead box K2 (FOXK2)			3.1
BG163465	I-mfa domain-containing protein (HIC)			2.0
AK025842	nuclear receptor subfamily 2, group F, member 2 (NR2F2)			2.0
				
*Cell motility, phagocytosis, trafficking*				
NM_002356	myristoylated alanine-rich protein kinase C substrate (MARCKS)	3.0		
NM_006086	tubulin, beta, 4 (TUBB4)		2.5	3.5
AK023457	RAP1A, member of RAS oncogene family (RAP1A)			2.7
				
*Lipid*				
AF323540	apolipoprotein L, 1 (APOL1)	3.7	12.4	
NM_005564	lipocalin 2 (oncogene 24p3) (LCN2)	8.6	10.7	
NM_004190	lipase, gastric (LIPF)			2.1
				
*Protein glycosylation*				
NM_004737	like-glycosyltransferase (LARGE)		2.3	
AB067505	UDP-N-acetyl-alpha-D-galactosamine:polypeptide N-acetylgalactosaminyltransferase 13 (GalNAc-T13)			2.0
NM_006042	heparan sulfate (glucosamine) 3-O-sulfotransferase 3A1 (HS3ST3A1)			2.1
AF155510	Heparanase (HPSE)			2.1
NM_005424	yrosine kinase with immunoglobulin and epidermal growth factor homology domains (TIE)			2.9
NM_002408	mannosyl (alpha-1,6-)-glycoprotein beta-1,2-N-acetylglucosaminyltransferase (MGAT2)			2.9
NM_006577	UDP-GlcNAc:betaGal beta-1,3-N-acetylglucosaminyltransferase 1 (B3GNT1)			3.3
				
*Signal transduction*				
NM_002401	mitogen-activated protein kinase kinase kinase 3 (MAP3K3)			3.0
NM_001721	BMX non-receptor tyrosine kinase (BMX)			2.6
NM_004297	guanine nucleotide binding protein (G protein), alpha 14 (GNA14)			2.1
NM_002075	guanine nucleotide binding protein (G protein), beta polypeptide 3 (GNB3)	2.6	2.2	
BC011671	guanine nucleotide binding protein (G protein), beta 5 (GNB5)		2.7	
NM_004125	guanine nucleotide binding protein (G protein), gamma 10 (GNG10)		2.2	2.5
NM_005279	G protein-coupled receptor 1 (GPR1)			3.3

**Table 2:** Genes downregulated by sepsis in the lung of the baboons at 2, 8 and 24 hrs post *E. coli *challenge

**Gene Accession no.**	**Gene Product**	**Fold change compared to control**		
		
		2 h	6–8 h	24 h
*Cytokine, chemokine, inflammation associated gene*				
NM_001243	tumor necrosis factor receptor superfamily, member 8 (TNFRSF8)		0.23	0.18
NM_000014	alpha-2-macroglobulin (A2M)		0.48	
NM_002994	chemokine (C-X-C motif) ligand 5 (CXCL5)		0.50	
NM_004763	integrin beta 1 binding protein 1 (ITGB1BP1)		0.50	
				
*Immune response*				
NM_000587	complement component 7 (C7)			0.32
NM_002125	major histocompatibility complex, class II, DR beta 3 (HLA-DRB3)			0.33
				
*Scaffolding protein*				
NM_001753	Caveolin 1 protein (CAV1)	0.49		
				
*Blood coagulation*				
NM_000361	Thrombomodulin (THBD)	0.39	0.17	0.25
NM_000611	CD59 antigen p18–20 (antigen identified by monoclonal antibodies 16.3A5, EJ16, EJ30, EL32 and G344) (CD59)			0.42
NM_001150	alanyl (membrane) aminopeptidase (aminopeptidase N, aminopeptidase M, microsomal aminopeptidase) (ANPEP)			0.43
				
*Collagen catabolism, extra cellular matrix*				
NM_004530	matrix metalloproteinase 2 (gelatinase A, 72 kDa gelatinase, 72 kDa type IV collagenase) (MMP2)		0.36	0.36
AL110197	tissue inhibitor of metalloproteinase 2 (TIMP2)		0.46	0.45
NM_001848	collagen, type VI, alpha 1 (COL6A1)		0.48	
NM_004369	collagen, type VI, alpha 3 (COL6A3)			0.30
NM_000362	tissue inhibitor of metalloproteinase 3 (Sorsby fundus dystrophy, pseudoinflammatory) (TIMP3)			0.32
				
*Cell adhesion, cell migration*				
NM_001901	connective tissue growth factor (CTGF)	0.37		
AY027862	upregulated in colorectal cancer gene 1 (UCC1)		0.40	
NM_001769	CD9 antigen (p24) (CD9)		0.30	
NM_002026	fibronectin 1 (FN1)		0.37	
AL050364	plakophilin 4 (PKP4)		0.44	0.43
NM_001305	claudin 4 (CLDN4)		0.46	
AF022375	vascular endothelial growth factor (VEGF)			0.40
NM_013994	discoidin domain receptor family, member 1 (DDR1)			0.44
NM_014000	Vinculin (VCL)			0.50
				
*Anti-inflammation*				
AB025432	delta sleep inducing peptide, immunoreactor (DSIPI)	0.41		0.39
				
*Cell growth, proliferation, differentiation, cell cycle*				
NM_001946	dual specificity phosphatase 6 (DUSP6)	0.45		
NM_002165	inhibitor of DNA binding 1, dominant negative helix-loop-helix protein (ID1)	0.34		
NM_006286	transcription factor Dp-2 (E2F dimerization partner 2) (TFDP2)		0.37	
NM_002276	keratin 19 (KRT19)		0.35	
NM_001424	epithelial membrane protein 2 (EMP2)		0.42	0.44
NM_001431	erythrocyte membrane protein band 4.1-like 2 (EPB41L2)		0.42	
NM_005610	retinoblastoma binding protein 4 (RBBP4)		0.45	0.45
NM_006407	cytoskeleton related vitamin A responsive protein (JWA)		0.45	
AL122055	cyclin-dependent kinase (CDC2-like) 11 (CDK11)		0.45	
NM_016250	NDRG family member 2 (NDRG2)		0.46	
AF368463	carboxypeptidase M (CPM)		0.47	0.47
NM_005708	glypican 6 (GPC6)		0.49	
NM_006835	cyclin I (CCNI)			0.20
AB023182	serine/threonine kinase 38 like (STK38L)		0.32	0.11
AF218029	H3 histone, family 3B (H3.3B) (H3F3B)			0.22
NM_001431	erythrocyte membrane protein band 4.1-like 2 (EPB41L2)			0.25
NM_006185	nuclear mitotic apparatus protein 1 (NUMA1)			0.36
NM_001553	insulin-like growth factor binding protein 7 (IGFBP7)			0.37
NM_001980	Epimorphin (EPIM)			0.38
AK055976	thymosin, beta 4, X-linked (TMSB4X)			0.40
NM_005778	RNA binding motif protein 5 (RBM5)			0.41
NM_014399	transmembrane 4 superfamily member 13 (TM4SF13)			0.41
NM_006791	mortality factor 4 like 1 (MORF4L1)			0.47
				
*Transcriptional regulation*				
NM_001271	chromodomain helicase DNA binding protein 2 (CHD2)		0.38	
NM_003924	paired-like homeobox 2b (PHOX2B)		0.41	0.43
AJ420430	general transcription factor IIIC, polypeptide 4, 90 kDa (GTF3C4)		0.41	
NM_002938	ring finger protein 4 (RNF4)		0.44	0.28
D49835	ras responsive element binding protein 1 (RREB1)		0.49	
NM_002624	prefoldin 5 (PFDN5)			0.32
NM_003418	zinc finger protein 9 (a cellular retroviral nucleic acid binding protein) (ZNF9)			0.34
NM_031243	heterogeneous nuclear ribonucleoprotein A2/B1 (HNRPA2B1)			0.36
NM_003151	signal transducer and activator of transcription 4 (STAT4)			0.38
AB058726	PHD finger protein 6 (PHF6)		0.34	0.39
NM_002228	v-jun sarcoma virus 17 oncogene homolog (avian) (JUN)			0.40
NM_003205	transcription factor 12 (HTF4, helix-loop-helix transcription factors 4) (TCF12)			0.43
NM_031314	heterogeneous nuclear ribonucleoprotein C (C1/C2) (HNRPC)			0.43
NM_001300	core promoter element binding protein (COPEB)			0.45
AL133574	TEA domain family member 1 (SV40 transcriptional enhancer factor) (TEAD1)			0.45
AK027071	transforming growth factor beta-stimulated protein TSC-22 (TSC22)			0.46
AJ420430	general transcription factor IIIC, polypeptide 4, 90 kDa (GTF3C4)			0.50
				
*Apoptosis, stress response*				
NM_000581	glutathione peroxidase 1 (GPX1)			0.30
NM_000041	apolipoprotein E (APOE)			0.36
NM_004394	death-associated protein (DAP)			0.40
NM_000851	glutathione S-transferase M5 (GSTM5)			0.45
NM_021003	protein phosphatase 1A (formerly 2C), magnesium-dependent, alpha isoform (PPM1A)			0.48
				
*Energy generation or protein synthesis*				
NM_020041	solute carrier family 2 (facilitated glucose transporter), member 9 (SLC2A9)		0.42	
AL136693	cytochrome b reductase 1 (CYBRD1)		0.42	
NM_002164	indoleamine-pyrrole 2,3 dioxygenase (INDO)		0.44	0.29
AF113701	ribosomal protein L22 (RPL22)		0.49	
NM_021194	solute carrier family 30 (zinc transporter), member 1 (SLC30A1)		0.49	
NM_000971	ribosomal protein L7 (RPL7)			0.22
NM_015994	ATPase, H+ transporting, lysosomal 34 kDa, V1 subunit D (ATP6V1D)			0.31
NM_001010	ribosomal protein S6 (RPS6)			0.32
NM_001867	cytochrome c oxidase subunit VIIc (COX7C)			0.35
NM_020041	solute carrier family 2 (facilitated glucose transporter), member 9 (SLC2A9)			0.38
NM_001967	eukaryotic translation initiation factor 4A, isoform 2 (EIF4A2)			0.42
NM_001961	eukaryotic translation elongation factor 2 (EEF2)			0.43
NM_001418	eukaryotic translation initiation factor 4 gamma, 2 (EIF4G2)			0.43
NM_003753	eukaryotic translation initiation factor 3, subunit 7 zeta, 66/67 kDa (EIF3S7)			0.43
NM_001460	flavin containing monooxygenase 2 (FMO2)			0.45
AK057575	solute carrier family 25 (mitochondrial carrier* phosphate carrier), member 3 (SLC25A3)			0.46
AK057299	dihydrolipoamide S-acetyltransferase (E2 component of pyruvate dehydrogenase complex) (DLAT)			0.48
NM_016091	eukaryotic translation initiation factor 3, subunit 6 interacting protein (EIF3S6IP)			0.50
				
*Cardiovascular calcifications*				
NM_000900	matrix Gla protein (MGP)		0.25	0.19
				
*Protein transport*				
NM_001282	adaptor-related protein complex 2, beta 1 subunit (AP2B1)			0.38
NM_004914	RAB36, member RAS oncogene family (RAB36)			0.40
NM_002865	RAB2, member RAS oncogene family (RAB2)			0.34
AK057157	RAB6A, member RAS oncogene family (RAB6A)			0.25
AK000757	sortilin 1 (SORT1)			0.47

**Table 3:** Oligonucleotide sequences of the primers used for QRT-PCR

*GenBank ID*	*Gene*	*Primer pair sequences*
AF070674	BIRC3	F 5'-GTG ATG GTG GAC TCA GGT GTT G-3' R 5'-TCC TGT CCT TTA ATT CTT ATC AAG TAC TCA-3'
NM052889	COP1	F 5'-CAG GCA GTG CGA GGA CAA-3' R 5'-AGC AAA GCT TGA TTC TGC CTT CT-3'
NM001530	HIF1A	F 5'-CCA AAT CCA GAG TCA CTG GAA CTT-3' R 5'-CAT CGG AAG GAC TAG GTG TCT GA-3'
NM003821	RIPK2	F 5'-ACG TCT GCA GCC TGG TAT AGC-3' R 5'-GGC AGG CTT CTG TCA TTT GG-3'
NM002964	S100A8	F 5'-CAA GTC CGT GGG CAT CAT G-3' R 5'-AGT ACT TGT GGT AGA CGT CGA TGA TAG-3'
NM002999	SDC4	F 5'-GGC AAG AAA CCC ATC TAC AAG AA-3' R 5'-GCC AGT GCC CAC AAG CA-3'
NM006290	TNFAIP3	F 5'-CTG CCC AGG AAT GCT ACA GAT AC-3' R 5'-AGG GTC ACC AAG GGT ACA AAA TG-3'
NM001753	CAV1	F 5'-ACA CCG TCT GTG ACC CAC TCT-3' R 5'-CTG CAA GTT GAT GCG GAC AT-3'
NM000361	THBD	F 5'-CCA GCT CTA CTG GTC TTG TGG AA-3' R 5'-GAT TAA GGC TAG GCC CTA ATT GG-3'
BC013835	β-actin	F 5'-AAG CCA CCC CAC TTC TCT CTA A-3' R 5'-AAT GCT ATC ACC TCC CCT GTG T-3'

Abbreviations: F, forward; R, reverse.

**Table 4:** Gene expression validation using QRT-PCR

***Gene***	***Microarrays***				***QRT-PCR***				***Correlation coefficient***
	***0***	***2 hr***	***8 hr***	***24 hr***	***0***	***2 hr***	***8 hr***	***24 h***	
TNFAIP3	9.64	23.66	17.60	10.87	1	8.77	4.63	151	0.995
BIRC3	7.77	22.24	14.76	7.23	1	3.79	2.52	1.27	0.993
RIPK2	6.73	14.17	10.43	8.34	1	9.48	5.65	1.24	0.978
SDC4	4.27	25.44	6.77	7.91	1	4.23	1.59	0.84	0.967
THBD	28.10	11.15	4.97	7.09	1	0.3	0.33	0.21	0.958
CAV1	13.31	6.65	7.15	3.44	1	0.64	0.51	0.61	0.843
COP	1.38	13.28	16.27	2.94	1	1.19	3.85	0.18	0.782
S100A8	9.29	111.85	255.40	57.64	1	9.41	8.77	4.96	0.779
HIF1A	6.17	17.79	15.62	7.77	1	2.13	6.66	1.13	0.602
