# Supplementary material for: Temporal dynamics of gene expression in the lung in a baboon model of E. coli sepsis
Source: BMC Genomics. 2007 Feb 26;8:58. doi: 10.1186/1471-2164-8-58 (PMC1819384; doi:10.1186/1471-2164-8-58)
Supplement: Additional file 2 — Table_A2. The table lists the genes of the IPA networks at the time point 2 hrs. [file 1471-2164-8-58-S2.doc]

***Table A2:* Genes included in the IPA networks at 2 hrs**

| ***Name*** | ***Description*** | ***Genbank*** | ***Ntwk*** | ***Location*** | *Family* |
| --- | --- | --- | --- | --- | --- |
| ABCC5 | ATP-binding cassette, sub-family C (CFTR/MRP), member 5 | AL359600 |  | Plasma Membrane | transporter |
| ACVR1B | activin A receptor, type IB | AL117643 | 2 | Plasma Membrane | kinase |
| ADCY1 | adenylate cyclase 1 (brain) | -- | 4 | Plasma Membrane | enzyme |
| ADCY2 | adenylate cyclase 2 (brain) | -- | 4 | Plasma Membrane | enzyme |
| AFM | afamin | NM_001133 | 2 | Extracellular Space | transporter |
| AKT1 | v-akt murine thymoma viral oncogene homolog 1 | -- | 4 | Cytoplasm | kinase |
| APOL1 | apolipoprotein L, 1 | AF323540 |  | Extracellular Space | transporter |
| APP | amyloid beta (A4) precursor protein (peptidase nexin-II, Alzheimer disease) | -- | 2 | Plasma Membrane | other |
| ARG1 | arginase, liver | -- | 3 | Cytoplasm | enzyme |
| ASS | argininosuccinate synthetase | -- | 3 | Cytoplasm | enzyme |
| BAZ1A | bromodomain adjacent to zinc finger domain, 1A | NM_013448 | 9 | Nucleus | other |
| BIRC3 | -- | AF070674 | 1 | Cytoplasm | other |
| BNIP3 | BCL2/adenovirus E1B 19kDa interacting protein 3 | -- | 3 | Cytoplasm | other |
| CA6 | carbonic anhydrase VI | NM_001215 | 7 | Extracellular Space | enzyme |
| CACNA1B | calcium channel, voltage-dependent, L type, alpha 1B subunit | -- | 4 | Plasma Membrane | ion channel |
| CAV1 | caveolin 1, caveolae protein, 22kDa | NM_001753 | 4 | Plasma Membrane | other |
| CBX8 | chromobox homolog 8 (Pc class homolog, Drosophila) | -- | 5 | Nucleus | other |
| CCL2 | chemokine (C-C motif) ligand 2 | NM_002982 | 1 | Extracellular Space | cytokine |
| CCL4 | chemokine (C-C motif) ligand 4 | NM_002984 | 1, 2 | Extracellular Space | cytokine |
| CCL6 | -- | -- | 2 | Extracellular Space | cytokine |
| CCL20 | chemokine (C-C motif) ligand 20 | NM_004591 | 1 | Extracellular Space | cytokine |
| CCL3L1 | chemokine (C-C motif) ligand 3-like 1 | D90145 | 4 | Extracellular Space | cytokine |
| CCR3 | chemokine (C-C motif) receptor 3 | -- | 4 | Plasma Membrane | G-protein coupled receptor |
| CCR5 | chemokine (C-C motif) receptor 5 | -- | 4 | Plasma Membrane | G-protein coupled receptor |
| CD24 | CD24 molecule | -- | 2 | Plasma Membrane | other |
| CEACAM8 | carcinoembryonic antigen-related cell adhesion molecule 8 | NM_001816 | 1 | Plasma Membrane | other |
| CEBPD | CCAAT/enhancer binding protein (C/EBP), delta | NM_005195 | 1 | Nucleus | other |
| CHRAC1 | chromatin accessibility complex 1 | -- | 9 | Nucleus | other |
| CLU | clusterin | -- | 2 | Extracellular Space | other |
| COP (LOC114769) | caspase-1 dominant-negative inhibitor pseudo-ICE | NM_052889 | 1 | Unknown | other |
| CR2 | complement component (3d/Epstein Barr virus) receptor 2 | -- | 4 | Plasma Membrane | transmembrane receptor |
| CSF3 | colony stimulating factor 3 (granulocyte) | NM_000759 | 1 | Extracellular Space | cytokine |
| CTGF | connective tissue growth factor | NM_001901 | 2 | Extracellular Space | growth factor |
| CTSD | cathepsin D (lysosomal aspartyl peptidase) | -- | 2 | Cytoplasm | peptidase |
| CX3CL1 | chemokine (C-X3-C motif) ligand 1 | -- | 2, 4 | Extracellular Space | cytokine |
| CXCL2 | chemokine (C-X-C motif) ligand 2 | NM_002089 | 1 | Extracellular Space | cytokine |
| CXCL10 | chemokine (C-X-C motif) ligand 10 | NM_001565 | 1 | Extracellular Space | cytokine |
| CXCL11 | chemokine (C-X-C motif) ligand 11 | NM_005409 | 1 | Extracellular Space | cytokine |
| DDIT3 | DNA-damage-inducible transcript 3 | -- | 7 | Nucleus | transcription regulator |
| DSCR1 | Down syndrome critical region gene 1 | -- | 2 | Nucleus | transcription regulator |
| DUSP6 | dual specificity phosphatase 6 | NM_001946 | 2 | Cytoplasm | phosphatase |
| E2F1 | E2F transcription factor 1 | -- | 3 | Nucleus | transcription regulator |
| EDG1 | endothelial differentiation, sphingolipid G-protein-coupled receptor, 1 | -- | 4 | Plasma Membrane | G-protein coupled receptor |
| EPB41 | erythrocyte membrane protein band 4.1 (elliptocytosis 1, RH-linked) | -- | 2 | Plasma Membrane | other |
| ERBB2 | v-erb-b2 erythroblastic leukemia viral oncogene homolog 2, neuro/glioblastoma derived oncogene homolog (avian) | -- | 3 | Plasma Membrane | kinase |
| F2 | coagulation factor II (thrombin) | -- | 2 | Extracellular Space | peptidase |
| F3 | coagulation factor III (thromboplastin, tissue factor) | NM_001993 | 1, 2 | Plasma Membrane | transmembrane receptor |
| FABP5 | fatty acid binding protein 5 (psoriasis-associated) | NM_001444 | 1 | Cytoplasm | transporter |
| FAS | Fas (TNF receptor superfamily, member 6) | -- | 3 | Plasma Membrane | transmembrane receptor |
| FGG | fibrinogen gamma chain | -- | 3 | Extracellular Space | other |
| FLT3LG | fms-related tyrosine kinase 3 ligand | -- | 4 | Extracellular Space | cytokine |
| FSHR | follicle stimulating hormone receptor | -- | 2 | Plasma Membrane | G-protein coupled receptor |
| GADD45B | growth arrest and DNA-damage-inducible, beta | NM_015675 | 1 | Cytoplasm | other |
| GBP1 | guanylate binding protein 1, interferon-inducible, 67kDa | NM_002053 | 1 | Cytoplasm | enzyme |
| GBP2 | guanylate binding protein 2, interferon-inducible | NM_004120 | 1 | Cytoplasm | enzyme |
| GCA | grancalcin, EF-hand calcium binding protein | NM_012198 | 8 | Cytoplasm | other |
| GLP109 | -- | -- | 3 | Unknown | other |
| GLUL | glutamate-ammonia ligase (glutamine synthetase) | AL161952 | 2 | Cytoplasm | enzyme |
| GNAI1 | guanine nucleotide binding protein (G protein), alpha inhibiting activity polypeptide 1 | -- | 4 | Plasma Membrane | enzyme |
| GNB1 | guanine nucleotide binding protein (G protein), beta polypeptide 1 | -- | 4 | Plasma Membrane | enzyme |
| GNB2 | guanine nucleotide binding protein (G protein), beta polypeptide 2 | -- | 4 | Plasma Membrane | enzyme |
| GNB3 | guanine nucleotide binding protein (G protein), beta polypeptide 3 | NM_002075 | 4 | Plasma Membrane | enzyme |
| GNB5 | guanine nucleotide binding protein (G protein), beta 5 | -- | 4 | Plasma Membrane | enzyme |
| GNG3 | guanine nucleotide binding protein (G protein), gamma 3 | -- | 4 | Plasma Membrane | enzyme |
| GNG4 | guanine nucleotide binding protein (G protein), gamma 4 | -- | 4 | Plasma Membrane | enzyme |
| GNG5 | guanine nucleotide binding protein (G protein), gamma 5 | -- | 4 | Plasma Membrane | enzyme |
| GNG7 | guanine nucleotide binding protein (G protein), gamma 7 | -- | 4 | Plasma Membrane | enzyme |
| GNG10 | guanine nucleotide binding protein (G protein), gamma 10 | -- | 4 | Plasma Membrane | enzyme |
| GNG11 | guanine nucleotide binding protein (G protein), gamma 11 | -- | 4 | Plasma Membrane | enzyme |
| GNG12 | guanine nucleotide binding protein (G protein), gamma 12 | -- | 4 | Plasma Membrane | enzyme |
| GNG13 | guanine nucleotide binding protein (G protein), gamma 13 | -- | 4 | Plasma Membrane | enzyme |
| HIF1A | hypoxia-inducible factor 1, alpha subunit (basic helix-loop-helix transcription factor) | NM_001530 | 3 | Nucleus | transcription regulator |
| HP | haptoglobin | AK055872 | 1 | Extracellular Space | peptidase |
| HRASLS3 | HRAS-like suppressor 3 | -- | 3 | Nucleus | other |
| HSPB8 | heat shock 22kDa protein 8 | NM_014365 | 2 | Unknown | kinase |
| ICAM1 | intercellular adhesion molecule 1 (CD54), human rhinovirus receptor | NM_000201 | 1 | Plasma Membrane | transmembrane receptor |
| ID1 | inhibitor of DNA binding 1, dominant negative helix-loop-helix protein | NM_002165 | 2 | Nucleus | other |
| IFNG | interferon, gamma | -- | 3 | Extracellular Space | cytokine |
| IFNK | interferon, kappa | -- | 3 | Extracellular Space | cytokine |
| IL4 | interleukin 4 | -- | 4 | Extracellular Space | cytokine |
| IL6 | interleukin 6 (interferon, beta 2) | NM_000600 | 1 | Extracellular Space | cytokine |
| IL15RA | interleukin 15 receptor, alpha | NM_002189 | 1 | Plasma Membrane | transmembrane receptor |
| IL1B | interleukin 1, beta | NM_000576 | 1 | Extracellular Space | cytokine |
| IL1RN | interleukin 1 receptor antagonist | X52015 | 1 | Extracellular Space | cytokine |
| IRF1 | interferon regulatory factor 1 | NM_002198 | 3 | Nucleus | transcription regulator |
| ITPKC | inositol 1,4,5-trisphosphate 3-kinase C | D38169 |  | Unknown | kinase |
| KIAA0101 | KIAA0101 | -- | 3 | Nucleus | other |
| KIAA0186 | GINS complex subunit 1 (Psf1 homolog) | -- | 3 | Unknown | other |
| KIAA0963 | KIAA0963 | NM_014963 | 1 | Unknown | other |
| KIAA1276 | KIAA1276 protein | AB033102 | 3 | Unknown | other |
| KITLG | KIT ligand | -- | 2 | Extracellular Space | growth factor |
| Klrb1c | killer cell lectin-like receptor subfamily B member 1C | -- | 3 | Plasma Membrane | other |
| KPNA2 | karyopherin alpha 2 (RAG cohort 1, importin alpha 1) | NM_002266 | 2 | Nucleus | transporter |
| LCN2 | lipocalin 2 (oncogene 24p3) | NM_005564 | 1 | Extracellular Space | transporter |
| LCP1 | lymphocyte cytosolic protein 1 (L-plastin) | -- | 8 | Cytoplasm | other |
| LYN | v-yes-1 Yamaguchi sarcoma viral related oncogene homolog | NM_002350 | 3 | Cytoplasm | kinase |
| MAPK3 | mitogen-activated protein kinase 3 | -- | 2 | Cytoplasm | kinase |
| MARCKS | myristoylated alanine-rich protein kinase C substrate | NM_002356 | 2 | Plasma Membrane | other |
| MATK | megakaryocyte-associated tyrosine kinase | -- | 3 | Cytoplasm | kinase |
| MLLT3 | myeloid/lymphoid or mixed-lineage leukemia (trithorax homolog, Drosophila); translocated to, 3 | NM_004529 | 5 | Nucleus | other |
| MME | membrane metallo-endopeptidase (neutral endopeptidase, enkephalinase, CALLA, CD10) | -- | 2 | Plasma Membrane | peptidase |
| MYC | v-myc myelocytomatosis viral oncogene homolog (avian) | -- | 2 | Nucleus | transcription regulator |
| MYH9 | myosin, heavy polypeptide 9, non-muscle | -- | 2 | Cytoplasm | other |
| NDRG2 | NDRG family member 2 | NM_016250 | 4 | Cytoplasm | other |
| NFKBIA | nuclear factor of kappa light polypeptide gene enhancer in B-cells inhibitor, alpha | NM_020529 | 1 | Cytoplasm | other |
| NFKBIE | nuclear factor of kappa light polypeptide gene enhancer in B-cells inhibitor, epsilon | NM_004556 | 1 | Nucleus | transcription regulator |
| NFYB | nuclear transcription factor Y, beta | -- | 3 | Nucleus | transcription regulator |
| NP | nucleoside phosphorylase | NM_000270 | 4 | Nucleus | enzyme |
| NUP88 | nucleoporin 88kDa | NM_002532 | 6 | Nucleus | transporter |
| NUP214 | nucleoporin 214kDa | -- | 6 | Nucleus | transporter |
| OAS1 | 2',5'-oligoadenylate synthetase 1, 40/46kDa | -- | 3 | Cytoplasm | enzyme |
| OAS2 | 2'-5'-oligoadenylate synthetase 2, 69/71kDa | -- | 3 | Cytoplasm | enzyme |
| P2RY2 | purinergic receptor P2Y, G-protein coupled, 2 | -- | 2 | Plasma Membrane | G-protein coupled receptor |
| PDE4A | phosphodiesterase 4A, cAMP-specific (phosphodiesterase E2 dunce homolog, Drosophila) | -- | 3 | Cytoplasm | enzyme |
| PDGFB | platelet-derived growth factor beta polypeptide (simian sarcoma viral (v-sis) oncogene homolog) | -- | 2 | Extracellular Space | growth factor |
| PDK4 | pyruvate dehydrogenase kinase, isozyme 4 | AF334710 | 3 | Cytoplasm | kinase |
| PHLDA1 | pleckstrin homology-like domain, family A, member 1 | -- | 3 | Cytoplasm | other |
| PIK3CG | phosphoinositide-3-kinase, catalytic, gamma polypeptide | -- | 4 | Cytoplasm | kinase |
| PLCB2 | phospholipase C, beta 2 | -- | 4 | Cytoplasm | enzyme |
| PLCE1 | phospholipase C, epsilon 1 | -- | 4 | Cytoplasm | enzyme |
| POLE3 | polymerase (DNA directed), epsilon 3 (p17 subunit) | -- | 9 | Nucleus | enzyme |
| PP | pyrophosphatase (inorganic) 1 | NM_021129 | 3 | Cytoplasm | enzyme |
| PPARA | peroxisome proliferative activated receptor, alpha | -- | 3 | Nucleus | ligand-dependent nuclear receptor |
| PPIA | peptidylprolyl isomerase A (cyclophilin A) | -- | 4 | Cytoplasm | enzyme |
| PRG1 | proteoglycan 1, secretory granule | NM_002727 | 2 | Extracellular Space | other |
| PRPSAP1 | phosphoribosyl pyrophosphate synthetase-associated protein 1 | NM_002766 | 3 | Unknown | other |
| PRSS25 | HtrA serine peptidase 2 | -- | 4 | Cytoplasm | peptidase |
| PTN | pleiotrophin (heparin binding growth factor 8, neurite growth-promoting factor 1) | -- | 2 | Extracellular Space | growth factor |
| RAB3C | RAB3C, member RAS oncogene family | -- | 3 | Cytoplasm | enzyme |
| RGS3 | regulator of G-protein signalling 3 | -- | 4 | Nucleus | other |
| RIPK2 | receptor-interacting serine-threonine kinase 2 | NM_003821 | 1 | Plasma Membrane | kinase |
| S100A8 | S100 calcium binding protein A8 (calgranulin A) | NM_002964 | 1 | Cytoplasm | other |
| S100A9 | S100 calcium binding protein A9 (calgranulin B) | NM_002965 | 1 | Cytoplasm | other |
| SDC4 | syndecan 4 (amphiglycan, ryudocan) | NM_002999 | 2 | Plasma Membrane | other |
| SERPINA3 | serpin peptidase inhibitor, clade A (alpha-1 antiproteinase, antitrypsin), member 3 | NM_001085 | 3 | Extracellular Space | other |
| SLC39A8 | solute carrier family 39 (zinc transporter), member 8 | NM_022154 | 3 | Unknown | transporter |
| SMARCA1 | SWI/SNF related, matrix associated, actin dependent regulator of chromatin, subfamily a, member 1 | -- | 9 | Nucleus | transcription regulator |
| SMARCA5 | SWI/SNF related, matrix associated, actin dependent regulator of chromatin, subfamily a, member 5 | -- | 9 | Nucleus | transcription regulator |
| SMPD2 | sphingomyelin phosphodiesterase 2, neutral membrane (neutral sphingomyelinase) | -- | 2 | Cytoplasm | enzyme |
| SOD2 | superoxide dismutase 2, mitochondrial | NM_000636 | 1 | Cytoplasm | enzyme |
| SPARCL1 | SPARC-like 1 (mast9, hevin) | NM_004684 | 3 | Extracellular Space | other |
| SRI | sorcin | -- | 8 | Cytoplasm | transporter |
| TFPI | tissue factor pathway inhibitor (lipoprotein-associated coagulation inhibitor) | -- | 2 | Extracellular Space | other |
| THBD | thrombomodulin | NM_000361 | 1 | Plasma Membrane | transmembrane receptor |
| TNFAIP2 | tumor necrosis factor, alpha-induced protein 2 | NM_006291 | 1 | Extracellular Space | other |
| TNFAIP3 | tumor necrosis factor, alpha-induced protein 3 | NM_006290 | 1 | Nucleus | other |
| TNIP1 | TNFAIP3 interacting protein 1 | NM_006058 | 1 | Nucleus | other |
| TSC22D3 | TSC22 domain family, member 3 | AB025432 | 2 | Nucleus | transcription regulator |
| TXNIP | thioredoxin interacting protein | -- | 2 | Cytoplasm | other |
| TXNRD1 | thioredoxin reductase 1 | NM_003330 | 3 | Cytoplasm | enzyme |
| VCAM1 | vascular cell adhesion molecule 1 | NM_001078 | 1 | Plasma Membrane | other |
| WARS | tryptophanyl-tRNA synthetase | NM_004184 | 3 | Cytoplasm | enzyme |
| YY1 | YY1 transcription factor | AK026497 | 4 | Nucleus | transcription regulator |
| ZFP36 | zinc finger protein 36, C3H type, homolog (mouse) | NM_003407 | 3 | Nucleus | transcription regulator |
