# Supplementary material for: Temporal dynamics of gene expression in the lung in a baboon model of E. coli sepsis
Source: BMC Genomics. 2007 Feb 26;8:58. doi: 10.1186/1471-2164-8-58 (PMC1819384; doi:10.1186/1471-2164-8-58)
Supplement: Additional file 3 — Table_A3. The table lists the genes of the IPA networks at the time point 8 hrs. [file 1471-2164-8-58-S3.doc]

***Table A3:* Genes included in the IPA networks at 8 hrs**

| ***Name*** | ***Description*** | ***Genbank*** | ***Ntw.*** | ***Location*** | ***Family*** |
| --- | --- | --- | --- | --- | --- |
| A2M | alpha-2-macroglobulin | NM_000014 | 1 | Extracellular Space | transporter |
| ACTB | actin, beta | -- | 8 | Cytoplasm | other |
| ADAMTS1 | ADAM metallopeptidase with thrombospondin type 1 motif, 1 | -- | 1 | Extracellular Space | peptidase |
| ADAMTS4 | ADAM metallopeptidase with thrombospondin type 1 motif, 4 | -- | 6 | Extracellular Space | peptidase |
| ADCYAP1 | adenylate cyclase activating polypeptide 1 (pituitary) | NM_001117 | 1 | Extracellular Space | other |
| ADM | adrenomedullin | -- | 2 | Extracellular Space | other |
| AKT1 | v-akt murine thymoma viral oncogene homolog 1 | -- | 5 | Cytoplasm | kinase |
| AP3D1 | adaptor-related protein complex 3, delta 1 subunit | -- | 13 | Cytoplasm | transporter |
| ARHGAP24 | Rho GTPase activating protein 24 | NM_031305 |  | Cytoplasm | other |
| ARHGEF9 | Cdc42 guanine nucleotide exchange factor (GEF) 9 | -- | 14 | Cytoplasm | other |
| ARL6 | ADP-ribosylation factor-like 6 | -- | 4 | Cytoplasm | transporter |
| ARL6IP5 | ADP-ribosylation-like factor 6 interacting protein 5 | NM_006407 | 4 | Cytoplasm | other |
| ASNS | asparagine synthetase | -- | 4 | Unknown | enzyme |
| BCAT2 | branched chain aminotransferase 2, mitochondrial | -- | 8 | Cytoplasm | enzyme |
| BHLHB2 | basic helix-loop-helix domain containing, class B, 2 | -- | 6 | Nucleus | transcription regulator |
| BRAF | v-raf murine sarcoma viral oncogene homolog B1 | NM_004333 | 7 | Cytoplasm | enzyme |
| C20ORF24 | chromosome 20 open reading frame 24 | NM_018840 | 6 | Cytoplasm | other |
| CA4 | carbonic anhydrase IV | NM_000717 |  | Plasma Membrane | enzyme |
| CALD1 | caldesmon 1 | -- | 6 | Cytoplasm | other |
| CASP3 | caspase 3, apoptosis-related cysteine peptidase | -- | 2 | Cytoplasm | peptidase |
| CCL3L1 | chemokine (C-C motif) ligand 3-like 1 | D90145 | 4 | Extracellular Space | cytokine |
| CD9 | CD9 molecule | NM_001769 | 1 | Plasma Membrane | other |
| CD81 | CD81 molecule | -- | 2 | Plasma Membrane | other |
| CD160 | CD160 molecule | NM_007053 | 2 | Plasma Membrane | transmembrane receptor |
| CD200 | CD200 molecule | NM_005944 | 7 | Plasma Membrane | other |
| CD200R1 | CD200 receptor 1 | -- | 7 | Plasma Membrane | other |
| CDC2L6 | cell division cycle 2-like 6 (CDK8-like) | AL122055 |  | Nucleus | kinase |
| CDKN2A | cyclin-dependent kinase inhibitor 2A (melanoma, p16, inhibits CDK4) | -- | 5 | Nucleus | transcription regulator |
| CEBPD | CCAAT/enhancer binding protein (C/EBP), delta | NM_005195 | 1, 3 | Nucleus | transcription regulator |
| CENTD1 | centaurin, delta 1 | NM_015230 | 2 | Unknown | other |
| CENTG1 | centaurin, gamma 1 | NM_014770 | 5 | Nucleus | enzyme |
| CFL1 | cofilin 1 (non-muscle) | -- | 1 | Nucleus | other |
| CHD2 | chromodomain helicase DNA binding protein 2 | NM_001271 | 5 | Nucleus | enzyme |
| CHI3L1 | chitinase 3-like 1 (cartilage glycoprotein-39) | NM_001276 | 6 | Extracellular Space | enzyme |
| CHTF18 | CTF18, chromosome transmission fidelity factor 18 homolog (S. cerevisiae) | -- | 5 | Unknown | other |
| CLDN4 | claudin 4 | NM_001305 | 1 | Plasma Membrane | transmembrane receptor |
| CLEC5A | C-type lectin domain family 5, member A | AF139768 | 11 | Plasma Membrane | other |
| CLIC5 | chloride intracellular channel 5 | AL049313 | 7 | Cytoplasm | ion channel |
| CLN3 | ceroid-lipofuscinosis, neuronal 3, juvenile (Batten, Spielmeyer-Vogt disease) | NM_000086 | 13 | Cytoplasm | other |
| CLN5 | ceroid-lipofuscinosis, neuronal 5 | -- | 13 | Cytoplasm | other |
| CLTC | clathrin, heavy polypeptide (Hc) | -- | 7 | Plasma Membrane | other |
| COL16A1 | collagen, type XVI, alpha 1 | -- | 6 | Extracellular Space | other |
| COL3A1 | collagen, type III, alpha 1 (Ehlers-Danlos syndrome type IV, autosomal dominant) | -- | 7 | Extracellular Space | other |
| COL6A1 | collagen, type VI, alpha 1 | NM_001848 | 6 | Extracellular Space | other |
| COL6A2 | collagen, type VI, alpha 2 | -- | 6 | Extracellular Space | other |
| COL6A3 | collagen, type VI, alpha 3 | -- | 6 | Extracellular Space | other |
| COP1 | caspase-1 dominant-negative inhibitor pseudo-ICE | NM_052889 | 6 | Unknown | other |
| CPB2 | carboxypeptidase B2 (plasma, carboxypeptidase U) | -- | 6 | Extracellular Space | peptidase |
| CPM | carboxypeptidase M | AF368463 | 12 | Plasma Membrane | peptidase |
| CPN2 | carboxypeptidase N, polypeptide 2, 83kD | J05158 | 5 | Extracellular Space | peptidase |
| CRKL | v-crk sarcoma virus CT10 oncogene homolog (avian)-like | -- | 7 | Cytoplasm | kinase |
| CTSD | cathepsin D (lysosomal aspartyl peptidase) | -- | 2 | Cytoplasm | peptidase |
| CTSH | cathepsin H | -- | 8 | Cytoplasm | peptidase |
| CXCL3 | chemokine (C-X-C motif) ligand 3 | -- | 1 | Extracellular Space | cytokine |
| CXCL5 | chemokine (C-X-C motif) ligand 5 | NM_002994 | 1, 4 | Extracellular Space | cytokine |
| CXCL11 | chemokine (C-X-C motif) ligand 11 | NM_005409 | 3 | Extracellular Space | cytokine |
| CYP1A2 | cytochrome P450, family 1, subfamily A, polypeptide 2 | NM_000761 | 4 | Cytoplasm | enzyme |
| DAPK1 | death-associated protein kinase 1 | -- | 6 | Cytoplasm | kinase |
| DARC | Duffy blood group, chemokine receptor | NM_002036 | 1 | Plasma Membrane | G-protein coupled receptor |
| DCC1 | defective in sister chromatid cohesion homolog 1 (S. cerevisiae) | -- | 5 | Unknown | other |
| DDX5 | DEAD (Asp-Glu-Ala-Asp) box polypeptide 5 | -- | 3 | Nucleus | enzyme |
| DDX17 | DEAD (Asp-Glu-Ala-Asp) box polypeptide 17 | -- | 8 | Nucleus | enzyme |
| DERPC | chromosome transmission fidelity factor 8 homolog (S. cerevisiae) | BC018700 | 5 | Unknown | other |
| DIO1 | deiodinase, iodothyronine, type I | -- | 4, 8 | Cytoplasm | enzyme |
| DLL4 | delta-like 4 (Drosophila) | -- | 4 | Extracellular Space | other |
| DOK1 | docking protein 1, 62kDa (downstream of tyrosine kinase 1) | -- | 7 | Plasma Membrane | other |
| DOK2 | docking protein 2, 56kDa | -- | 3, 7 | Plasma Membrane | other |
| E2F1 | E2F transcription factor 1 | -- | 5 | Nucleus | transcription regulator |
| EGF | epidermal growth factor (beta-urogastrone) | -- | 7 | Extracellular Space | growth factor |
| EHHADH | enoyl-Coenzyme A, hydratase/3-hydroxyacyl Coenzyme A dehydrogenase | -- | 8 | Cytoplasm | enzyme |
| ELP5 | -- | -- | 5 | Unknown | other |
| EMP2 | epithelial membrane protein 2 | NM_001424 | 2 | Plasma Membrane | other |
| EPB41L2 | erythrocyte membrane protein band 4.1-like 2 | NM_001431 | 7 | Plasma Membrane | other |
| EPRS | glutamyl-prolyl-tRNA synthetase | -- | 6 | Cytoplasm | enzyme |
| F2 | coagulation factor II (thrombin) | -- | 6 | Extracellular Space | peptidase |
| FABP5 | fatty acid binding protein 5 (psoriasis-associated) | NM_001444 | 2 | Cytoplasm | transporter |
| FAP | fibroblast activation protein, alpha | AK055327 | 2 | Cytoplasm | peptidase |
| FLII | flightless I homolog (Drosophila) | -- | 8 | Nucleus | other |
| FLT1 | fms-related tyrosine kinase 1 (vascular endothelial growth factor/vascular permeability factor receptor) | AF339822 | 4 | Plasma Membrane | kinase |
| FMNL1 | formin-like 1 | NM_005892 |  | Cytoplasm | other |
| FMR1 | fragile X mental retardation 1 | -- | 8 | Nucleus | other |
| FN1 | fibronectin 1 | NM_002026 | 1 | Plasma Membrane | enzyme |
| FURIN | furin (paired basic amino acid cleaving enzyme) | -- | 6 | Cytoplasm | peptidase |
| GBP1 | guanylate binding protein 1, interferon-inducible, 67kDa | NM_002053 | 2, 4 | Cytoplasm | enzyme |
| GBP2 | guanylate binding protein 2, interferon-inducible | NM_004120 | 2, 3 | Cytoplasm | enzyme |
| GCM2 | glial cells missing homolog 2 (Drosophila) | NM_004752 | 3 | Nucleus | transcription regulator |
| GDI1 | GDP dissociation inhibitor 1 | NM_001493 | 8 | Cytoplasm | other |
| GGA2 | golgi associated, gamma adaptin ear containing, ARF binding protein 2 | -- | 7 | Cytoplasm | transporter |
| GH1 | growth hormone 1 | -- | 3 | Extracellular Space | cytokine |
| GHRHR | growth hormone releasing hormone receptor | -- | 8 | Plasma Membrane | G-protein coupled receptor |
| GLRA1 | glycine receptor, alpha 1 (startle disease/hyperekplexia, stiff man syndrome) | -- | 14 | Plasma Membrane | ion channel |
| GLRA2 | glycine receptor, alpha 2 | -- | 14 | Plasma Membrane | ion channel |
| GLRA3 | glycine receptor, alpha 3 | -- | 14 | Plasma Membrane | ion channel |
| GLRA4 | glycine receptor, alpha 4 | -- | 14 | Plasma Membrane | ion channel |
| GLRB | glycine receptor, beta | NM_000824 | 14 | Plasma Membrane | ion channel |
| GM2A | GM2 ganglioside activator | -- | 3 | Cytoplasm | enzyme |
| GNB1 | guanine nucleotide binding protein (G protein), beta polypeptide 1 | -- | 5 | Plasma Membrane | enzyme |
| GNB3 | guanine nucleotide binding protein (G protein), beta polypeptide 3 | NM_002075 | 5 | Plasma Membrane | enzyme |
| GNB5 | guanine nucleotide binding protein (G protein), beta 5 | BC011671 | 5 | Plasma Membrane | enzyme |
| GNG5 | guanine nucleotide binding protein (G protein), gamma 5 | -- | 5 | Plasma Membrane | enzyme |
| GNG7 | guanine nucleotide binding protein (G protein), gamma 7 | -- | 5 | Plasma Membrane | enzyme |
| GNG10 | guanine nucleotide binding protein (G protein), gamma 10 | -- | 5 | Plasma Membrane | enzyme |
| GNG11 | guanine nucleotide binding protein (G protein), gamma 11 | -- | 5 | Plasma Membrane | enzyme |
| GNG12 | guanine nucleotide binding protein (G protein), gamma 12 | AK055914 | 5 | Plasma Membrane | enzyme |
| GPHN | gephyrin | -- | 14 | Plasma Membrane | enzyme |
| GSN | gelsolin (amyloidosis, Finnish type) | -- | 7 | Extracellular Space | other |
| GSTP1 | glutathione S-transferase pi | -- | 3 | Cytoplasm | enzyme |
| GTF2A1 | general transcription factor IIA, 1, 19/37kDa | NM_015859 | 4 | Cytoplasm | transcription regulator |
| GTF2A2 | general transcription factor IIA, 2, 12kDa | -- | 4 | Nucleus | transcription regulator |
| HCCA2 | HCCA2 protein | NM_053005 | 8 | Nucleus | other |
| HERC1 | hect (homologous to the E6-AP (UBE3A) carboxyl terminus) domain and RCC1 (CHC1)-like domain (RLD) 1 | NM_003922 | 7 | Cytoplasm | other |
| HGF | hepatocyte growth factor (hepapoietin A; scatter factor) | -- | 5 | Extracellular Space | growth factor |
| HHEX | homeobox, hematopoietically expressed | -- | 4 | Nucleus | transcription regulator |
| HIF1A | hypoxia-inducible factor 1, alpha subunit (basic helix-loop-helix transcription factor) | -- | 4 | Nucleus | transcription regulator |
| HIST2H4 | histone 2, H4 | -- | 4 | Nucleus | other |
| HLA-C | major histocompatibility complex, class I, C | -- | 2 | Plasma Membrane | transmembrane receptor |
| HLA-G | HLA-G histocompatibility antigen, class I, G | -- | 2 | Plasma Membrane | transmembrane receptor |
| HMGN2 | high-mobility group nucleosomal binding domain 2 | -- | 3 | Nucleus | other |
| HP | haptoglobin | AK055872 | 1 | Extracellular Space | peptidase |
| HR | hairless homolog (mouse) | -- | 8 | Nucleus | transcription regulator |
| HSPA1A | heat shock 70kDa protein 1A | -- | 3 | Cytoplasm | other |
| HSPB8 | heat shock 22kDa protein 8 | NM_014365 | 2 | Unknown | kinase |
| IARS | isoleucine-tRNA synthetase | NM_013417 | 6 | Cytoplasm | enzyme |
| IER3 | immediate early response 3 | -- | 3 | Cytoplasm | other |
| IFNG | interferon, gamma | -- | 2 | Extracellular Space | cytokine |
| IGF2 | insulin-like growth factor 2 (somatomedin A) | -- | 8 | Extracellular Space | growth factor |
| IHPK1 | inositol hexaphosphate kinase 1 | AK001120 |  | Cytoplasm | kinase |
| IL6 | interleukin 6 (interferon, beta 2) | -- | 4 | Extracellular Space | cytokine |
| IL13 | interleukin 13 | -- | 7 | Extracellular Space | cytokine |
| IL1B | interleukin 1, beta | -- | 6 | Extracellular Space | cytokine |
| INDO | indoleamine-pyrrole 2,3 dioxygenase | NM_002164 | 2 | Cytoplasm | enzyme |
| IRF1 | interferon regulatory factor 1 | NM_002198 | 1 | Nucleus | transcription regulator |
| ITGAL | integrin, alpha L (antigen CD11A (p180), lymphocyte function-associated antigen 1; alpha polypeptide) | -- | 3 | Plasma Membrane | other |
| ITGB1 | integrin, beta 1 (fibronectin receptor, beta polypeptide, antigen CD29 includes MDF2, MSK12) | -- | 2 | Plasma Membrane | transmembrane receptor |
| ITGB1BP1 | integrin beta 1 binding protein 1 | NM_004763 | 1 | Plasma Membrane | other |
| JAM2 | junctional adhesion molecule 2 | NM_021219 | 2 | Plasma Membrane | other |
| KLF2 | Kruppel-like factor 2 (lung) | -- | 3 | Nucleus | transcription regulator |
| KRT8 | keratin 8 | -- | 7 | Cytoplasm | kinase |
| KRT18 | keratin 18 | -- | 7 | Cytoplasm | other |
| KRT19 | keratin 19 | NM_002276 | 7 | Cytoplasm | other |
| LAP3 | leucine aminopeptidase 3 | NM_015907 |  | Cytoplasm | peptidase |
| LARGE | like-glycosyltransferase | NM_004737 | 4 | Cytoplasm | enzyme |
| LCAT | lecithin-cholesterol acyltransferase | -- | 1 | Extracellular Space | enzyme |
| LCN2 | lipocalin 2 (oncogene 24p3) | NM_005564 | 5 | Extracellular Space | transporter |
| LDB2 | LIM domain binding 2 | NM_001290 | 3 | Nucleus | transcription regulator |
| LRPAP1 | low density lipoprotein receptor-related protein associated protein 1 | -- | 1 | Plasma Membrane | transmembrane receptor |
| LRRN1 | leucine rich repeat neuronal 1 | AB040930 | 3 | Unknown | other |
| MAFK | v-maf musculoaponeurotic fibrosarcoma oncogene homolog K (avian) | AK056767 | 3 | Nucleus | transcription regulator |
| MAGEA3 | melanoma antigen family A, 3 | -- | 2 | Unknown | other |
| MAP2K2 | mitogen-activated protein kinase kinase 2 | -- | 7 | Cytoplasm | kinase |
| MDM2 | Mdm2, transformed 3T3 cell double minute 2, p53 binding protein (mouse) | -- | 10 | Nucleus | transcription regulator |
| MGP | matrix Gla protein | NM_000900 | 1 | Extracellular Space | other |
| MIA | melanoma inhibitory activity | -- | 1 | Extracellular Space | other |
| MIF | macrophage migration inhibitory factor (glycosylation-inhibiting factor) | -- | 2 | Extracellular Space | cytokine |
| MKL1 | megakaryoblastic leukemia (translocation) 1 | AK025156 | 3 | Nucleus | transcription regulator |
| MMP2 | matrix metallopeptidase 2 (gelatinase A, 72kDa gelatinase, 72kDa type IV collagenase) | NM_004530 | 1 | Extracellular Space | peptidase |
| MMP16 | matrix metallopeptidase 16 (membrane-inserted) | -- | 1 | Extracellular Space | peptidase |
| MMP17 | matrix metallopeptidase 17 (membrane-inserted) | -- | 1 | Extracellular Space | peptidase |
| MMP25 | matrix metallopeptidase 25 | -- | 1 | Extracellular Space | peptidase |
| MMP26 | matrix metallopeptidase 26 | -- | 1 | Extracellular Space | peptidase |
| MTBP | Mdm2, transformed 3T3 cell double minute 2, p53 binding protein (mouse) binding protein, 104kDa | AK025100 | 10 | Unknown | other |
| MYBBP1A | MYB binding protein (P160) 1a | NM_014520 | 5 | Nucleus | transcription regulator |
| MYC | v-myc myelocytomatosis viral oncogene homolog (avian) | -- | 3 | Nucleus | transcription regulator |
| NCOA2 | nuclear receptor coactivator 2 | NM_006540 | 8 | Nucleus | transcription regulator |
| NDRG2 | NDRG family member 2 | NM_016250 | 3 | Cytoplasm | other |
| NEDD9 | neural precursor cell expressed, developmentally down-regulated 9 | -- | 2 | Nucleus | other |
| NF2 | neurofibromin 2 (bilateral acoustic neuroma) | -- | 7 | Plasma Membrane | other |
| NFYB | nuclear transcription factor Y, beta | -- | 8 | Nucleus | transcription regulator |
| NME1 | non-metastatic cells 1, protein (NM23A) expressed in | -- | 8 | Nucleus | kinase |
| NME2 | non-metastatic cells 2, protein (NM23B) expressed in | -- | 1 | Nucleus | kinase |
| NNMT | nicotinamide N-methyltransferase | -- | 6 | Cytoplasm | enzyme |
| NNT | nicotinamide nucleotide transhydrogenase | AF052090 |  | Cytoplasm | enzyme |
| NOC2L | nucleolar complex associated 2 homolog (S. cerevisiae) | NM_015658 | 4 | Nucleus | transcription regulator |
| NPPC | natriuretic peptide precursor C | NM_024409 | 6 | Extracellular Space | other |
| NRGN | neurogranin (protein kinase C substrate, RC3) | -- | 8 | Cytoplasm | other |
| NUFIP1 | nuclear fragile X mental retardation protein interacting protein 1 | NM_012345 | 8 | Nucleus | other |
| NUP88 | nucleoporin 88kDa | NM_002532 | 1 | Nucleus | transporter |
| OAS1 | 2',5'-oligoadenylate synthetase 1, 40/46kDa | -- | 3 | Cytoplasm | enzyme |
| P2RX7 | purinergic receptor P2X, ligand-gated ion channel, 7 | -- | 2 | Plasma Membrane | ion channel |
| PAPSS1 | 3'-phosphoadenosine 5'-phosphosulfate synthase 1 | -- | 9 | Cytoplasm | enzyme |
| PAPSS2 | 3'-phosphoadenosine 5'-phosphosulfate synthase 2 | AF074331 | 9 | Cytoplasm | enzyme |
| PAX3 | paired box gene 3 (Waardenburg syndrome 1) | -- | 5 | Nucleus | transcription regulator |
| PBEF1 | pre-B-cell colony enhancing factor 1 | -- | 6 | Extracellular Space | cytokine |
| PCNA | proliferating cell nuclear antigen | -- | 5 | Nucleus | other |
| PENK1 | proenkephalin 1 | -- | 8 | Extracellular Space | other |
| PF4 | platelet factor 4 (chemokine (C-X-C motif) ligand 4) | -- | 12 | Extracellular Space | cytokine |
| PFKFB1 | 6-phosphofructo-2-kinase/fructose-2,6-biphosphatase 1 | NM_002625 | 3 | Cytoplasm | kinase |
| PHF6 | PHD finger protein 6 | AB058726 |  | Unknown | other |
| PHLDA2 | pleckstrin homology-like domain, family A, member 2 | NM_003311 | 7 | Unknown | other |
| PHOX2A | paired-like (aristaless) homeobox 2a | -- | 4 | Nucleus | transcription regulator |
| PHOX2B | paired-like homeobox 2b | NM_003924 | 4 | Nucleus | transcription regulator |
| PIK3C2A | phosphoinositide-3-kinase, class 2, alpha polypeptide | AL049998 | 7 | Cytoplasm | kinase |
| PKP4 | plakophilin 4 | AL050364 | 6 | Plasma Membrane | other |
| PLA2G1B | phospholipase A2, group IB (pancreas) | -- | 1 | Extracellular Space | enzyme |
| PLAGL1 | pleiomorphic adenoma gene-like 1 | -- | 8 | Nucleus | transcription regulator |
| PLCL1 | phospholipase C-like 1 | -- | 5 | Cytoplasm | enzyme |
| PMAIP1 | phorbol-12-myristate-13-acetate-induced protein 1 | -- | 3, 4 | Cytoplasm | other |
| PPA1 | pyrophosphatase (inorganic) 1 | NM_021129 |  | Cytoplasm | enzyme |
| PPARBP | PPAR binding protein | -- | 4 | Nucleus | transcription regulator |
| PPFIA4 | protein tyrosine phosphatase, receptor type, f polypeptide (PTPRF), interacting protein (liprin), alpha 4 | AB020704 | 4 | Plasma Membrane | phosphatase |
| PPP1R14B | protein phosphatase 1, regulatory (inhibitor) subunit 14B | -- | 4 | Unknown | phosphatase |
| PRDX6 | peroxiredoxin 6 | -- | 1 | Cytoplasm | enzyme |
| PRG1 (includes EG:5552) | proteoglycan 1, secretory granule | NM_002727 | 5 | Extracellular Space | other |
| PROCR | protein C receptor, endothelial (EPCR) | -- | 6 | Plasma Membrane | other |
| PRPF3 | PRP3 pre-mRNA processing factor 3 homolog (S. cerevisiae) | -- | 7 | Nucleus | other |
| PRPF4 | PRP4 pre-mRNA processing factor 4 homolog (yeast) | NM_004697 | 7 | Nucleus | other |
| PSEN1 | presenilin 1 (Alzheimer disease 3) | -- | 6 | Plasma Membrane | peptidase |
| PSMB7 | proteasome (prosome, macropain) subunit, beta type, 7 | -- | 2 | Cytoplasm | peptidase |
| PSMB8 | proteasome (prosome, macropain) subunit, beta type, 8 (large multifunctional peptidase 7) | NM_004159 | 2, 3 | Cytoplasm | peptidase |
| PSMB9 | proteasome (prosome, macropain) subunit, beta type, 9 (large multifunctional peptidase 2) | -- | 2 | Cytoplasm | peptidase |
| PSMB10 | proteasome (prosome, macropain) subunit, beta type, 10 | -- | 2 | Cytoplasm | peptidase |
| PSME1 | proteasome (prosome, macropain) activator subunit 1 (PA28 alpha) | -- | 2 | Cytoplasm | other |
| PSME2 | proteasome (prosome, macropain) activator subunit 2 (PA28 beta) | NM_002818 | 2 | Cytoplasm | peptidase |
| PTEN | phosphatase and tensin homolog (mutated in multiple advanced cancers 1) | -- | 3 | Cytoplasm | phosphatase |
| RAB3A | RAB3A, member RAS oncogene family | -- | 8 | Cytoplasm | enzyme |
| RAB5C | RAB5C, member RAS oncogene family | NM_004583 | 5 | Cytoplasm | enzyme |
| RAMP2 | receptor (calcitonin) activity modifying protein 2 | NM_005854 | 2 | Plasma Membrane | other |
| RBBP4 | retinoblastoma binding protein 4 | NM_005610 | 5 | Nucleus | other |
| RECK | reversion-inducing-cysteine-rich protein with kazal motifs | -- | 1 | Plasma Membrane | other |
| RFC3 | replication factor C (activator 1) 3, 38kDa | -- | 5 | Nucleus | enzyme |
| RFC4 | replication factor C (activator 1) 4, 37kDa | -- | 5 | Nucleus | other |
| RFC5 | replication factor C (activator 1) 5, 36.5kDa | -- | 5 | Nucleus | enzyme |
| RGC32 | response gene to complement 32 | NM_014059 | 1 | Cytoplasm | other |
| RGS3 | regulator of G-protein signalling 3 | -- | 5 | Nucleus | other |
| RIT2 | Ras-like without CAAX 2 | -- | 7 | Plasma Membrane | enzyme |
| RNF4 | ring finger protein 4 | NM_002938 | 4 | Nucleus | transcription regulator |
| RPL22 | ribosomal protein L22 | AF113701 | 3 | Nucleus | other |
| RPS27A | ribosomal protein S27a | NM_002954 | 6 | Cytoplasm | other |
| RPSA | ribosomal protein SA | -- | 1, 8 | Plasma Membrane | transmembrane receptor |
| RREB1 | ras responsive element binding protein 1 | D49835 | 5 | Nucleus | transcription regulator |
| RUNX2 | runt-related transcription factor 2 | -- | 3 | Nucleus | transcription regulator |
| S100A8 | S100 calcium binding protein A8 (calgranulin A) | NM_002964 | 2 | Cytoplasm | other |
| S100A9 | S100 calcium binding protein A9 (calgranulin B) | NM_002965 | 2 | Cytoplasm | other |
| SART3 | squamous cell carcinoma antigen recognised by T cells 3 | -- | 7 | Nucleus | other |
| SCARB1 | scavenger receptor class B, member 1 | -- | 2 | Plasma Membrane | transporter |
| SEMA4A | sema domain, immunoglobulin domain (Ig), transmembrane domain (TM) and short cytoplasmic domain, (semaphorin) 4A | AB029394 | 4 | Plasma Membrane | other |
| SERPINA3 | serpin peptidase inhibitor, clade A (alpha-1 antiproteinase, antitrypsin), member 3 | NM_001085 | 1 | Extracellular Space | other |
| SFRP1 | secreted frizzled-related protein 1 | -- | 3 | Plasma Membrane | transmembrane receptor |
| SFTPB | surfactant, pulmonary-associated protein B | NM_000542 | 8 | Extracellular Space | other |
| SFTPC | surfactant, pulmonary-associated protein C | -- | 8 | Extracellular Space | other |
| SGK | serum/glucocorticoid regulated kinase | -- | 7 | Cytoplasm | kinase |
| SHMT2 | serine hydroxymethyltransferase 2 (mitochondrial) | AK055053 | 3 | Cytoplasm | enzyme |
| SKIL | SKI-like | -- | 6 | Nucleus | transcription regulator |
| SLC2A1 | solute carrier family 2 (facilitated glucose transporter), member 1 | -- | 3 | Plasma Membrane | transporter |
| SLC2A9 | solute carrier family 2 (facilitated glucose transporter), member 9 | NM_020041 | 6 | Plasma Membrane | transporter |
| SLC30A1 | solute carrier family 30 (zinc transporter), member 1 | NM_021194 | 8 | Plasma Membrane | transporter |
| SLC39A8 | solute carrier family 39 (zinc transporter), member 8 | NM_022154 | 8 | Unknown | transporter |
| SLC39A14 | solute carrier family 39 (zinc transporter), member 14 | D31887 | 6 | Unknown | transporter |
| SLC9A1 | solute carrier family 9 (sodium/hydrogen exchanger), member 1 (antiporter, Na+/H+, amiloride sensitive) | -- | 7 | Plasma Membrane | ion channel |
| SMAD2 | SMAD, mothers against DPP homolog 2 (Drosophila) | -- | 6 | Nucleus | transcription regulator |
| SMARCB1 | SWI/SNF related, matrix associated, actin dependent regulator of chromatin, subfamily b, member 1 | -- | 5 | Nucleus | other |
| SORT1 | sortilin 1 | AK000757 | 7 | Cytoplasm | transporter |
| SOX8 | SRY (sex determining region Y)-box 8 | AK024491 | 3 | Nucleus | transcription regulator |
| SP1 | Sp1 transcription factor | -- | 4 | Nucleus | transcription regulator |
| SP100 | SP100 nuclear antigen | -- | 2 | Nucleus | other |
| SPARCL1 | SPARC-like 1 (mast9, hevin) | NM_004684 | 6 | Extracellular Space | other |
| SPRY2 | sprouty homolog 2 (Drosophila) | -- | 7 | Plasma Membrane | other |
| SPRY4 | sprouty homolog 4 (Drosophila) | -- | 7 | Plasma Membrane | other |
| STK16 | serine/threonine kinase 16 | -- | 6 | Cytoplasm | kinase |
| STK38L | serine/threonine kinase 38 like | AB023182 | 8 | Cytoplasm | kinase |
| SUB1 | SUB1 homolog (S. cerevisiae) | -- | 8 | Nucleus | transcription regulator |
| SUZ12 | suppressor of zeste 12 homolog (Drosophila) | -- | 5 | Nucleus | other |
| SYT3 | synaptotagmin III | NM_032298 | 7 | Cytoplasm | transporter |
| TBP | TATA box binding protein | -- | 4 | Nucleus | transcription regulator |
| TCF20 | transcription factor 20 (AR1) | -- | 4 | Nucleus | transcription regulator |
| TFDP2 | transcription factor Dp-2 (E2F dimerization partner 2) | NM_006286 | 5 | Nucleus | transcription regulator |
| TGFB1 | transforming growth factor, beta 1 (Camurati-Engelmann disease) | -- | 6 | Extracellular Space | growth factor |
| TGM1 | transglutaminase 1 (K polypeptide epidermal type I, protein-glutamine-gamma-glutamyltransferase) | -- | 6 | Plasma Membrane | enzyme |
| THBD | thrombomodulin | NM_000361 | 6 | Plasma Membrane | transmembrane receptor |
| THBS2 | thrombospondin 2 | -- | 1, 8 | Extracellular Space | other |
| THRA | thyroid hormone receptor, alpha (erythroblastic leukemia viral (v-erb-a) oncogene homolog, avian) | NM_003250 | 8 | Nucleus | ligand-dependent nuclear receptor |
| THRB | thyroid hormone receptor, beta (erythroblastic leukemia viral (v-erb-a) oncogene homolog 2, avian) | -- | 8 | Nucleus | ligand-dependent nuclear receptor |
| THRSP | thyroid hormone responsive (SPOT14 homolog, rat) | -- | 8 | Nucleus | other |
| TIMP2 | TIMP metallopeptidase inhibitor 2 | AL110197 | 1 | Extracellular Space | other |
| TIMP4 | TIMP metallopeptidase inhibitor 4 | -- | 1 | Extracellular Space | other |
| TMEM93 | transmembrane protein 93 | NM_031298 | 8 | Unknown | other |
| TNF | tumor necrosis factor (TNF superfamily, member 2) | -- | 3 | Extracellular Space | cytokine |
| TNFAIP2 (includes EG:7127) | tumor necrosis factor, alpha-induced protein 2 | -- | 6 | Extracellular Space | other |
| TNFRSF8 | tumor necrosis factor receptor superfamily, member 8 | NM_001243 | 3 | Plasma Membrane | transmembrane receptor |
| TNFRSF9 | tumor necrosis factor receptor superfamily, member 9 | -- | 3 | Plasma Membrane | other |
| TNFRSF6B | tumor necrosis factor receptor superfamily, member 6b, decoy | -- | 2 | Plasma Membrane | transmembrane receptor |
| TNFSF11 | tumor necrosis factor (ligand) superfamily, member 11 | -- | 3 | Extracellular Space | cytokine |
| TP53 | tumor protein p53 (Li-Fraumeni syndrome) | -- | 4, 8 | Nucleus | transcription regulator |
| TP53I3 | tumor protein p53 inducible protein 3 | -- | 4 | Unknown | enzyme |
| TRIM22 | tripartite motif-containing 22 | NM_006074 | 4 | Cytoplasm | transcription regulator |
| TSC2 | tuberous sclerosis 2 | -- | 7 | Cytoplasm | other |
| TSHB | thyroid stimulating hormone, beta | -- | 8 | Extracellular Space | other |
| TUBB3 | tubulin, beta 3 | NM_006086 | 4 | Cytoplasm | other |
| TXNRD1 | thioredoxin reductase 1 | -- | 4 | Cytoplasm | enzyme |
| TYROBP | TYRO protein tyrosine kinase binding protein | -- | 11 | Plasma Membrane | other |
| USP31 | ubiquitin specific peptidase 31 | AK057491 |  | Unknown | peptidase |
| VASP | vasodilator-stimulated phosphoprotein | NM_003370 | 6 | Plasma Membrane | other |
| VCAM1 | vascular cell adhesion molecule 1 | NM_001078 | 1 | Plasma Membrane | other |
| VIL2 | villin 2 (ezrin) | -- | 7 | Plasma Membrane | other |
| WARS | tryptophanyl-tRNA synthetase | NM_004184 | 2 | Cytoplasm | enzyme |
| YWHAH | tyrosine 3-monooxygenase/ eta polypeptide | -- | 7 | Cytoplasm | transcription regulator |
| ZNF148 | zinc finger protein 148 (pHZ-52) | -- | 4 | Nucleus | transcription regulator |
