# Supplementary material for: Temporal dynamics of gene expression in the lung in a baboon model of E. coli sepsis
Source: BMC Genomics. 2007 Feb 26;8:58. doi: 10.1186/1471-2164-8-58 (PMC1819384; doi:10.1186/1471-2164-8-58)
Supplement: Additional file 4 — Table_A4. The table lists the genes of the IPA networks at the time point 24 hrs. [file 1471-2164-8-58-S4.doc]

| ***Table A4:* Genes included in the IPA networks at 24 hrs** | | | | | |
| --- | --- | --- | --- | --- | --- |
| ***Name*** | ***Description*** | ***Genbank*** | ***Ntw*** | ***Location*** | ***Family*** |
| ADAMTS4 | ADAM metallopeptidase with thrombospondin type 1 motif, 4 | -- | 1 | Extracellular Space | peptidase |
| AFP | alpha-fetoprotein | -- | 2, 3 | Extracellular Space | transporter |
| AGT | angiotensinogen (serpin peptidase inhibitor, clade A, member 8) | -- | 3 | Extracellular Space | other |
| AHR | aryl hydrocarbon receptor | -- | 2 | Nucleus | ligand-dependent nuclear receptor |
| AMD1 | adenosylmethionine decarboxylase 1 | -- | 1 | Unknown | enzyme |
| APEX1 | APEX nuclease (multifunctional DNA repair enzyme) 1 | -- | 3 | Nucleus | enzyme |
| APOE | apolipoprotein E | NM_000041 | 1 | Extracellular Space | transporter |
| Beta-Hexosaminidase | -- | -- | 6 | Cytoplasm | other |
| BLZF1 | basic leucine zipper nuclear factor 1 (JEM-1) | -- | 13 | Nucleus | transcription regulator |
| BMX | BMX non-receptor tyrosine kinase | NM_001721 | 2 | Cytoplasm | kinase |
| BRD2 | bromodomain containing 2 | -- | 3 | Nucleus | kinase |
| C3 | complement component 3 | -- | 4 | Extracellular Space | peptidase |
| C7 | complement component 7 | NM_000587 | 2 | Unknown | other |
| C5B6 | -- | -- | 2 | Unknown | other |
| CCL1 | chemokine (C-C motif) ligand 1 | -- | 1 | Extracellular Space | cytokine |
| CCNI | cyclin I | NM_006835 | 2 | Unknown | other |
| CD59 | CD59 molecule, complement regulatory protein | NM_000611 | 2 | Plasma Membrane | other |
| CD74 | CD74 molecule, major histocompatibility complex, class II invariant chain | -- | 4 | Plasma Membrane | transmembrane receptor |
| CD81 | CD81 molecule | -- | 4 | Plasma Membrane | other |
| CD160 | CD160 molecule | NM_007053 | 1 | Plasma Membrane | transmembrane receptor |
| CDK8 | cyclin-dependent kinase 8 | NM_001260 | 3 | Nucleus | kinase |
| CDKN2A | cyclin-dependent kinase inhibitor 2A (melanoma, p16, inhibits CDK4) | -- | 2 | Nucleus | transcription regulator |
| CEACAM1 | -- | -- | 4 | Plasma Membrane | transmembrane receptor |
| CENTD1 | centaurin, delta 1 | NM_015230 | 4 | Unknown | other |
| COL6A3 | collagen, type VI, alpha 3 | NM_004369 | 3 | Extracellular Space | other |
| COX5B | cytochrome c oxidase subunit Vb | -- | 3 | Cytoplasm | enzyme |
| COX6C | cytochrome c oxidase subunit VIc | -- | 3 | Cytoplasm | enzyme |
| COX7A1 | cytochrome c oxidase subunit VIIa polypeptide 1 (muscle) | -- | 3 | Cytoplasm | enzyme |
| COX7C | cytochrome c oxidase subunit VIIc | NM_001867 | 3 | Cytoplasm | enzyme |
| CPB2 | carboxypeptidase B2 (plasma, carboxypeptidase U) | -- | 2 | Extracellular Space | peptidase |
| CTLA4 | cytotoxic T-lymphocyte-associated protein 4 | -- | 4 | Plasma Membrane | other |
| CXCL3 | chemokine (C-X-C motif) ligand 3 | -- | 2 | Extracellular Space | cytokine |
| CXCL9 | chemokine (C-X-C motif) ligand 9 | -- | 4 | Extracellular Space | cytokine |
| CYP1A2 | cytochrome P450, family 1, subfamily A, polypeptide 2 | NM_000761 | 2 | Cytoplasm | enzyme |
| DAP | death-associated protein | NM_004394 |  | Cytoplasm | other |
| EEF2 | eukaryotic translation elongation factor 2 | NM_001961 | 1, 3 | Cytoplasm | translation regulator |
| EIF3S10 | eukaryotic translation initiation factor 3, subunit 10 theta, 150/170kDa | -- | 3 | Cytoplasm | translation regulator |
| EIF3S6IP | eukaryotic translation initiation factor 3, subunit 6 interacting protein | NM_016091 | 3 | Cytoplasm | other |
| EIF4A1 | eukaryotic translation initiation factor 4A, isoform 1 | -- | 3 | Cytoplasm | translation regulator |
| EIF4A2 | eukaryotic translation initiation factor 4A, isoform 2 | NM_001967 | 3 | Cytoplasm | translation regulator |
| EIF4B | eukaryotic translation initiation factor 4B | -- | 3 | Cytoplasm | translation regulator |
| EIF4E | eukaryotic translation initiation factor 4E | -- | 3 | Cytoplasm | translation regulator |
| EIF4G1 | eukaryotic translation initiation factor 4 gamma, 1 | -- | 3 | Cytoplasm | translation regulator |
| EIF4G2 | eukaryotic translation initiation factor 4 gamma, 2 | NM_001418 | 3 | Cytoplasm | translation regulator |
| EIF4G3 | eukaryotic translation initiation factor 4 gamma, 3 | -- | 3 | Cytoplasm | translation regulator |
| EMP2 | epithelial membrane protein 2 | NM_001424 | 8 | Plasma Membrane | other |
| EP300 | E1A binding protein p300 | -- | 2 | Nucleus | transcription regulator |
| EPN1 | epsin 1 | -- | 11 | Plasma Membrane | other |
| EPS15 | epidermal growth factor receptor pathway substrate 15 | -- | 11 | Plasma Membrane | other |
| F2 | coagulation factor II (thrombin) | -- | 2 | Extracellular Space | peptidase |
| FGF23 | fibroblast growth factor 23 | NM_020638 | 7 | Extracellular Space | growth factor |
| FMO2 | flavin containing monooxygenase 2 | NM_001460 | 5 | Cytoplasm | enzyme |
| FN1 | fibronectin 1 | NM_002026 | 1 | Plasma Membrane | enzyme |
| GBP2 | guanylate binding protein 2, interferon-inducible | NM_004120 | 1, 2 | Cytoplasm | enzyme |
| GCNT1 | glucosaminyl (N-acetyl) transferase 1, core 2 (beta-1,6-N-acetylglucosaminyltransferase) | -- | 2 | Cytoplasm | enzyme |
| GOLGA2 | golgi autoantigen, golgin subfamily a, 2 | -- | 13 | Cytoplasm | other |
| GORASP2 | golgi reassembly stacking protein 2, 55kDa | -- | 13 | Cytoplasm | other |
| GPX1 | glutathione peroxidase 1 | NM_000581 | 1 | Cytoplasm | enzyme |
| GREM1 | gremlin 1, cysteine knot superfamily, homolog (Xenopus laevis) | -- | 12 | Extracellular Space | other |
| GTSE1 | G-2 and S-phase expressed 1 | NM_016426 | 2 | Cytoplasm | other |
| GYPA | glycophorin A (MNS blood group) | NM_002099 | 4 | Plasma Membrane | other |
| GZMB | granzyme B (granzyme 2, cytotoxic T-lymphocyte-associated serine esterase 1) | -- | 4 | Cytoplasm | peptidase |
| H2-Eb2 | histocompatibility 2, class II antigen E beta2 | -- | 4 | Unknown | other |
| H3F3B | H3 histone, family 3B (H3.3B) | AF218029 | 3 | Nucleus | other |
| HGF | hepatocyte growth factor (hepapoietin A; scatter factor) | -- | 10 | Extracellular Space | growth factor |
| HLA-DMA | major histocompatibility complex, class II, DM alpha | -- | 4 | Plasma Membrane | transmembrane receptor |
| HLA-DMB | major histocompatibility complex, class II, DM beta | -- | 4 | Plasma Membrane | transmembrane receptor |
| HLA-DOA | major histocompatibility complex, class II, DO alpha | -- | 4 | Plasma Membrane | transmembrane receptor |
| HLA-DOB | major histocompatibility complex, class II, DO beta | -- | 4 | Plasma Membrane | transmembrane receptor |
| HLA-DQA1 | major histocompatibility complex, class II, DQ alpha 1 | -- | 4 | Plasma Membrane | transmembrane receptor |
| HLA-DQB1 | major histocompatibility complex, class II, DQ beta 1 | -- | 4 | Plasma Membrane | transmembrane receptor |
| HLA-DRA | major histocompatibility complex, class II, DR alpha | -- | 4 | Plasma Membrane | transmembrane receptor |
| HLA-DRB1 | major histocompatibility complex, class II, DR beta 1 | -- | 4 | Plasma Membrane | transmembrane receptor |
| HLA-DRB5 | major histocompatibility complex, class II, DR beta 5 | NM_002125 | 4 | Plasma Membrane | transmembrane receptor |
| HNRPC | heterogeneous nuclear ribonucleoprotein C (C1/C2) | NM_031314 | 1 | Nucleus | other |
| HPSE | heparanase | AF155510 | 2 | Plasma Membrane | enzyme |
| HTATIP2 | HIV-1 Tat interactive protein 2, 30kDa | -- | 1 | Nucleus | transcription regulator |
| ICAM1 | intercellular adhesion molecule 1 (CD54), human rhinovirus receptor | -- | 2 | Plasma Membrane | transmembrane receptor |
| IFNG | interferon, gamma | -- | 4 | Extracellular Space | cytokine |
| IGFBP7 | insulin-like growth factor binding protein 7 | NM_001553 | 1 | Extracellular Space | transporter |
| IL2 | interleukin 2 | -- | 4 | Extracellular Space | cytokine |
| IL6 | interleukin 6 (interferon, beta 2) | -- | 2 | Extracellular Space | cytokine |
| IL21 | interleukin 21 | -- | 4 | Extracellular Space | cytokine |
| IL12RB1 | interleukin 12 receptor, beta 1 | -- | 4 | Plasma Membrane | transmembrane receptor |
| IL12RB2 | interleukin 12 receptor, beta 2 | -- | 4 | Plasma Membrane | transmembrane receptor |
| IL18BP | interleukin 18 binding protein | -- | 4 | Extracellular Space | other |
| IL2RG | interleukin 2 receptor, gamma (severe combined immunodeficiency) | -- | 4 | Plasma Membrane | transmembrane receptor |
| JUN | v-jun sarcoma virus 17 oncogene homolog (avian) | NM_002228 | 1 | Nucleus | transcription regulator |
| KIF20A | kinesin family member 20A | -- | 2 | Cytoplasm | transporter |
| KIR2DL4 | killer cell immunoglobulin-like receptor, two domains, long cytoplasmic tail, 4 | -- | 4 | Plasma Membrane | transmembrane receptor |
| LY86 | lymphocyte antigen 86 | NM_004271 | 2 | Plasma Membrane | other |
| MAP3K3 | mitogen-activated protein kinase kinase kinase 3 | NM_002401 | 1 | Cytoplasm | kinase |
| MAP3K8 | mitogen-activated protein kinase kinase kinase 8 | -- | 4 | Cytoplasm | kinase |
| MAP3K13 | mitogen-activated protein kinase kinase kinase 13 | -- | 1 | Cytoplasm | kinase |
| MAPK8IP1 | mitogen-activated protein kinase 8 interacting protein 1 | NM_005456 | 1 | Cytoplasm | other |
| MGAT2 | mannosyl (alpha-1,6-)-glycoprotein beta-1,2-N-acetylglucosaminyltransferase | NM_002408 |  | Cytoplasm | enzyme |
| MGP | matrix Gla protein | NM_000900 | 2 | Extracellular Space | other |
| MKNK1 | MAP kinase interacting serine/threonine kinase 1 | -- | 3 | Cytoplasm | kinase |
| MMP2 | matrix metallopeptidase 2 (gelatinase A, 72kDa gelatinase, 72kDa type IV collagenase) | NM_004530 | 1 | Extracellular Space | peptidase |
| MMP10 | matrix metallopeptidase 10 (stromelysin 2) | -- | 2 | Extracellular Space | peptidase |
| MMP16 | matrix metallopeptidase 16 (membrane-inserted) | -- | 1 | Extracellular Space | peptidase |
| MMP25 | matrix metallopeptidase 25 | -- | 1 | Extracellular Space | peptidase |
| MMP26 | matrix metallopeptidase 26 | -- | 1 | Extracellular Space | peptidase |
| MYC | v-myc myelocytomatosis viral oncogene homolog (avian) | -- | 3 | Nucleus | transcription regulator |
| NBL1 | neuroblastoma, suppression of tumorigenicity 1 | -- | 12 | Nucleus | other |
| NMB | neuromedin B | NM_021077 | 1 | Extracellular Space | other |
| NME2 | non-metastatic cells 2, protein (NM23B) expressed in | -- | 1 | Nucleus | kinase |
| NSEP1 | Y box binding protein 1 | -- | 4 | Nucleus | transcription regulator |
| P2RX7 | purinergic receptor P2X, ligand-gated ion channel, 7 | -- | 8 | Plasma Membrane | ion channel |
| PEG3 | paternally expressed 3 | -- | 3 | Nucleus | kinase |
| PFDN5 | prefoldin subunit 5 | NM_002624 | 3 | Nucleus | transcription regulator |
| PHEX | phosphate regulating endopeptidase homolog, X-linked (hypophosphatemia, vitamin D resistant rickets) | -- | 7 | Cytoplasm | peptidase |
| PIM2 | pim-2 oncogene | -- | 4 | Unknown | kinase |
| PLA2G1B | phospholipase A2, group IB (pancreas) | -- | 1 | Extracellular Space | enzyme |
| PLSCR2 | phospholipid scramblase 2 | NM_020359 | 6 | Nucleus | enzyme |
| PTN | pleiotrophin (heparin binding growth factor 8, neurite growth-promoting factor 1) | -- | 3 | Extracellular Space | growth factor |
| RAB2 | RAB2, member RAS oncogene family | NM_002865 | 13 | Cytoplasm | enzyme |
| RAB36 | RAB36, member RAS oncogene family | NM_004914 | 3 | Cytoplasm | enzyme |
| RAB6A | RAB6A, member RAS oncogene family | AK057157 | 2 | Cytoplasm | enzyme |
| RALBP1 | ralA binding protein 1 | -- | 11 | Cytoplasm | other |
| RECK | reversion-inducing-cysteine-rich protein with kazal motifs | -- | 1 | Plasma Membrane | other |
| REPS1 | RALBP1 associated Eps domain containing 1 | NM_031922 | 11 | Unknown | other |
| RGS8 | regulator of G-protein signalling 8 | AK057753 | 9 | Unknown | other |
| ROBO1 | roundabout, axon guidance receptor, homolog 1 (Drosophila) | -- | 12 | Plasma Membrane | transmembrane receptor |
| S100A8 | S100 calcium binding protein A8 (calgranulin A) | NM_002964 | 4 | Cytoplasm | other |
| S100B | S100 calcium binding protein, beta (neural) | -- | 3 | Cytoplasm | other |
| SCARB1 | scavenger receptor class B, member 1 | -- | 4 | Plasma Membrane | transporter |
| SLC7A1 | solute carrier family 7 (cationic amino acid transporter, y+ system), member 1 | -- | 2 | Plasma Membrane | transporter |
| SLCO2A1 | solute carrier organic anion transporter family, member 2A1 | NM_005630 |  | Plasma Membrane | transporter |
| SLIT1 | slit homolog 1 (Drosophila) | AB011537 | 12 | Extracellular Space | other |
| SMARCA4 | SWI/SNF related, matrix associated, actin dependent regulator of chromatin, subfamily a, member 4 | -- | 5 | Nucleus | transcription regulator |
| SMARCB1 | SWI/SNF related, matrix associated, actin dependent regulator of chromatin, subfamily b, member 1 | -- | 3 | Nucleus | other |
| SMC1L2 | SMC1 structural maintenance of chromosomes 1-like 2 (yeast) | -- | 2 | Nucleus | transporter |
| SMTN | smoothelin | -- | 2 | Extracellular Space | other |
| SOAT1 | sterol O-acyltransferase (acyl-Coenzyme A: cholesterol acyltransferase) 1 | -- | 4 | Cytoplasm | enzyme |
| SOD2 | superoxide dismutase 2, mitochondrial | NM_000636 | 1 | Cytoplasm | enzyme |
| SPARC | secreted protein, acidic, cysteine-rich (osteonectin) | NM_003118 | 1, 3 | Extracellular Space | other |
| STAG2 | stromal antigen 2 | NM_006603 | 2 | Nucleus | other |
| STAT4 | signal transducer and activator of transcription 4 | NM_003151 | 1, 4 | Nucleus | transcription regulator |
| TCF12 | transcription factor 12 (HTF4, helix-loop-helix transcription factors 4) | NM_003205 | 2 | Nucleus | transcription regulator |
| TCOF1 | Treacher Collins-Franceschetti syndrome 1 | -- | 10 | Nucleus | transporter |
| TFE3 | transcription factor binding to IGHM enhancer 3 | NM_006521 | 2 | Nucleus | transcription regulator |
| TGFB1 | transforming growth factor, beta 1 (Camurati-Engelmann disease) | -- | 2 | Extracellular Space | growth factor |
| TGIF | TGFB-induced factor (TALE family homeobox) | -- | 2 | Nucleus | transcription regulator |
| THBD | thrombomodulin | NM_000361 | 1, 2 | Plasma Membrane | transmembrane receptor |
| THBS2 | thrombospondin 2 | -- | 3 | Extracellular Space | other |
| TIMP2 | TIMP metallopeptidase inhibitor 2 | AL110197 | 1 | Extracellular Space | other |
| TIMP3 | TIMP metallopeptidase inhibitor 3 (Sorsby fundus dystrophy, pseudoinflammatory) | NM_000362 | 1 | Extracellular Space | other |
| TIMP4 | TIMP metallopeptidase inhibitor 4 | -- | 1 | Extracellular Space | other |
| TMSB4X | thymosin, beta 4, X-linked | AK055976 | 3 | Cytoplasm | other |
| TNF | tumor necrosis factor (TNF superfamily, member 2) | -- | 2 | Extracellular Space | cytokine |
| TNFRSF8 | tumor necrosis factor receptor superfamily, member 8 | NM_001243 | 1 | Plasma Membrane | transmembrane receptor |
| TNFRSF6B | tumor necrosis factor receptor superfamily, member 6b, decoy | -- | 4 | Plasma Membrane | transmembrane receptor |
| TP53 | tumor protein p53 (Li-Fraumeni syndrome) | -- | 3 | Nucleus | transcription regulator |
| TSC22D3 | TSC22 domain family, member 3 | AB025432 | 1 | Nucleus | transcription regulator |
| TUBB3 | tubulin, beta 3 | NM_006086 | 3 | Cytoplasm | other |
| TUBB4 | tubulin, beta 4 | -- | 3 | Cytoplasm | other |
| TYR | tyrosinase (oculocutaneous albinism IA) | -- | 2 | Cytoplasm | enzyme |
| VCL | vinculin | NM_014000 | 1 | Plasma Membrane | enzyme |
| VDP | vesicle docking protein p115 | -- | 13 | Cytoplasm | transporter |
| VEGF | vascular endothelial growth factor | AF022375 | 1 | Extracellular Space | growth factor |
| YWHAB | tyrosine 3-monooxygenase/tryptophan 5-monooxygenase activation protein, beta polypeptide | -- | 9 | Cytoplasm | other |
| ZNF9 | zinc finger protein 9 | NM_003418 | 10 | Nucleus | transcription regulator |
