# Supplementary material for: Temporal dynamics of gene expression in the lung in a baboon model of E. coli sepsis
Source: BMC Genomics. 2007 Feb 26;8:58. doi: 10.1186/1471-2164-8-58 (PMC1819384; doi:10.1186/1471-2164-8-58)
Supplement: Additional file 1 — Table_A1. The table lists the genes presented in the cluster analysis. [file 1471-2164-8-58-S1.doc]

***Table A1:*** List of differentially expressed genes grouped in clusters

| Cluster | | Gene Bank ID | | Gene Symbol | | Gene Name |
| --- | --- | --- | --- | --- | --- | --- |
| 1 | | NM_001305 | | CLDN4 | | claudin 4 |
| 1 | | NM_001271 | | CHD2 | | chromodomain helicase DNA binding protein 2 |
| 1 | | NM_002276 | | KRT19 | | keratin 19 |
| 1 | | AX014270 | |  | |  |
| 1 | | NM_012317 | | LDOC1 | | leucine zipper, down-regulated in cancer 1 |
| 1 | | NM_006286 | | TFDP2 | | transcription factor Dp-2 (E2F dimerization partner 2) |
| 1 | | AL161622 | |  | |  |
| 1 | | AF113701 | | RPL22 | | ribosomal protein L22 |
| 1 | | NM_005708 | | GPC6 | | glypican 6 |
| 2 | | AK021481 | |  | | CDNA FLJ11419 fis, clone HEMBA1000985 |
| 2 | | AK000144 | |  | | CDNA FLJ20137 fis, clone COL07137 |
| 2 | | NM_001290 | | LDB2 | | LIM domain binding 2 |
| 2 | | NM_005854 | | RAMP2 | | receptor (calcitonin) activity modifying protein 2 |
| 2 | | NM_001424 | | EMP2 | | epithelial membrane protein 2 |
| 2 | | BC009353 | |  | |  |
| 2 | | NM_002766 | | PRPSAP1 | | phosphoribosyl pyrophosphate synthetase-associated protein 1 |
| 2 | | NM_000361 | | THBD | | thrombomodulin |
| 2 | | AF074331 | |  | |  |
| 2 | | NM_002165 | | ID1 | | inhibitor of DNA binding 1, dominant negative helix-loop-helix protein |
| 2 | | NM_000900 | | MGP | | matrix Gla protein |
| 2 | | AK057328 | | TNS | | tensin |
| 2 | | AB018301 | | GPR116 | | G protein-coupled receptor 116 |
| 2 | | NM_004684 | | SPARCL1 | | SPARC-like 1 (mast9, hevin) |
| 2 | | AL050364 | | PKP4 | | plakophilin 4 |
| 2 | | NM_002532 | | NUP88 | | nucleoporin 88kDa |
| 2 | | AF334710 | | PDK4 | | pyruvate dehydrogenase kinase, isoenzyme 4 |
| 2 | | NM_001946 | | DUSP6 | | dual specificity phosphatase 6 |
| 2 | | BC010635 | |  | | Clone IMAGE:3867347, mRNA, partial cds |
| 2 | | AB025432 | | DSIPI | | delta sleep inducing peptide, immunoreactor |
| 2 | | NM_000014 | | A2M | | alpha-2-macroglobulin |
| 2 | | NM_004530 | | MMP2 | | matrix metalloproteinase 2 (gelatinase A, 72kDa gelatinase,  72kDa type IV collagenase) |
| 2 | | NM_001901 | | CTGF | | connective tissue growth factor |
| 2 | | NM_016250 | | NDRG2 | | NDRG family member 2 |
| 2 | | AY027862 | | UCC1 | | upregulated in colorectal cancer gene 1 |
| 2 | | NM_006407 | | JWA | | cytoskeleton related vitamin A responsive protein |
| 2 | | NM_001460 | | FMO2 | | flavin containing monooxygenase 2 |
| 2 | | NM_001753 | | CAV1 | | caveolin 1, caveolae protein, 22kDa |
| 2 | | AL136693 | | CYBRD1 | | cytochrome b reductase 1 |
| 2 | | NM_003118 | | SPARC | | secreted protein, acidic, cysteine-rich (osteonectin) |
| 2 | | NM_001444 | | FABP5 | | fatty acid binding protein 5 (psoriasis-associated) |
| 2 | | AF368463 | | CPM | | carboxypeptidase M |
| 2 | | NM_024567 | | FLJ21616 | | hypothetical protein FLJ21616 |
| 2 | | M61906 | | PIK3R1 | | phosphoinositide-3-kinase, regulatory subunit, polypeptide 1 (p85 alpha) |
| 3 | | NM_001243 | | TNFRSF8 | | tumor necrosis factor receptor superfamily, member 8 |
| 3 | | NM_005610 | | RBBP4 | | retinoblastoma binding protein 4 |
| 3 | | NM_005944 | | MOX2 | | antigen identified by monoclonal antibody MRC OX-2 |
| 3 | | NM_006729 | | DIAPH2 | | diaphanous homolog 2 (Drosophila) |
| 3 | | NM_000851 | | GSTM5 | | glutathione S-transferase M5 |
| 3 | | NM_015994 | | ATP6V1D | | ATPase, H+ transporting, lysosomal 34kDa, V1 subunit D |
| 3 | | AK024944 | |  | | MRNA* cDNA DKFZp434N185 (from clone DKFZp434N185) |
| 3 | | AK000757 | | SORT1 | | sortilin 1 |
| 3 | | NM_001961 | | EEF2 | | eukaryotic translation elongation factor 2 |
| 3 | | AB033097 | | KIAA1271 | | KIAA1271 protein |
| 3 | | NM_006791 | | MORF4L1 | | mortality factor 4 like 1 |
| 3 | | AL136883 | | PHTF2 | | putative homeodomain transcription factor 2 |
| 3 | | AK021584 | |  | | CDNA FLJ11522 fis, clone HEMBA1002498 |
| 3 | | NM_006185 | | NUMA1 | | nuclear mitotic apparatus protein 1 |
| 3 | | AK056404 | | FLJ31842 | | hypothetical protein FLJ31842 |
| 3 | | NM_006103 | | WFDC2 | | WAP four-disulfide core domain 2 |
| 3 | | NM_000581 | | GPX1 | | glutathione peroxidase 1 |
| 3 | | NM_002624 | | PFDN5 | | prefoldin 5 |
| 3 | | AK057157 | | RAB6A | | RAB6A, member RAS oncogene family |
| 3 | | AB058726 | | PHF6 | | PHD finger protein 6 |
| 3 | | AK056295 | | DKFZp547D2210 | | hypothetical protein DKFZp547D2210 |
| 3 | | NM_002865 | | RAB2 | | RAB2, member RAS oncogene family |
| 3 | | NM_025032 | |  | |  |
| 3 | | AK056408 | |  | |  |
| 3 | | AF268193 | | IRA1 | | likely ortholog of mouse IRA1 protein |
| 3 | | AL133574 | | TEAD1 | | TEA domain family member 1 (SV40 transcriptional enhancer factor) |
| 3 | | NM_031492 | | MGC10871 | | hypothetical protein similar to RNA-binding protein lark |
| 3 | | AF022375 | | VEGF | | vascular endothelial growth factor |
| 3 | | AK022168 | |  | | CDNA FLJ12106 fis, clone HEMBB1002702 |
| 3 | | NM_004914 | | RAB36 | | RAB36, member RAS oncogene family |
| 3 | | NM_032298 | | SYT3 | | synaptotagmin III |
| 3 | | NM_006600 | | NUDC | | nuclear distribution gene C homolog (A. nidulans) |
| 3 | | NM_004926 | | ZFP36L1 | | zinc finger protein 36, C3H type-like 1 |
| 3 | | NM_001980 | | EPIM | | epimorphin |
| 3 | | NM_006835 | | CCNI | | cyclin I |
| 3 | | NM_013994 | | DDR1 | | discoidin domain receptor family, member 1 |
| 3 | | NM_001769 | | CD9 | | CD9 antigen (p24) |
| 3 | | NM_000717 | | CA4 | | carbonic anhydrase IV |
| 3 | | NM_002994 | | CXCL5 | | chemokine (C-X-C motif) ligand 5 |
| 3 | | NM_001848 | | COL6A1 | | collagen, type VI, alpha 1 |
| 3 | | NM_005778 | | RBM5 | | RNA binding motif protein 5 |
| 3 | | AK023675 | | UTRN | | utrophin (homologous to dystrophin) |
| 3 | | AK056229 | |  | | CDNA FLJ31667 fis, clone NT2RI2004840 |
| 3 | | NM_013384 | | LASS2 | | LAG1 longevity assurance homolog 2 (S. cerevisiae) |
| 3 | | NM_014713 | | LAPTM4A | | lysosomal-associated protein transmembrane 4 alpha |
| 3 | | NM_002909 | | REG1A | | regenerating islet-derived 1 alpha (pancreatic stone protein,  pancreatic thread protein) |
| 3 | | NM_000611 | | CD59 | | CD59 antigen |
| 3 | | NM_004333 | | BRAF | | v-raf murine sarcoma viral oncogene homolog B1 |
| 3 | | AB040930 | | LRRN1 | | leucine rich repeat neuronal 1 |
| 3 | | NM_003151 | | STAT4 | | signal transducer and activator of transcription 4 |
| 3 | | NM_017661 | |  | |  |
| 3 | | AK001074 | |  | | CDNA FLJ10212 fis, clone HEMBA1006467 |
| 3 | | NM_001493 | | GDI1 | | GDP dissociation inhibitor 1 |
| 3 | | AK054986 | |  | | CDNA FLJ30424 fis, clone BRACE2008881,  weakly similar to ZINC FINGER PROTEIN 195 |
| 3 | | NM_053005 | | HCCA2 | | HCCA2 protein |
| 3 | | AJ420430 | | GTF3C4 | | general transcription factor IIIC, polypeptide 4, 90kDa |
| 3 | | AK057095 | | CRR9 | | cisplatin resistance related protein CRR9p |
| 3 | | NM_001867 | | COX7C | | cytochrome c oxidase subunit VIIc |
| 3 | | AK055302 | |  | | CDNA FLJ30740 fis, clone FEBRA2000319 |
| 3 | | M28825 | | CD1A | | CD1A antigen, a polypeptide |
| 3 | | NM_031305 | | DKFZP564B1162 | | hypothetical protein DKFZp564B1162 |
| 3 | | AF285120 | |  | |  |
| 3 | | AK057299 | | DLAT | | dihydrolipoamide S-acetyltransferase  (E2 component of pyruvate dehydrogenase complex) |
| 3 | | AK027071 | | TSC22 | | transforming growth factor beta-stimulated protein TSC-22 |
| 3 | NM_014762 | | DHCR24 | | 24-dehydrocholesterol reductase | |
| 3 | | NM_002938 | | RNF4 | | ring finger protein 4 |
| 3 | | AB023182 | | STK38L | | serine/threonine kinase 38 like |
| 3 | | NM_016029 | | DHRS7 | | dehydrogenase/reductase (SDR family) member 7 |
| 3 | | NM_021194 | | SLC30A1 | | solute carrier family 30 (zinc transporter), member 1 |
| 3 | | NM_003924 | | PHOX2B | | paired-like homeobox 2b |
| 3 | | NM_031462 | | CD99L2 | | CD99 antigen-like 2 |
| 3 | | NM_000883 | | IMPDH1 | | IMP (inosine monophosphate) dehydrogenase 1 |
| 3 | | BC007901 | | LOC91461 | | hypothetical protein BC007901 |
| 3 | | NM_000587 | | C7 | | complement component 7 |
| 3 | | AK056665 | |  | |  |
| 3 | | NM_001150 | | ANPEP | | alanyl (membrane) aminopeptidase (aminopeptidase N,  aminopeptidase M, microsomal aminopeptidase, CD13, p150) |
| 3 | | AL137575 | |  | | MRNA* cDNA DKFZp564C1964 (from clone DKFZp564C1964) |
| 3 | | NM_020041 | | SLC2A9 | | solute carrier family 2 (facilitated glucose transporter), member 9 |
| 3 | | NM_001967 | | EIF4A2 | | eukaryotic translation initiation factor 4A, isoform 2 |
| 3 | | AL110152 | | CD109 | | CD109 antigen (Gov platelet alloantigens) |
| 3 | | NM_000362 | | TIMP3 | | tissue inhibitor of metalloproteinase 3 |
| 3 | | AK057575 | | SLC25A3 | | solute carrier family 25 (mitochondrial carrier* phosphate carrier), member 3 |
| 3 | | AB007869 | | KIAA0409 | | KIAA0409 protein |
| 3 | | NM_024311 | | ET | | hypothetical protein ET |
| 3 | | NM_022088 | | ZFP64 | | zinc finger protein 64 homolog (mouse) |
| 3 | | AL049313 | |  | | MRNA* cDNA DKFZp564B076 (from clone DKFZp564B076) |
| 3 | | AL136762 | | MGC15419 | | MGC15419 protein |
| 3 | | AB007857 | | RUTBC1 | | RUN and TBC1 domain containing 1 |
| 3 | | NM_001117 | | ADCYAP1 | | adenylate cyclase activating polypeptide 1 (pituitary) |
| 3 | | NM_004763 | | ITGB1BP1 | | integrin beta 1 binding protein 1 |
| 3 | | AK022418 | | MGC39821 | | hypothetical protein MGC39821 |
| 3 | | NM_032927 | | MGC13159 | | hypothetical protein MGC13159 |
| 3 | | AK001889 | | PRLR | | prolactin receptor |
| 3 | | AL110197 | | TIMP2 | | tissue inhibitor of metalloproteinase 2 |
| 3 | | D49835 | | RREB1 | | ras responsive element binding protein 1 |
| 3 | | NM_014000 | | VCL | | vinculin |
| 3 | | NM_014042 | | DKFZP564M082 | | DKFZP564M082 protein |
| 3 | | NM_002164 | | INDO | | indoleamine-pyrrole 2,3 dioxygenase |
| 3 | | NM_016524 | | LOC51760 | | B/K protein |
| 3 | | AF208850 | | PTP4A2 | | protein tyrosine phosphatase type IVA, member 2 |
| 3 | | AK021632 | | LOC91526 | | hypothetical protein DKFZp434D2328 |
| 3 | | BC011405 | | LOC112476 | | similar to lymphocyte antigen 6 complex,  locus G5B* G5b protein* open reading frame 31 |
| 3 | | NM_002026 | | FN1 | | fibronectin 1 |
| 3 | | NM_019095 | | C20orf155 | | chromosome 20 open reading frame 155 |
| 3 | | NM_016091 | | EIF3S6IP | | eukaryotic translation initiation factor 3, subunit 6 interacting protein |
| 3 | | NM_003205 | | TCF12 | | transcription factor 12 (HTF4, helix-loop-helix transcription factors 4) |
| 3 | | NM_004369 | | COL6A3 | | collagen, type VI, alpha 3 |
| 3 | | AL049397 | | PNAS-4 | | CGI-146 protein |
| 3 | | AK000951 | |  | | CDNA FLJ10089 fis, clone HEMBA1002237 |
| 3 | | NM_021003 | | PPM1A | | protein phosphatase 1A (formerly 2C),  magnesium-dependent, alpha isoform |
| 3 | | BC016993 | | LOC147645 | | hypothetical protein LOC147645 |
| 3 | | NM_001431 | | EPB41L2 | | erythrocyte membrane protein band 4.1-like 2 |
| 3 | | AK022090 | |  | | Clone IMAGE:5274212, mRNA |
| 3 | | NM_012134 | | LMOD1 | | leiomodin 1 (smooth muscle) |
| 3 | | AB029316 | | RNF19 | | ring finger protein 19 |
| 3 | | AK055914 | | GNG12 | | guanine nucleotide binding protein (G protein), gamma 12 |
| 3 | | NM_004394 | | DAP | | death-associated protein |
| 3 | | NM_015642 | | ZNF288 | | zinc finger protein 288 |
| 4 | | AL049265 | |  | | MRNA* cDNA DKFZp564F053 (from clone DKFZp564F053) |
| 4 | | BC004215 | |  | |  |
| 4 | | NM_005129 | |  | |  |
| 4 | | AJ224082 | |  | |  |
| 4 | | AB032969 | | KIAA1143 | | KIAA1143 protein |
| 4 | | NM_000971 | | RPL7 | | ribosomal protein L7 |
| 4 | | NM_002228 | | JUN | | v-jun sarcoma virus 17 oncogene homolog (avian) |
| 4 | | NM_031243 | | HNRPA2B1 | | heterogeneous nuclear ribonucleoprotein A2/B1 |
| 4 | | AK055976 | | TMSB4X | | thymosin, beta 4, X-linked |
| 4 | | AK023362 | |  | |  |
| 4 | | NM_002125 | | HLA-DRB3 | | major histocompatibility complex, class II, DR beta 3 |
| 4 | | NM_001010 | | RPS6 | | ribosomal protein S6 |
| 4 | | NM_007013 | | WWP1 | | WW domain-containing protein 1 |
| 4 | | NM_003418 | | ZNF9 | | zinc finger protein 9 (a cellular retroviral nucleic acid binding protein) |
| 4 | | NM_001553 | | IGFBP7 | | insulin-like growth factor binding protein 7 |
| 4 | | NM_000041 | | APOE | | apolipoprotein E |
| 4 | | AL035604 | |  | |  |
| 4 | | NM_001967 | | EIF4A2 | | eukaryotic translation initiation factor 4A, isoform 2 |
| 4 | | NM_001282 | | AP2B1 | | adaptor-related protein complex 2, beta 1 subunit |
| 4 | | NM_005630 | | SLCO2A1 | | solute carrier organic anion transporter family, member 2A1 |
| 4 | | NM_018049 | | PLEKHJ1 | | pleckstrin homology domain containing, family J member 1 |
| 4 | | NM_004872 | | C1orf8 | | chromosome 1 open reading frame 8 |
| 4 | | AF218029 | | H3F3B | | H3 histone, family 3B (H3.3B) |
| 4 | | AK027539 | | LOC201725 | | hypothetical protein LOC201725 |
| 4 | | NM_003753 | | EIF3S7 | | eukaryotic translation initiation factor 3, subunit 7 zeta, 66/67kDa |
| 5 | | NM_014059 | | RGC32 | | response gene to complement 32 |
| 5 | | NM_006074 | | TRIM22 | | tripartite motif-containing 22 |
| 5 | | NM_015658 | | DKFZP564C186 | | DKFZP564C186 protein |
| 5 | | NM_003922 | | HERC1 | | hect (homologous to the E6-AP (UBE3A) carboxyl terminus)  domain and RCC1 (CHC1)-like domain (RLD) 1 |
| 5 | | NM_012451 | | SYNGR4 | | synaptogyrin 4 |
| 5 | | AK055913 | |  | | CDNA FLJ31351 fis, clone MESAN2000167 |
| 5 | | NM_004184 | | WARS | | tryptophanyl-tRNA synthetase |
| 5 | | BC011916 | | LOC112817 | | hypothetical protein BC011916 |
| 5 | | BF541376 | |  | |  |
| 5 | | BC000560 | | KBTBD6 | | kelch repeat and BTB (POZ) domain containing 6 |
| 5 | | NM_002625 | | PFKFB1 | | 6-phosphofructo-2-kinase/fructose-2,6-biphosphatase 1 |
| 5 | | AK024491 | |  | |  |
| 5 | | NM_004737 | | LARGE | | like-glycosyltransferase |
| 5 | | NM_004583 | | RAB5C | | RAB5C, member RAS oncogene family |
| 5 | | NM_024123 | | LY6G6E | | lymphocyte antigen 6 complex, locus G6E |
| 5 | | AK056401 | |  | | CDNA FLJ31839 fis, clone NT2RP7000086 |
| 5 | | NM_001276 | | CHI3L1 | | chitinase 3-like 1 (cartilage glycoprotein-39) |
| 5 | | AK054825 | |  | | CDNA FLJ30263 fis, clone BRACE2002606 |
| 5 | | NM_015859 | | GTF2A1 | | general transcription factor IIA, 1, 19/37kDa |
| 5 | | NM_031298 | | MGC2963 | | hypothetical protein MGC2963 |
| 5 | | NM_004719 | | SFRS2IP | | splicing factor, arginine/serine-rich 2, interacting protein |
| 5 | | NM_007053 | | CD160 | | CD160 antigen |
| 5 | | NM_000761 | | CYP1A2 | | cytochrome P450, family 1, subfamily A, polypeptide 2 |
| 5 | | NM_014787 | |  | |  |
| 5 | | NM_003311 | | PHLDA2 | | pleckstrin homology-like domain, family A, member 2 |
| 5 | | AJ012755 | | LOC220594 | | TL132 protein |
| 5 | | AK056887 | |  | |  |
| 5 | | NM_006540 | | NCOA2 | | nuclear receptor coactivator 2 |
| 5 | | AL360137 | |  | | MRNA* cDNA DKFZp686A1429 (from clone DKFZp686A1429) |
| 5 | | NM_025009 | | FLJ13621 | | hypothetical protein FLJ13621 |
| 5 | | NM_018840 | | C20orf24 | | chromosome 20 open reading frame 24 |
| 5 | | AK026434 | |  | |  |
| 5 | | AK025156 | |  | | CDNA FLJ46295 fis, clone TESTI4034973 |
| 5 | | NM_004752 | | GCM2 | | glial cells missing homolog 2 (Drosophila) |
| 5 | | NM_003073 | | SMARCB1 | | SWI/SNF related, matrix associated, actin dependent regulator of chromatin, subfamily b, member 1 |
| 5 | | AL049998 | | PIK3C2A | | phosphoinositide-3-kinase, class 2, alpha polypeptide |
| 5 | | AB020704 | | PPFIA4 | | protein tyrosine phosphatase, receptor type, f polypeptide (PTPRF), interacting protein (liprin), alpha 4 |
| 6 | | AL122055 | | CDK11 | | cyclin-dependent kinase (CDC2-like) 11 |
| 6 | | AK022421 | |  | | CDNA FLJ12359 fis, clone MAMMA1002355 |
| 6 | | NM_006521 | | TFE3 | | transcription factor binding to IGHM enhancer 3 |
| 6 | | NM_017792 | |  | | Hypothetical protein FLJ20373 (FLJ20373), mRNA |
| 6 | | NM_001721 | | BMX | | BMX non-receptor tyrosine kinase |
| 6 | | NM_006069 | | MRVI1 | | murine retrovirus integration site 1 homolog |
| 6 | | NM_031922 | | REPS1 | | RALBP1 associated Eps domain containing 1 |
| 6 | | AK021462 | | MGC20741 | | ubiquitin specific protease 49 |
| 6 | | AK025842 | | NR2F2 | | nuclear receptor subfamily 2, group F, member 2 |
| 6 | | NM_004514 | | FOXK2 | | forkhead box K2 |
| 6 | | NM_006577 | | B3GNT1 | | UDP-GlcNAc:betaGal beta-1,3-N-acetylglucosaminyltransferase 1 |
| 6 | | NM_020638 | | FGF23 | | fibroblast growth factor 23 |
| 6 | | NM_021077 | | NMB | | neuromedin B |
| 6 | | AK055341 | |  | | CDNA FLJ30779 fis, clone FEBRA2000815 |
| 6 | | NM_002099 | | GYPA | | glycophorin A (includes MN blood group) |
| 6 | | AK025100 | | SNTB1 | | syntrophin, beta 1 (dystrophin-associated protein A1, 59kDa, basic component 1) |
| 6 | | AK024152 | |  | | CDNA FLJ14090 fis, clone MAMMA1000264 |
| 6 | | NM_015681 | | EPPB9 | | B9 protein |
| 6 | | AK023380 | |  | | MRNA* cDNA DKFZp686I04101 (from clone DKFZp686I04101) |
| 6 | | AB033010 | | MR-1 | | myofibrillogenesis regulator 1 |
| 6 | | AB067505 | | GALNT13 | | UDP-N-acetyl-alpha-D-galactosamine:polypeptide N-acetylgalactosaminyltransferase 13 (GalNAc-T13) |
| 6 | | BC012903 | | PPA2 | | inorganic pyrophosphatase 2 |
| 6 | | BC010437 | | MGC22679 | | hypothetical protein MGC22679 |
| 6 | | AB011537 | | SLIT1 | | slit homolog 1 (Drosophila) |
| 6 | | NM_004190 | | LIPF | | lipase, gastric |
| 6 | | NM_016426 | | GTSE1 | | G-2 and S-phase expressed 1 |
| 6 | | NM_020359 | | PLSCR2 | | phospholipid scramblase 2 |
| 6 | | AK057753 | |  | | RGS8 mRNA, partial sequence |
| 6 | | NM_005424 | | TIE | | tyrosine kinase with immunoglobulin and epidermal growth factor homology domains |
| 6 | | NM_032319 | | C2orf7 | | chromosome 2 open reading frame 7 |
| 6 | | NM_004271 | | LY86 | | lymphocyte antigen 86 |
| 6 | | U83115 | | AIM1 | | absent in melanoma 1 |
| 6 | | NM_006685 | | PROL3 | | proline rich 3 |
| 6 | | AB046770 | | PLXNA4 | | plexin A4 |
| 7 | | NM_012345 | | NUFIP1 | | nuclear fragile X mental retardation protein interacting protein 1 |
| 7 | | NM_030621 | | DICER1 | | Dicer1, Dcr-1 homolog (Drosophila) |
| 7 | | AK055327 | | FAP | | fibroblast activation protein, alpha |
| 7 | | AF155510 | | HPSE | | heparanase |
| 7 | | NM_012436 | | SPAG8 | | sperm associated antigen 8 |
| 7 | | AF052090 | | NNT | | nicotinamide nucleotide transhydrogenase |
| 7 | | AL359602 | |  | |  |
| 7 | | NM_004297 | | GNA14 | | guanine nucleotide binding protein (G protein), alpha 14 |
| 7 | | NM_016592 | | GNAS | | GNAS complex locus |
| 7 | | AF339822 | | FLT1 | | fms-related tyrosine kinase 1 (vascular endothelial growth factor/vascular permeability factor receptor) |
| 7 | | NM_017964 | | SLC30A6 | | solute carrier family 30 (zinc transporter), member 6 |
| 7 | | NM_021219 | | JAM2 | | junctional adhesion molecule 2 |
| 7 | | AK024321 | |  | | CDNA FLJ14259 fis, clone PLACE1001076 |
| 7 | | AL050107 | | TAZ | | transcriptional co-activator with PDZ-binding motif (TAZ) |
| 7 | | BG163465 | | HIC | | I-mfa domain-containing protein |
| 7 | | NM_005456 | | MAPK8IP1 | | mitogen-activated protein kinase 8 interacting protein 1 |
| 7 | | NM_006086 | | TUBB4 | | tubulin, beta, 4 |
| 7 | | NM_001260 | | CDK8 | | cyclin-dependent kinase 8 |
| 7 | | AF247167 | | AD031 | | AD031 protein |
| 7 | | AK055270 | |  | | MRNA* cDNA DKFZp686E16168 (from clone DKFZp686E16168) |
| 7 | | NM_005523 | | HOXA11 | | homeo box A11 |
| 7 | | AK056707 | | KIAA0460 | | KIAA0460 protein |
| 7 | | AK026873 | | LOC284591 | | hypothetical protein LOC284591 |
| 7 | | AF442729 | | MDAC1 | | MDAC1 |
| 7 | | NM_005901 | | MADH2 | | MAD, mothers against decapentaplegic homolog 2 (Drosophila) |
| 7 | | NM_014520 | | MYBBP1A | | MYB binding protein (P160) 1a |
| 7 | | NM_015230 | | CENTD1 | | centaurin, delta 1 |
| 7 | | AB046764 | | NBEA | | neurobeachin |
| 7 | | NM_005279 | | GPR1 | | G protein-coupled receptor 1 |
| 7 | | NM_006042 | | HS3ST3A1 | | heparan sulfate (glucosamine) 3-O-sulfotransferase 3A1 |
| 7 | | AK056352 | | PRDM5 | | PR domain containing 5 |
| 7 | | AK026679 | |  | | CDNA: FLJ23026 fis, clone LNG01738 |
| 7 | | BC008384 | |  | |  |
| 7 | | AK023457 | | RAP1A | | RAP1A, member of RAS oncogene family |
| 7 | | AB029394 | | FLJ12287 | | hypothetical protein FLJ12287 similar to semaphorins |
| 7 | | NM_002401 | | MAP3K3 | | mitogen-activated protein kinase kinase kinase 3 |
| 7 | | AF131841 | |  | | Clone 24974 mRNA sequence |
| 7 | | NM_005420 | | SULT1E1 | | sulfotransferase family 1E, estrogen-preferring, member 1 |
| 7 | | NM_004125 | | GNG10 | | guanine nucleotide binding protein (G protein), gamma 10 |
| 7 | | NM_006603 | | STAG2 | | stromal antigen 2 |
| 7 | | AK001120 | |  | | CDNA FLJ10258 fis, clone HEMBB1000908 |
| 7 | | AF353674 | | BTBD6 | | BTB (POZ) domain containing 6 |
| 7 | | AK055053 | | SHMT2 | | serine hydroxymethyltransferase 2 (mitochondrial) |
| 7 | | Z46376 | |  | |  |
| 7 | | NM_014898 | |  | |  |
| 7 | | BC008352 | |  | | Similar to hypothetical protein D630003M21 (LOC389256), mRNA |
| 7 | | AK057785 | | FLJ25056 | | hypothetical protein FLJ25056 |
| 7 | | J05158 | | CPN2 | | carboxypeptidase N, polypeptide 2, 83kD |
| 7 | | NM_000824 | | GLRB | | glycine receptor, beta |
| 7 | | NM_000086 | | CLN3 | | ceroid-lipofuscinosis, neuronal 3, juvenile (Batten, Spielmeyer-Vogt disease) |
| 7 | | AK021817 | |  | | CDNA FLJ11755 fis, clone HEMBA1005593 |
| 7 | | NM_004697 | | PRPF4 | | PRP4 pre-mRNA processing factor 4 homolog (yeast) |
| 7 | | AK057828 | | FRMD4 | | FERM domain containing 4 |
| 7 | | AK056545 | | FLJ21156 | | hypothetical protein FLJ21156 |
| 7 | | NM_024409 | | NPPC | | natriuretic peptide precursor C |
| 8 | | NM_003821 | | RIPK2 | | receptor-interacting serine-threonine kinase 2 |
| 8 | | NM_012198 | | GCA | | grancalcin, EF-hand calcium binding protein |
| 8 | | AK057491 | | USP31 | | ubiquitin-specific proteinase 31 |
| 8 | | NM_000600 | | IL6 | | interleukin 6 (interferon, beta 2) |
| 8 | | AF070674 | | BIRC3 | | baculoviral IAP repeat-containing 3 |
| 8 | | NM_006291 | | TNFAIP2 | | tumor necrosis factor, alpha-induced protein 2 |
| 8 | | NM_024960 | | PANK2 | | pantothenate kinase 2 (Hallervorden-Spatz syndrome) |
| 8 | | NM_001530 | | HIF1A | | hypoxia-inducible factor 1, alpha subunit (basic helix-loop-helix transcription factor) |
| 8 | | NM_002089 | | CXCL2 | | chemokine (C-X-C motif) ligand 2 |
| 8 | | NM_002982 | | CCL2 | | chemokine (C-C motif) ligand 2 |
| 8 | | AF327403 | | SLC25A28 | | solute carrier family 25, member 28 |
| 8 | | NM_000576 | | IL1B | | interleukin 1, beta |
| 8 | | NM_006058 | | TNIP1 | | TNFAIP3 interacting protein 1 |
| 8 | | NM_004556 | | NFKBIE | | nuclear factor of kappa light polypeptide gene enhancer in B-cells inhibitor, epsilon |
| 8 | | NM_005195 | | KIAA0146 | | KIAA0146 protein |
| 8 | | NM_004591 | | CCL20 | | chemokine (C-C motif) ligand 20 |
| 8 | | NM_002266 | | KPNA2 | | karyopherin alpha 2 (RAG cohort 1, importin alpha 1) |
| 8 | | NM_014963 | |  | |  |
| 8 | | AK055872 | | HP | | haptoglobin |
| 8 | | AB033102 | | KIAA1276 | | KIAA1276 protein |
| 8 | | D38169 | | ITPKC | | inositol 1,4,5-trisphosphate 3-kinase C |
| 8 | | NM_001418 | | EIF4G2 | | eukaryotic translation initiation factor 4 gamma, 2 |
| 8 | | NM_006290 | | TNFAIP3 | | tumor necrosis factor, alpha-induced protein 3 |
| 8 | | X52015 | | IL1RN | | interleukin 1 receptor antagonist |
| 8 | | NM_031314 | | HNRPC | | heterogeneous nuclear ribonucleoprotein C (C1/C2) |
| 8 | | NM_003900 | | SQSTM1 | | sequestosome 1 |
| 8 | | NM_002350 | | LYN | | v-yes-1 Yamaguchi sarcoma viral related oncogene homolog |
| 8 | | AL161952 | | GLUL | | glutamate-ammonia ligase (glutamine synthase) |
| 8 | | NM_002984 | | CCL4 | | chemokine (C-C motif) ligand 4 |
| 8 | | NM_002999 | | SDC4 | | syndecan 4 (amphiglycan, ryudocan) |
| 8 | | NM_001078 | | VCAM1 | | vascular cell adhesion molecule 1 |
| 8 | | NM_000201 | | ICAM1 | | intercellular adhesion molecule 1 (CD54), human rhinovirus receptor |
| 8 | | NM_003407 | | ZFP36 | | zinc finger protein 36, C3H type, homolog (mouse) |
| 8 | | NM_022154 | | SLC39A8 | | solute carrier family 39 (zinc transporter), member 8 |
| 8 | | NM_001300 | | COPEB | | core promoter element binding protein |
| 8 | | NM_001816 | | CEACAM8 | | carcinoembryonic antigen-related cell adhesion molecule 8 |
| 8 | | NM_002356 | | MARCKS | | myristoylated alanine-rich protein kinase C substrate |
| 8 | | NM_020529 | | NFKBIA | | nuclear factor of kappa light polypeptide gene enhancer in B-cells inhibitor, alpha |
| 8 | | BI821590 | | LOC92017 | | similar to RIKEN cDNA 4933437K13 |
| 8 | | NM_001133 | | AFM | | afamin |
| 8 | | AK025431 | |  | | CDNA: FLJ21778 fis, clone HEP00201 |
| 8 | | NM_000270 | | NP | | nucleoside phosphorylase |
| 8 | | NM_003330 | | TXNRD1 | | thioredoxin reductase 1 |
| 8 | | NM_001215 | | CA6 | | carbonic anhydrase VI |
| 8 | | NM_015675 | | GADD45B | | growth arrest and DNA-damage-inducible, beta |
| 8 | | AL359600 | | ABCC5 | | ATP-binding cassette, sub-family C (CFTR/MRP), member 5 |
| 8 | | NM_013448 | | BAZ1A | | bromodomain adjacent to zinc finger domain, 1A |
| 8 | | D31887 | | SLC39A14 | | solute carrier family 39 (zinc transporter), member 14 |
| 8 | | NM_014399 | | TM4SF13 | | transmembrane 4 superfamily member 13 |
| 9 | | AF339799 | |  | | Clone IMAGE:2363394, mRNA sequence |
| 9 | | NM_002818 | | PSME2 | | proteasome (prosome, macropain) activator subunit 2 (PA28 beta) |
| 9 | | NM_001565 | | CXCL10 | | chemokine (C-X-C motif) ligand 10 |
| 9 | | NM_005409 | | CXCL11 | | chemokine (C-X-C motif) ligand 11 |
| 9 | | NM_005892 | | FMNL1 | | formin-like 1 |
| 9 | | NM_000759 | | CSF3 | | colony stimulating factor 3 (granulocyte) |
| 9 | | AK056767 | |  | | Hypothetical protein FLJ32205 (FLJ32205), mRNA |
| 9 | | NM_000542 | | SFTPB | | surfactant, pulmonary-associated protein B |
| 9 | | AF139768 | | CLECSF5 | | C-type (calcium dependent, carbohydrate-recognition domain) lectin, superfamily member 5 |
| 9 | | NM_004159 | | PSMB8 | | proteasome (prosome, macropain) subunit, beta type, 8 (large multifunctional protease 7) |
| 9 | | NM_002965 | | S100A9 | | S100 calcium binding protein A9 (calgranulin B) |
| 9 | | AF020774 | |  | | Similar to arachidonate lipoxygenase, epidermal, clone IMAGE:5270337, mRNA |
| 9 | | NM_004120 | | GBP2 | | guanylate binding protein 2, interferon-inducible |
| 9 | | NM_000636 | | SOD2 | | superoxide dismutase 2, mitochondrial |
| 9 | | NM_015907 | | LAP3 | | leucine aminopeptidase 3 |
| 9 | | AL390143 | |  | | MRNA* cDNA DKFZp547N074 (from clone DKFZp547N074) |
| 9 | | AK000852 | | LOC348262 | | hypothetical protein LOC348262 |
| 9 | | NM_002036 | | FY | | Duffy blood group |
| 9 | | NM_014770 | | CENTG1 | | centaurin, gamma 1 |
| 9 | | NM_002954 | | RPS27A | | ribosomal protein S27a |
| 9 | | AF323540 | | APOL1 | | apolipoprotein L, 1 |
| 9 | | NM_052889 | | COP | | CARD only protein |
| 9 | | NM_003250 | | THRA | | thyroid hormone receptor, alpha (erythroblastic leukemia viral (v-erb-a) oncogene homolog, avian) |
| 9 | | NM_002964 | | S100A8 | | S100 calcium binding protein A8 (calgranulin A) |
| 9 | | AY026763 | | TRIM47 | | tripartite motif-containing 47 |
| 9 | | NM_002727 | | PRG1 | | proteoglycan 1, secretory granule |
| 9 | | NM_002198 | | IRF1 | | interferon regulatory factor 1 |
| 9 | | NM_002053 | | GBP1 | | guanylate binding protein 1, interferon-inducible, 67kDa |
| 9 | | NM_005564 | | LCN2 | | lipocalin 2 (oncogene 24p3) |
| 9 | | NM_002219 | |  | |  |
| 10 | | NM_003370 | | VASP | | vasodilator-stimulated phosphoprotein |
| 10 | | NM_021129 | | PP | | pyrophosphatase (inorganic) |
| 10 | | NM_013417 | | IARS | | isoleucine-tRNA synthetase |
| 10 | | NM_001085 | | SERPINA3 | | serine (or cysteine) proteinase inhibitor, clade A (alpha-1 antiproteinase, antitrypsin), member 3 |
| 10 | | D90145 | |  | |  |
| 10 | | AL117643 | | ACVR1B | | activin A receptor, type IB |
| 10 | | NM_004529 | | MLLT3 | | myeloid/lymphoid or mixed-lineage leukemia (trithorax homolog, Drosophila)* translocated to, 3 |
| 10 | | NM_005622 | | SAH | | SA hypertension-associated homolog (rat) |
| 10 | | AL137597 | | C20orf110 | | chromosome 20 open reading frame 110 |
| 10 | | NM_006637 | |  | |  |
| 10 | | NM_005734 | | HIPK3 | | homeodomain interacting protein kinase 3 |
| 10 | | AK026497 | | YY1 | | YY1 transcription factor |
| 10 | | NM_001993 | | F3 | | coagulation factor III (thromboplastin, tissue factor) |
| 10 | | NM_002075 | | GNB3 | | guanine nucleotide binding protein (G protein), beta polypeptide 3 |
| 10 | | BC018700 | | FLJ20400 | | CTF8, chromosome transmission fidelity factor 8 homolog (S. cerevisiae) |
| 10 | | NM_002189 | | IL15RA | | interleukin 15 receptor, alpha |
| 10 | | NM_025097 | | FLJ21106 | | hypothetical protein FLJ21106 |
| 10 | | BC011671 | | GNB5 | | guanine nucleotide binding protein (G protein), beta 5 |
| 10 | | NM_014365 | | HSPB8 | | heat shock 27kDa protein 8 |
| 10 | | NM_033051 | | TSCOT | | thymic stromal co-transporter |
| 10 | | NM_002408 | | MGAT2 | | mannosyl (alpha-1,6-)-glycoprotein beta-1,2-N-acetylglucosaminyltransferase |
